# A Comparison of *Ex Vivo* Expanded Human Regulatory T Cells Using Allogeneic Stimulated B Cells or Monocyte-Derived Dendritic Cells

**DOI:** 10.3389/fimmu.2021.679675

**Published:** 2021-06-18

**Authors:** Linda M. Lee, Hong Zhang, Karim Lee, Horace Liang, Alexander Merleev, Flavio Vincenti, Emanual Maverakis, Angus W. Thomson, Qizhi Tang

**Affiliations:** ^1^ Department of Surgery, University of California San Francisco, San Francisco, CA, United States; ^2^ Starzl Transplantation Institute, University of Pittsburgh, Pittsburgh, PA, United States; ^3^ Department of Dermatology, School of Medicine, University of California Davis, Davis, CA, United States; ^4^ Department of Medicine, University of California San Francisco, San Francisco, CA, United States; ^5^ Department of Immunology, University of Pittsburgh, Pittsburgh, PA, United States

**Keywords:** immune regulation, regulatory T cell, Treg therapy, dendritic cells, B cells, human, transplantation, transplant tolerance

## Abstract

Alloreactive regulatory T cells (arTregs) are more potent than polyclonal Tregs at suppressing immune responses to transplant antigens. Human arTregs can be expanded with allogeneic CD40L-stimulated B cells (sBcs) or stimulated-matured monocyte-derived dendritic cells (sDCs). Here, we compared the expansion efficiency and properties of arTregs stimulated *ex vivo* using these two types of antigen-presenting cells. Compared to sBcs, sDCs stimulated Tregs to expand two times more in number. The superior expansion-inducing capacity of sDCs correlated with their higher expression of CD80, CD86, and T cell-attracting chemokines. sBc- and sDC-arTregs expressed comparable levels of FOXP3, HELIOS, CD25, CD27, and CD62L, demethylated FOXP3 enhancer and *in vitro* suppressive function. sBc- and sDCs-arTregs had similar gene expression profiles that were distinct from primary Tregs. sBc- and sDC-arTregs exhibited similar low frequencies of IFN-γ, IL-4, and IL-17A-producing cells, and the cytokine-producing arTregs expressed high levels of FOXP3. Almost all sBc- and sDC-arTregs expressed CXCR3, which may enable them traffic to inflammatory sites. Thus, sDCs-arTregs that expand more readily, are phenotypically similar to sBc-arTregs, supporting sDCs as a viable alternative for arTreg production for clinical evaluation.

## Introduction

Organ transplantation can dramatically decrease morbidity and mortality, and improve the quality of life for patients with end-stage organ disease. However, in the process, the recipient’s immune system is activated against donor alloantigens, leading to graft injury and potential graft loss (1). A combination of immunosuppressive drugs is currently used as standard therapy to prevent graft injury (2). However, the use of the current drugs to obtain optimum immune suppression is often limited by their toxicities. These drugs can enhance susceptibility to infection, injure organs *via* non-immune cell toxicities, and predispose individuals to development of cancer (2). For example, corticosteroids are toxic to pancreatic islets and can cause post-transplant diabetes (3). Calcineurin inhibitors exhibit nephrotoxicity and can consequently decrease the life of kidney grafts or impair renal function in recipients of other types of organ transplant (4). Also, corticosteroids and calcineurin inhibitors lead to frequent occurrence of metabolic (5) and neurologic (6) side effects, which have major impacts on the quality of life on the recipients of solid organ transplants.

Promoting immune tolerance to transplanted organs can potentially decrease or eliminate the use of immunosuppressive drugs. Several early phase regulatory T cell (Treg) therapy trials in transplantation have been initiated (7). In preclinical murine models, donor alloreactive-Tregs (arTregs) are 5-10 times more effective compared to polyclonal Tregs in reducing the number of anti-donor alloreactive T effector cells. In current clinical trials for solid organ transplantation, arTregs are being expanded using either irradiated donor PBMCs or donor-derived CD40L-stimulated B cells (sBcs) as antigen-presenting cells (APCs) (7). However, arTreg expansions can be highly variable (7), thus optimizing any aspect of the arTreg manufacturing process would be beneficial. Dendritic cells (DCs) are potent APCs that can expand arTregs (8–10). However, no study of our knowledge has directly compared Tregs activated by allogeneic B cells versus allogeneic DCs to determine what are the similarities and differences between these two approaches.

In this study, we compared the arTreg-stimulating capacity of human stimulated matured monocyte-derived DCs (sDCs) differentiated from CD14^+^ blood monocytes, to that of sBcs to determine whether sDCs can potentially be used as an alternative APC to sBc for arTreg expansion.

## Materials and Methods

### Cells

PBMCs from normal donors were isolated as previously described (11) under appropriate UCSF (Study 10-01980) and University of Pittsburgh (Study 19120084) institutional review board approval. Cells from males and females were used for all experiments, except only cells from females were used in the Treg-specific demethylated region (TSDR) methylation assay.

### CD40L-Stimulated B Cells and Stimulated Matured Monocyte-Derived DCs

sBcs were generated as previously described using CD40L-expressing K562 cells (12). Cytokine-matured sDCs were used for all experiments, except monophosphoryl lipid A (MPLA)-matured sDCs were used in responder:stimulator combination 3 in the T cell receptor sequencing analysis. Cytokine-matured sDCs were generated from PBMC-isolated CD14^+^ monocytes using the ImmunoCult™ Dendritic Cell Culture Kit (StemCell Technologies). Briefly, the kit contains a proprietary maturation supplement that includes TNFα and IL-1β. MPLA-matured sDCs were generated by differentiating monocytes in human recombinant (rh) IL-4- and rhGM-CSF-supplemented medium followed by maturation with MPLA. Prior to all assays, sBcs and sDCs were irradiated (25 Gray).

### Mixed Leukocyte Reaction

Carboxyfluorescein diacetate, succinimidyl ester (CFSE, Invitrogen/Thermo Fisher Scientific)-labeled responder PBMCs were cultured at 37°C with irradiated allogeneic sBcs (2 sBcs per PBMC) or sDCs (1 sDC per 4 PBMCs) for 4 days. CFSE dilution/proliferation was assessed by flow cytometry.

### T Cell Culture

Tregs (CD4^+^CD127^lo/-^CD25^+^) and conventional T cells (Tconvs; CD4^+^CD127^+^CD25^-^) from responder PBMCs were FACS-purified using a FACS Aria II (BD Biosciences, San Jose, CA). The T cells were cultured with irradiated allogeneic sBcs (4 sBcs per 1 T cell) or sDCs (1 sDC per 4 T cells) in Optimizer T cell expansion media (Invitrogen), supplemented with rhIL-2 (300 IU/ml) (Proleukin, Novartis) at 37°C. Alloreactive T cell phenotypes were assessed on day 11.

### sBc and sDC Cytokine Production

sBcs and sDCs were cultured alone at 37°C at the same density as used in T cell expansion cultures (200,000 sBcs or 12,500 sDCs in 100 µL assay medium). After 48 h, supernatants were harvested, and cytokine and chemokine levels were measured using 65-plex human cytokine/chemokine Luminex assay (Eve Technologies, Alberta, Canada).

### Flow Cytometry

sBcs, sDCs, MLR cultures, and T cells were stained with antibodies against cell surface molecules, and, for some experiments, followed by intracellular stain for transcription factors and/or cytokines after fixation and permeabilization with Foxp3/Transcription Factor Staining Buffer Set (Invitrogen). Samples were analyzed on a BD Fortessa, BD LSRII or a Beckman Coulter Navios flow cytometer (Indianapolis, IN). Data analyses were performed using FlowJo (TreeStar, Ashland, OR) or Kaluza Analysis Software (Beckman Coulter). Precursor frequencies were calculated as previously described (13).

### Treg-Specific Demethylated Region Methylation Assay

Frozen cell pellets were analyzed using the human FOXP3, Intron 1 TSDR region assay (EpigenDX, Hopkington, MA, ADS783-FS2) to obtain percentages of demethylated TSDR. All samples were from female donors. Due to X-chromosome inactivation in females, the maximum percentage of demethylation is ~50%.

### Gene Expression Analysis of Stimulated T Cells

FACS-purified Tregs and Tconvs (primary-Tregs and primary-Tconvs) and expanded alloreactive T cells were stimulated with Dynabeads Human T-Activator CD3/CD28 beads (Invitrogen) for 24 h. RNA was isolated and analyzed using the Nanostring PanCancer Immune Profiling Panel (Seattle, WA). Nanostring data was analyzed using the nSolver 4.0 software.

### Cytokine Analyses of Stimulated T Cells

For analysis of secreted cytokines, primary T cells and cultured alloreactive T cells were stimulated with anti-CD3/CD28 beads for 24 h. Supernatants were harvested and analyzed for cytokines and chemokines using a 42-plex Luminex assay (Eve Technologies). For analysis of intracellular cytokines, primary T cells and expanded alloreactive T cells were stimulated with PMA and ionomycin (Sigma Aldrich) in the presence of Brefeldin A (Sigma Aldrich) and monensin (BD Biosciences) for 5 h before staining and analysis using flow cytometry.

### TCR Sequencing

RNA was isolated from ~5x10^5^ arTregs using High Pure Isolation Kit (Roche Life Sciences, Indianapolis, IN) and submitted to iRepertoire (Huntsville, AL) for TCRβ sequencing and data analysis. Approximately 250,000 cell equivalent RNA was sequenced, which yielded ~1x10^6^ reads after applying filters to eliminate sequencing artifacts per iRepertoire protocol. MiXCR software was used for TCR repertoire comparison and data visualization (14–16). Scripts developed in R were used to aggregate clones, plot data, and to calculate percentages of shared reads and unique CDR3s, and Jaccard and Morisita distances (17). Briefly, for each individual sample well, first, T cell clones with the same CDR3 amino acid sequence were merged. Second, the public clones (clone that is present in 2 or more samples) were extracted from the sample. Third, then the top 100 clones were extracted from the samples. Lastly, the filtered samples were compared with other samples. For digitally pooled samples, replicate well data were combined, then the filtering steps described for individual sample wells were performed.

### *In Vitro* Suppression Assay

Responder PBMCs were cultured with irradiated stimulator PBMCs, in the presence of sBc- or sDC-arTregs for 7 days. ^3^[H] thymidine (Perkin Elmer, Waltham, MA) was added for the final 16 h of culture. The arTregs tested were generated from the same donor as the responder PBMCs. sBcs or sDCs tested were generated from the same donor as the stimulator PBMCs. Additionally, third-party donors were used to assess specificity to the sBc and sDC donors. Proliferation was assessed using ^3^[H] thymidine incorporation in triplicate wells and quantified as counts per minute (cpm). Percent suppression was calculated using the following formula = (1- [(mean cpm of wells with Tregs)/(mean cpm of wells with no Tregs)]) x100.

### Statistics

Statistics were performed using GraphPad Prism, version 5 or 6 (GraphPad Software, San Diego, CA). Briefly, for most experiments, Wilcoxon matched-pairs signed rank test was used to compare sBcs versus sDCs, sBc-arTregs versus sDC-arTregs, and cytokine-producing cells versus non-cytokine-producing cells. In the Treg suppression assay, unpaired t-test was used to compare sBc-arTregs and sDC-arTregs at the same Treg dilution. Data from primary T cells and arTconvs are shown as a reference and were not included in statistical analyses.

## Results

### Both sBcs and sDCs Are Potent Allogeneic T Cell Stimulators

**Figure 1:**
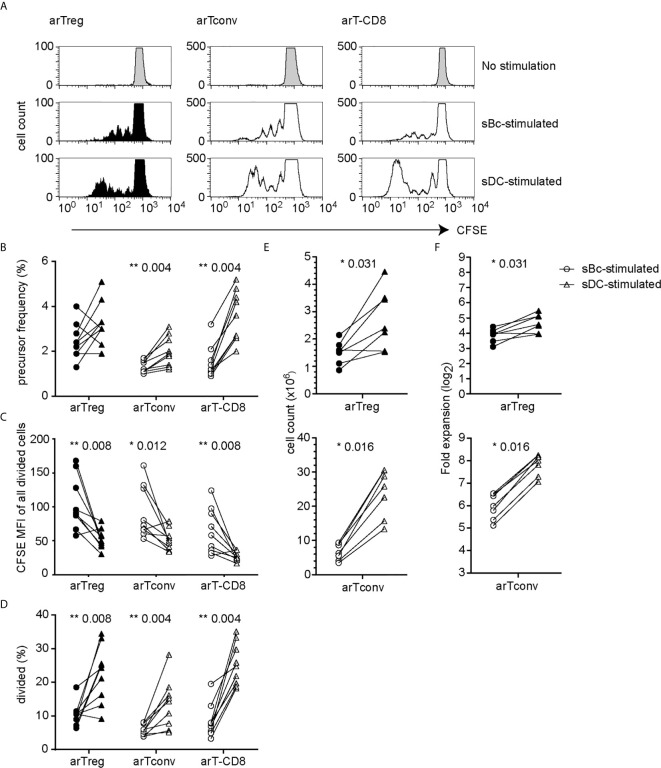
sDCs, compared to sBcs, stimulate more alloreactive T cell proliferation. **(A–D)** CFSE-labeled responder PBMCs were stimulated with sBcs (2 sBcs per 1 PBMC), or sDCs (1 sDC per 4 PBMCs) for 4 days. **(A)** Representative histograms showing CFSE dilution/proliferation of T cells: Tregs (CD3^+^CD4^+^CD8^-^FOXP3^+^HELIOS^+^), Tconvs (CD3^+^CD4^+^CD8^-^non-Treg), and CD8^+^ T cells (CD3^+^CD8^+^CD4^-^). Histograms are zoomed in to show proliferation peaks. **(B)** T cell precursor frequency. **(C)** Divided T cells were gated as CFSE^lo^, and CFSE level of expression was measured by mean fluorescence intensity (MFI). **(D)** Percentage of divided T cells (CFSE^lo^) of total T cells. **(E, F)** FACS-purified Tregs (CD4^+^CD127^lo/-^CD25^+^) and Tconvs (CD4^+^CD127^+^CD25^-^) were cultured with sBcs (4 sBcs per 1 T cell), or sDCs (1 sDC per 4 T cells). arTreg (top) and arTconv (bottom) **(E)** cell counts on d11 and **(F)** fold expansion from d0 to d11. Cell counts were normalized to d0 count of 100,000. Data in **(A–D)** contain 9 different responder-stimulator combinations. Data in **(E, F)** contain 7 different responder-stimulator combinations. Connecting lines indicate alloreactive T cells stimulated by APCs (sBcs or sDCs) derived from the same donor. Statistics were performed using the Wilcoxon matched-pairs signed rank test.

We first compared the ability of sBcs and sDCs to stimulate allogeneic T cells by culturing mixed leukocyte reaction (MLR) cultures containing CFSE-labeled PBMCs with allogeneic sBcs or sDCs (**Figure 1A**). Previously, we found 2 sBcs per responding PBMC, and 4 sBcs per responding purified human Treg, were optimal ratios to stimulate T cell expansion (12). In contrast, one DC can stimulate 1-10 Tregs (9, 10). In pilot studies, we determined that 1:4, 1:8, and 1:16 ratios of sDC : PBMCs led to similar proliferation of T cells in PBMCs (**Supplementary Figure S1**). Thus, for all experiments described hereafter, for MLR cultures, we used ratios of 1 PBMC to 2 sBcs and 4 PBMCs to 1 sDC. For stimulating T cell expansions, we used ratios of 1 T cell to 4 sBcs, and 4 T cells to 1 sDC. We next compared the ability of sBcs and sDCs to drive cell cycle progression by back-calculating the frequencies of T cells that entered cycle in the original PBMC population based on division peaks (**Figure 1B**). sDCs, compared to sBcs, promoted more alloantigen-reactive CD4^+^ T conventional cells (Tconvs), CD8^+^ T cells, and in some cases CD4^+^FOXP3^+^HELIOS^+^ Tregs to proliferate, but this was not statistically significant for Tregs. However, in most cases, responding Tconvs, CD8^+^ T cells, and Tregs divided more when stimulated with sDCs, as indicated by a shift in the CFSE division peaks to the left (**Figures 1A, C** **)**, and reflected in the increased proportions of divided T cells in sDC-stimulated cultures compared to sBc-stimulated cultures (**Figure 1D**). These data suggest that sDCs stimulate more T cells to enter the cell cycle and drive them to proliferate more in the 4-day culture. We next compared the two APCs’ abilities to expand FACS-purified CD4^+^CD127^lo/-^CD25^+^ Tregs and CD4^+^CD127^+^CD25^-^ Tconvs. On average, sDCs, compared to sBcs, induced ~2-fold higher expansion of arTregs and arTconvs (**Figures 1E, F** **)**.

**Figure 2:**
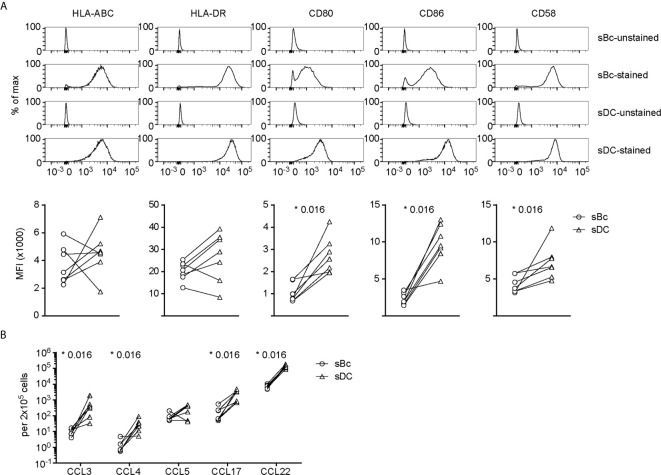
sDCs, compared to sBcs, express higher levels of costimulatory molecules and secrete more T cell-attracting chemokines. **(A)** Expression of cell surface molecules on sBcs and sDCs. Cells were stained with antibodies (“sBc-stained” and “sDC-stained”), or were not stained with antibodies (“sBc-unstained” and “sDC-unstained”) for reference. Representative histograms (top row) and level of expression (MFI) (bottom row) of cell surface molecules on sBcs and sDCs. **(B)** Secretion of chemokines by sBcs and sDCs. sBcs and sDCs were plated alone at the same density used to culture purified T cells (200,000 sBcs or 12,500 sDCs in 100µL assay medium). After 48 h, the culture supernatants were harvested and tested for the indicated molecules using Luminex assay. Data in **(A, B)** contain 7 different sBc:sDC pairs. Connecting lines indicate sBcs and sDCs derived from the same donor. Statistics were performed between sBcs and sDCs using the Wilcoxon matched-pairs signed rank test.

We then compared sBcs and sDCs to explore what features of sDCs that might explain their higher T cell stimulatory capacities. Both sBcs and sDCs expressed comparable high levels of HLA-ABC and HLA-DR (**Figure 2A**). However, sDCs expressed higher levels of CD80 and CD86 and the adhesion molecule CD58. Robust T cell expansions are usually preceded by efficient clustering of T cells with APCs, which may be facilitated by chemokines (18). sBcs and sDCs secreted similar CCL5 levels on a per cell basis, whereas sDCs produced significantly higher levels of CCL3, CCL4, CCL17, and CCL22 (**Figure 2B**). Thus, higher expression of CD80, CD86 and CD58 by sDCs and their greater chemokine production may explain the higher potency of sDCs in stimulating arTreg expansion.

### arTreg Identity and Phenotype

**Figure 3:**
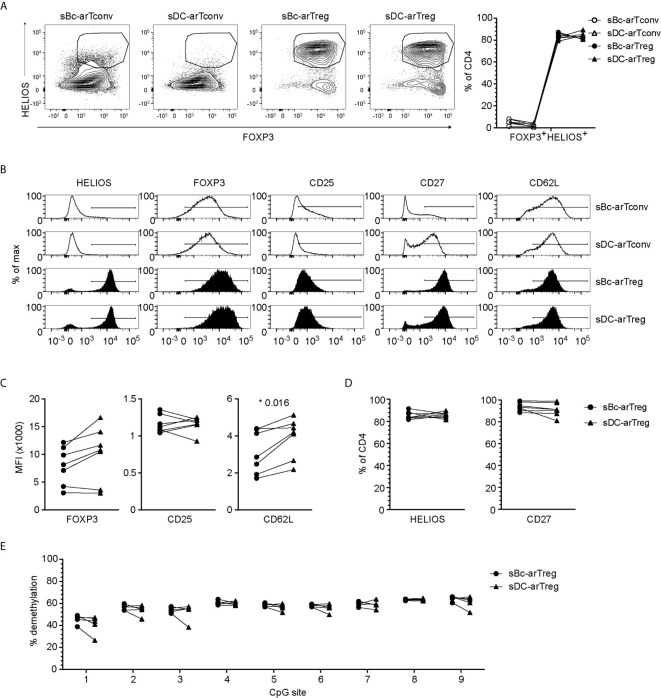
sBc- and sDC-arTregs express Treg-associated molecules and are stable Tregs. **(A–D)** Expression of different molecules by arTregs and arTconvs was assessed by flow cytometry. **(A)** Representative FOXP3 versus HELIOS contour plots of arTregs and arTconvs (left) and %FOXP3^+^HELIOS^+^ of CD4 cells (right). **(B)** Representative histograms of arTregs and arTconvs. **(C)** Level of expression (MFI) and **(D)** Percentage of CD4^+^ T cells expressing specific molecules. **(E)** Percentage of demethylated FOXP3 gene TSDR at different CpG sites. All arTregs were derived from female donors so maximum demethylation was ~50%. Data in **(A–D)** contain 7 different responder-stimulator combinations. Data in **(E)** contain 5 different responder-stimulator combinations. Connecting lines indicate alloreactive T cells stimulated by APCs (sBcs or sDCs) derived from the same donor. Statistics were performed between sBc-arTregs and sDC-arTregs using the Wilcoxon matched-pairs signed rank test. Data from arTconvs are shown as reference and were not included in statistical analyses.

Both sBc- and sDC-arTregs expressed high levels of the Treg lineage-defining transcription factor, FOXP3, and Treg-associated molecules HELIOS, CD25, CD27 and CD62L (**Figures 3A–D**). FOXP3 is also induced in arTconvs (19–21), but not expressed as highly as in arTregs (**Figures 3A–C**). A more definitive determination of Treg identity is the demethylation of the Treg-specific demethylated region (TSDR), an enhancer in the FOXP3 gene. sBc- and sDC-arTregs displayed similar high percentages of demethylated TSDR (**Figure 3E**), suggesting that both sBc and sDC expanded bona fide lineage-committed arTregs.

**Figure 4:**
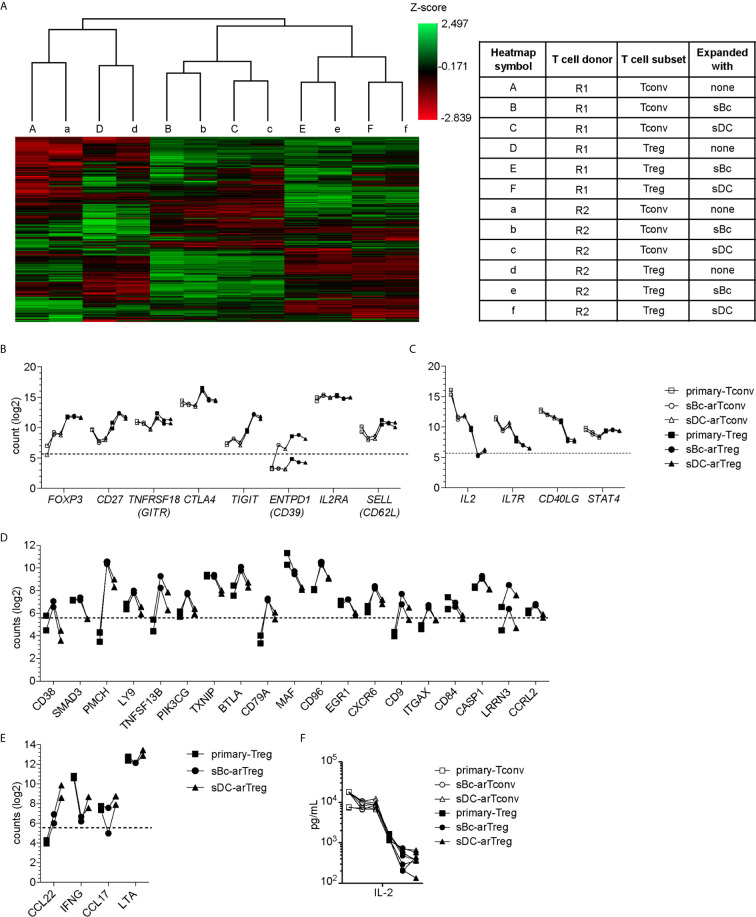
sBc- and sDC-arTregs maintain their Treg identity after restimulation. **(A–F)** FACS-purified T cells (primary-Tregs and primary-Tconvs) and cultured alloreactive T cells were stimulated with anti-CD3/CD28 beads for 24 h. **(A–E)** RNA was harvested from stimulated cells and analyzed using Nanostring’s PanCancer Immune Profiling Panel. **(A)** Heatmap generated from unsupervised clustering analysis of 439 normalized gene expression data (left). Description of T cell subsets and APC stimulation (table, right) **(B)** Gene expression of Treg-associated molecules. **(C)** Gene expression of Tconv-associated molecules. **(D)** Genes differentially expressed at least 2-fold greater in sBc-arTregs compared to sDC-arTregs. **(E)** Genes differentially expressed at least 2-fold less in sBc-arTregs compared to sDC-arTregs. **(F)** Supernatants from stimulated cells were collected and analyzed for IL-2 using Luminex assay. Data in **(A–F)** contain 2 different responder-stimulator combinations. Values above the dotted line are above background expression. Data in **(F)** contain 7 different responder-stimulator combinations. Connecting lines indicate alloreactive T cells stimulated by APCs (sBcs or sDCs) derived from the same donor. Statistics in **(F)** were performed between sBc-arTregs and sDC-arTregs using the Wilcoxon matched-pairs signed rank test. Data from primary T cells and arTconvs are shown as reference and were not included in statistical analyses.

To further probe the phenotype of the sBc- and sDC-expanded arTregs, we restimulated them with anti-CD3/CD28 beads for 24 h, then examined their gene expression using a 770-gene panel from Nanostring (**Table S1**). Unsupervised clustering analysis showed that sBc- and sDC-arTregs were most similar and distinct from arTconvs, and further separated from Tregs and Tconvs not expanded by APCs (primary Tregs and Tconvs) (**Figure 4A**). Consistent with protein expression assessed before restimulation (**Figures 3A–D**), mRNA expression of Treg-associated molecules FOXP3, CD25, CD27, and CD62L were mostly similarly expressed between sBc- and sDC-arTregs (**Figure 4B**). Additionally, sBc- and sDC-arTregs expressed mRNA encoding other Treg-associated molecules, such as GITR (glucocorticoid-induced tumor necrosis factor receptor, TNFRSF18), CTLA-4 (cytotoxic T-lymphocyte-associated protein 4), TIGIT (T Cell Immunoreceptor With Ig And ITIM Domains), and CD39 (**Figure 4B**).

Previous studies have shown that repeated *in vitro* stimulation of Tregs leads to Treg destabilization (22, 23). To assess potential arTreg destabilization after restimulation, we examined expression of molecules that are normally repressed in Tregs. Upon activation, Tconvs preferentially express CD40L compared to Tregs (24). sBc- and sDC-arTregs expressed lower levels of CD40L mRNA compared to sBc- and sDC-arTconvs (**Figure 4C**). Additionally, sBc- and sDC-arTregs expressed lower levels of IL-2 and IL-7R mRNA compared to sBc- and sDC-arTconvs, consistent with low IL-2 in arTreg culture supernatants (**Figure 4F**). Previous studies have shown that stable Tregs are characterized by lower expression of STAT4 protein compared to Tconvs (25). Interestingly, STAT4 mRNA induction was largely similar between arTregs compared to arTconvs (**Figure 4C**).

Although sBc- and sDC-arTregs expressed similar levels of Treg-associated molecules, we found 23 genes that were differentially expressed by at least 2-fold between sBc- and sDC-arTregs (**Figures 4D, E**). Notably, mRNA encoding CD38, a transmembrane cyclic ADP ribose hydrolase, was induced almost 7-fold more in sBc-arTregs compared to sDC-arTregs (**Figure 4D**). Previous studies have shown that mouse CD38^+^ Tregs are more suppressive than CD38^-^ Tregs (26), which suggest sBc-arTregs may be more suppressive than sDC-arTregs. Interestingly, we found sDC-arTregs compared to sBc-arTregs expressed almost 7-fold higher mRNA levels encoding a CCR4 ligand, CCL22 (**Figure 4E** **)**. Higher expression of CCL22 may allow sDC-arTregs attract CCR4-expressing T cells (18) to the same APC by which they have been activated. All other chemokines in the Nanostring panel either showed no difference between sBc- and sDC-arTregs (CCL1, CCL3, CCL4, CCL5, CCL17, CCL20, CCL22), or were below background (data not shown).

### arTreg Specialization

**Figure 5:**
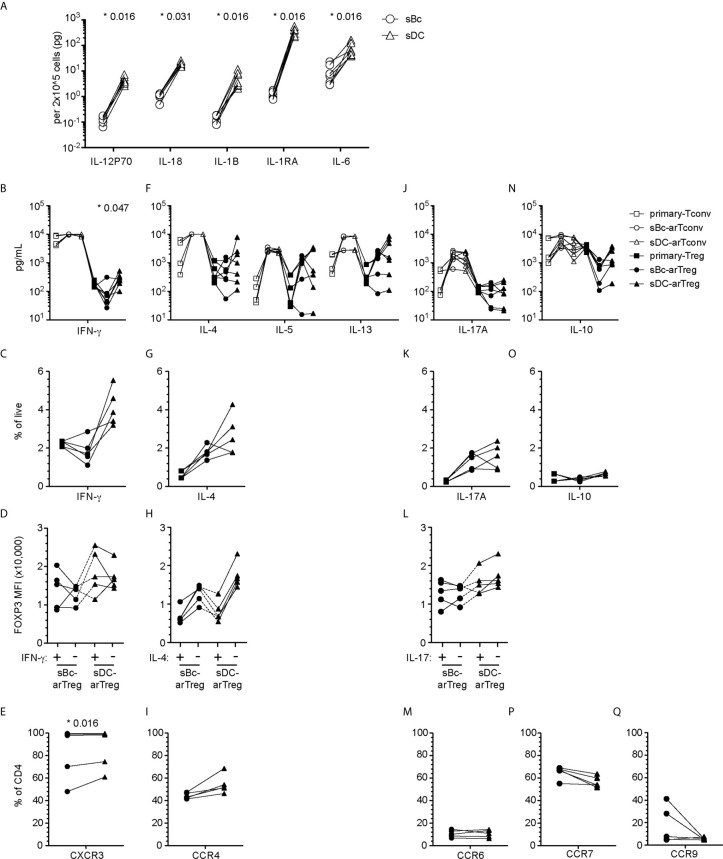
sBc- and sDC-arTregs acquire specialized characteristics while maintaining expression of FOXP3. **(A)** Secretion of cytokines by sBcs and sDCs. sBcs and sDCs were plated alone at the same density used to culture purified T cells (200,000 sBcs or 12,500 sDCs in 100µL assay medium). After 48 h, the culture supernatants were harvested and tested for the indicated molecules using Luminex assay. **(B, F, J, N)** Primary T cells and cultured alloreactive T cells were stimulated with anti-CD3/CD28 beads for 24 h. Supernatants from stimulated cells were collected and analyzed for different cytokines using Luminex assay. **(C, D, G, H, K, L, O)** Primary T cells and cultured alloreactive T cells were stimulated with PMA/Ionomycin in the presence of Brefeldin A and monensin for 5 h. Cytokine production by arTregs **(C, G, K, O)**, and level of expression (MFI) of FOXP3 in cytokine- and non-cytokine-producing arTregs **(D, H, L)** was assessed by intracellular staining. **(E, I, M, P, Q)** Chemokine receptor expression on arTregs. Data in **(A)** contain 7 different sBc and sDC pairs. Connecting lines indicate sBcs and sDCs derived from the same donor. Data in **(B, F, J, N)** contain 7 different responder:stimulator pairs. Data in **(C, D, G, H, K, L, O)** contain 5 different responder:stimulator pairs. Data in **(E, I, M, P, Q)** contain at least 5 different responder:stimulator pairs. Connecting lines indicate alloreactive T cells stimulated by APCs (sBcs or sDCs) derived from the same donor. Statistics in **(A)** were performed using the Wilcoxon matched-pairs signed test. Statistics in **(B, C, E, F, G, I, J, K, M, N, O, P, Q)** were performed comparing sBc- and sDC-arTreg populations using the Wilcoxon matched-pairs signed rank test. Data from primary Tconvs and arTconvs are shown as a reference and were not included in statistical analyses. Statistics in **(D, H, L)** were performed compared cytokine-producing and non-producing cells within the same APC stimulation group using the Wilcoxon matched-pairs signed rank test.

Tregs can specialize to suppress specific T effector cell functions (7). Specialized Tregs express transcription factors, cytokines and chemokine receptors that are associated with the CD4^+^ T effector subsets they suppress (27). The tissue microenvironment where Tregs are activated influences Treg specialization, thus we examined the cytokine secretion profile of sBcs and sDCs. sDCs, compared to sBcs, expressed higher levels of IL-1β, IL-6, IL-12p70, and IL-18 (**Figure 5A**). sDC cultures also produced more IL-1R antagonist (IL-1RA).

We then compared sBc- and sDC-arTregs for their specialization phenotypes. We first examined the molecules associated with T_H_1-like Tregs. Both sBc- and sDC-arTregs expressed less TBX21 when compared with primary Tregs and Tconvs, but sDC-arTregs expressed ~2-fold more TBX21 when compared to sBc-arTregs (**Supplementary Figure 2A**), consistent with their higher IFN-γ mRNA expression (**Supplementary Figure 2B**), IFN-γ secretion in culture supernatants (**Figure 5B**), and a trend to a greater percentage of cells producing IFN-γ detected intracellularly using flow cytometry (**Figure 5C**, **Supplementary Figure 3A**). However, IFN-γ-producing cells showed comparable FOXP3 MFI when compared with non-IFN-γ-producing cells from the sDC-arTreg cultures (**Figure 5D**, **Supplementary Figure 3B**), suggesting these arTreg are likely still bona fide Tregs. Additionally, the percentage of IFN-γ^+^ cells was lower among sBc- and sDC-arTregs when compared to sBc- and sDC-arTconvs (**Supplementary Figure 3C**). Lastly, almost all sBc- and sDC-arTregs expressed CXCR3 protein (**Figure 5E**, **Supplementary Figure 4**). These data suggest that sBc- and sDC-arTregs may be able to traffic efficiently to sites of T_H_1 inflammation and suppress T_H_1 responses more effectively than circulating Tregs.

We also examined molecules associated with T_H_17-like Tregs. Both sBc- and sDC-arTregs expressed relatively low levels of RORC **(** **Supplementary Figure 2A** **)**, secreted similar levels of IL-17A (**Figure 5J**) and comprised similar percentages of IL-17A-producing cells (**Figure 5K**, **Supplementary Figure 3A**). The level of IL-17 production was much less compared to arTconvs (**Figure 5J**, **Supplementary Figures 3A, C**). IL-17-producing cells showed comparable FOXP3 MFI when compared with non-IL-17-producing cells (**Figure 5L**, **Supplementary Figure 3B**). CCR6 mRNA expression (**Supplementary Figure 2C**) and percentages of arTregs expressing CCR6 were similarly low (**Figure 5M**, **Supplementary Figure 4**).

We next investigated molecules associated with other specialized T helper cells. sBc- and sDC-arTregs expressed similar levels of mRNA encoding GATA3 and BCL6 as seen in primary Tregs (**Supplementary Figure 2A**). sBc- and sDC-arTregs secreted similar levels of IL-4, IL-5, IL-13 (**Figure 5F**) and IL-10 (**Figure 5N**) and contained similar percentages of IL-4- and IL-10 producing cells (**Figures 5G, O** and **Supplementary Figure 3A**). IL-4 producing cells showed a non-significant trend towards lower FOXP3 MFI compared with non-IL-4-producing cells (**Figure 5H**, **Supplementary Figure 3B**). CCR4 mRNA expression (**Supplementary Figure 2C**) was similar between sBc- and sDC-arTregs, and about 50% of sBc- and sDC-arTregs expressed CCR4 (**Figure 5I**, **Supplementary Figure 4**).

We also examined arTreg expression of tissue-homing chemokine receptors. Lymphoid-homing receptor CCR7 mRNA expression **(** **Supplementary Figure 2C** **)** and the percentage of arTregs expressing CCR7 were similar between sBc- and sDC-arTregs (**Figure 5P**). Approximately 60% of arTregs expressed CCR7. 30-40% sBc-arTregs from two responders expressed the gut-homing receptor, CCR9, but the percentages of CCR9 in the other sBc-arTreg cultures and the sDC-arTreg cultures were relatively low **(** **Figure 5Q** **)**. Levels of CCR9 mRNA were below limit of detection (**Supplementary Figure 2C**).

### arTreg Repertoire and Specificity

**Figure 6:**
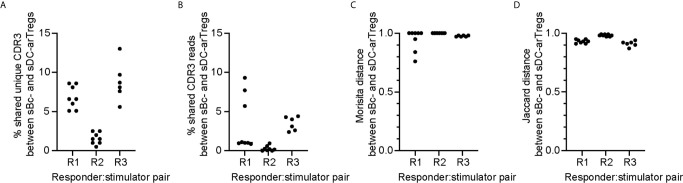
sBc- and sDC-arTregs stimulate distinct TCR repertoire likely in part due to diversity of the circulating Tregs. On d0, 2-4 replicate culture wells were set up using the same responder:stimulator combinations (R1, R2, and R3). Cytokine-matured monocyte-derived DCs were used to stimulate responder 1 and 2, and MPLA-matured monocyte-derived DCs were used to stimulate responder 3. On d11, RNA was isolated from the arTregs in each well (~500,000). RNA from ~250,000 cells was TCRβ sequenced with a read depth of 1 million. For each responder:stimulator combination, the top 100 CDR3s between sBc- and sDC-arTregs were compared. All possible sBc- and sDC-arTreg combinations within the same responder: stimulator pair were compared. Each comparison is represented by one dot. **(A)** Percentage shared unique CDR3, **(B)** percentage shared CDR3 reads, **(C)** Morisita distance comparing CDR3 usage, and **(D)** Jaccard distance comparing CDR3 usage were calculated between sBc- and sDC-arTregs.

To compare the clonal composition of the sBc- and sDC-expanded arTreg populations, we performed high-throughput sequencing of the T cell receptor β chain (TCRβ) (**Figure 6**). arTreg sets derived from three responder:stimulator combinations (responder defined as Tregs from one donor, and stimulator defined as sBc and sDC derived from the same donor allogeneic to the Treg donor) were used. For each responder:stimulator combination, 2-4 replicate culture wells were set up in parallel, thus a total of 17 TCRβ sequencing reactions were run (**Table S2**). Surprisingly, the top 100 most frequent unique TCRβ CDR3s from sBc- and sDC-arTregs generated from the same responder:stimulator pairs showed less than 10% overlap in all 3 responders (**Figure 6A**). Similarly, low sharing of total CDR3 reads among top 100 clones, counting repeated sequences, were observed (**Figure 6B**). Morisita and Jaccard distance were then used to quantify the similarity of arTreg populations produced from the same responder:stimulator pair. A distance ratio of 1 suggests no similarity, and a ratio of 0 indicates complete similarity (**Figures 6C, D**). The majority of sBc- *versus* sDC-arTregs comparisons had a ratio very close to 1, suggesting little similarity between the sBc- and sDC-arTreg TCR repertoires.

The low overlap between sBc- and sDC-arTregs’ TCR repertoires may be due to expansion of distinct arTreg clones stimulated by sBc and sDC. Alternatively, the primary Tregs at the start of the sBc and sDC cultures may have had distinct repertoire due to limited sampling (100,000 to 250,000/well) of highly clonally diverse circulating Tregs. This latter possibility is supported by the observation that replicate cultures of sBc-arTregs or sDC-arTregs had limited CDR3 overlap and repertoire similarity (**Supplementary Figure 5**). To further test this idea that the narrow sampling of a very diverse pool of Tregs at culture initiation limited repertoire overlap between sBc- and sDC-arTregs., we simulated higher Treg input, thus wider sampling, by digitally pooling replicate wells together to increase the Treg inputs to 200,000 to 750,000/condition (**Table S3**). Two of three responder:stimulator pairs (R1 and R3) had greater sharing between digitally pooled sBc- and sDC-arTreg repertoire compared to individual replicate wells. These data suggest the difference in TCR repertoires stimulated by sBc and sDC is largely due to limited sampling of a very diverse population of blood Tregs.

**Figure 7:**
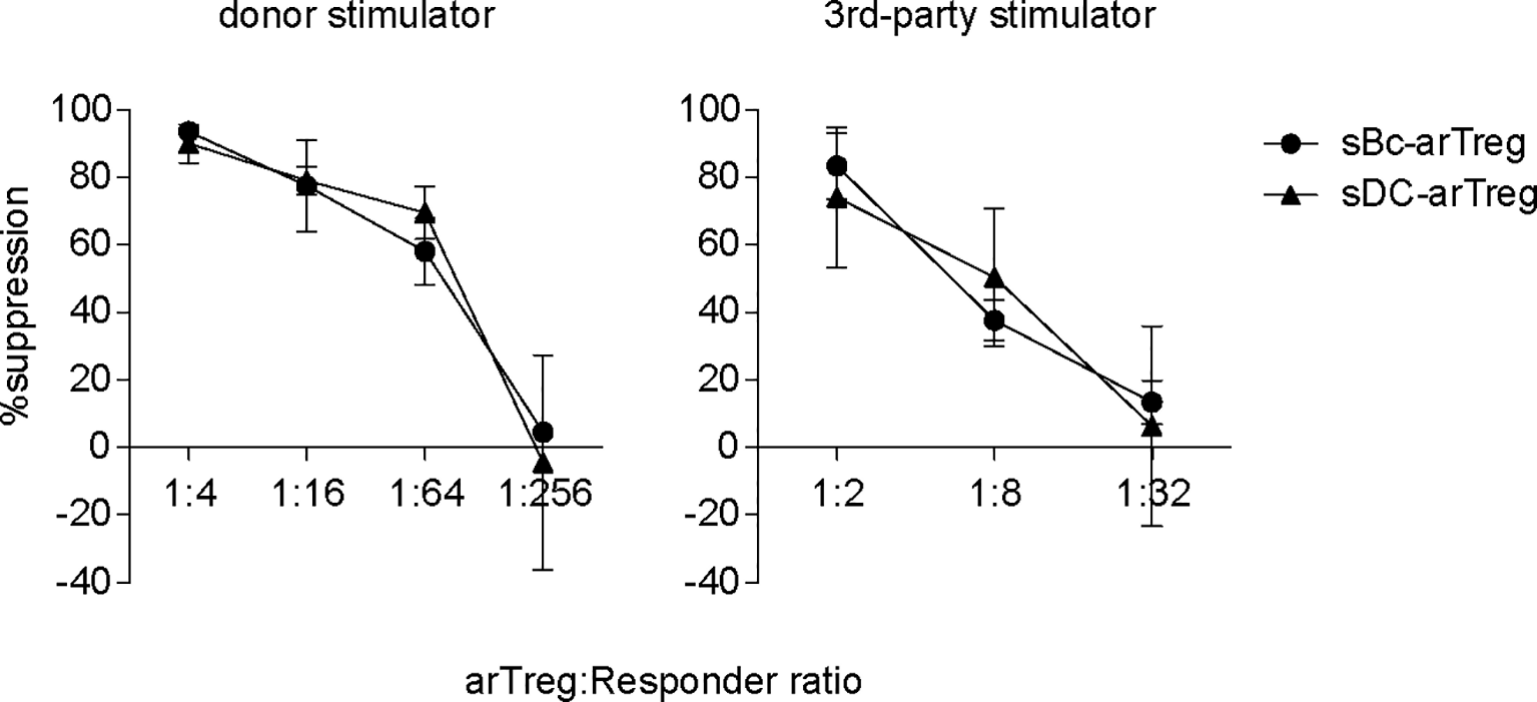
Both sBc- and sDC-arTregs suppress T cell proliferation. Suppression of autologous PBMC proliferation by sBc- and sDC-arTregs that were stimulated from APC donor (left) or from third-party donor (right). Data contain n=2 (donor) and n=3 (third-party) different responder-stimulator combinations. Statistics were performed using unpaired t-test between sBc-arTregs and sDC-arTregs at the same Treg dilution.

CDR3 sequences are useful for tracking T cells at the clonal level because they are uniquely generated during T cell development. CDR3 sequences are important determinants of peptide specificity of the TCR. However, since alloreactive TCRs likely interact with the polymorphic frame region of the HLA, not specific to the peptides presented in the HLA (28), the CDR3 sequence may not reflect the alloreactivity of the TCR. Thus, although sBc- and sDC-arTregs use different CDR3 sequences, these differences may not correlate with any differences in their alloreactivity. To compare the alloreactivity of sBc- and sDC-arTregs, we examined their suppressive function stimulated by alloantigens. sBc- and sDC-arTregs showed similar potency in suppressing the proliferation of autologous PBMCs stimulated by irradiated PBMCs from the same donor used to generate the sBcs and sDCs (**Figure 7**, left). sBc- and sDC-arTregs exhibited enhanced suppressive activity against proliferation stimulated by relevant donor APCs than by an unrelated donor (**Figure 7**, right). Overall, sBc- and sDC-arTregs appear to have similar alloreactivity and have similar potent and specific suppressive function.

## Discussion

Previous studies have shown that use of sBcs as APCs is an effective way of expanding Tregs (12, 29). sBc-arTreg manufacturing process has already been reviewed by the FDA in the context of several ongoing early phase clinical trials. Stimulatory DCs are potent APCs and may be a viable alternative for manufacturing clinical grade arTregs. In this study, we compared the stimulatory capacity of sBc and sDC to expand arTregs, and characterized the *in vitro* characteristics of these expanded arTregs.

In general, we found that sDCs, versus sBcs, led to 2-fold more arTreg expansion, which may be due to sDCs’ increased expression of CD80 and CD86 and chemokines. However, Tregs from a few responders showed comparable or less proliferation when stimulated with sDCs than with sBcs. These results are likely due to certain undefined responder-Treg characteristics, because the sDCs used in these experiments were more potent stimulators of other responder Tregs compared the sBcs (data not shown). Future *in vitro* studies using blocking antibodies against co-stimulatory molecules, chemokines, and/or other soluble factors can be performed to dissect the mechanisms on how sDC stimulate more arTreg expansion. Also, it would be interesting to determine whether cell-to-cell contact between the sDC and Tregs is necessary for increased proliferation.

We found that sBc- and sDC-arTregs are very comparable in purity, phenotype, antigen-specific suppression. Although sBc- and sDC-arTregs expressed similar levels of Treg-associated molecules, we found 23 genes that were differentially expressed by at least 2-fold between sBc- and sDC-arTregs. Further mechanistic studies can use blocking antibodies to some of these proteins to see whether they affect proliferation of Tregs or alter Treg suppression capability.

One potential concern with using sDCs is that they may produce higher levels of pro-inflammatory factors that could destabilize Tregs. We found higher IL-1β, IL-6, and IL-12p70 expression by sDC than by sBcs. However, the levels of these cytokines were very low in both cultures. More importantly, sBc- and sDC-arTregs had similar percentages of FOXP3 enhancer demethylation, similar phenotypes and suppressive functions, suggesting that neither stimulatory APC type induced Treg destabilization during the selective expansion of arTregs. Low percentages arTregs expressed IFN-γ, IL-4, and IL-17 and most of these cytokine-producing cells were FOXP3^+^, whereas most sBc- and sDC-arTregs expressed CXCR3. Together, our phenotype analyses show that both sBc- and sDC-arTregs have a stable, committed Treg phenotype and may have enhanced ability to traffic to sites of T_H_1 inflammation, such as transplanted organs undergoing alloimmune attack. Results from this study of direct comparison between sBc and sDC showed that arTregs expanded by these 2 APCs were, for the most part, are comparable in terms of purity, phenotype, and antigen-specific suppression.

Our previous study demonstrated that sBc-arTregs were effective *in vivo* in preventing alloimmune-mediated injury of human skin allografts (12). sBc-arTregs were able to home to transplanted skin allografts and were detected 6 weeks after injection into mice. In this current study, we found sBc- and sDC-arTregs to be phenotypically and functionally similar. The cells have similar high demethylation of the FOXP3 enhancer. Thus, we speculate that sDC-arTregs would have similar suppressive activity, comparable stability, and migration patterns *in vivo* as demonstrated previously with sBc-arTregs.

Currently, four registered clinical trials (NCT02188719, NCT02244801, NCT02474199, NCT02711826) are using sBcs as stimulatory APCs to generate arTregs. Using sDCs to manufacture arTregs could potentially provide some key advantages over using sBcs. First, arTregs expand 2-fold more after stimulation with sDCs than with sBcs. Second, sDC differentiation and maturation from monocytes takes 7 days, which may be further shortened (30), whereas sBc generation takes at least 10 days. Third, sDC cultures require minimal handling before harvesting, whereas sBc cultures require regular feeding and restimulation. Fourth, B cell stimulation with CD40L requires feeder cells. Although CD40L stimulation of B cells can be achieved without feeder cells by using a cell-free soluble 4-trimer CD40L reagent (UltraCD40L) (31), this reagent may not be widely available as a GMP reagent, whereas sDC production can be feeder-free and rely solely on well-defined soluble GMP-grade reagents. Fifth, B cells, not monocytes, harbor latent Epstein Barr Virus (EBV). Stimulating B cells to sBcs can potentially lead to the reactivation of latent EBV. Detection of infectious EBV in sBcs will lead to termination of clinical arTreg manufacturing. Sixth, less sDCs are required to stimulate T cells compared to sBcs. One disadvantage of using sDCs is that they do not increase in number during *in vitro* differentiation and maturation, whereas sBcs can expand more than 10-fold during 10-day culture. Despite the need for less sDCs to expand Tregs, more donor blood will be needed to manufacture sDCs. Also, spleen from the donor is commonly available to make donor sBcs. While sBcs can be generated from splenocytes, it remains to be demonstrated that splenic CD14^+^ monocytes can be differentiated into sDCs. Previous studies have shown precursors in mouse spleen can be cultured to develop into stimulatory DCs (32–34). Another potential source of monocytes from human donors is bone marrow cells. Taking in these considerations of advantages and disadvantages of using sDCs *versus* sBcs, sDCs are slightly better for their relative simpler culturing process and slightly better Treg expansion.

Together, our results show that sDCs have more potent Treg expansion ability and the resulting arTregs are similar to those expanded with sBcs. We propose that sDCs may be a viable alternative to manufacture arTregs for clinical use.

## Data Availability Statement

The raw data supporting the conclusions of this article will be made available by the authors, without undue reservation.

## Ethics Statement

The studies involving human participants were reviewed and approved by UCSF (Study 10-01980) and University of Pittsburgh (Study 19120084) institutional review board approval. The patients/participants provided their written informed consent to participate in this study.

## Author Contributions

LL: study design, data generation and analysis, and writing of the manuscript. HZ: study design, data generation and analysis, and manuscript review. KL: study design, data generation, and manuscript review. HL: data generation. AM and EM: data analysis and interpretation. FV: manuscript review. AT: study design, and manuscript review. QT: research design, data analysis, and writing of the manuscript. All authors contributed to the article and approved the submitted version.

## Funding

This work was supported by a NIAID/CTOT (Clinical Trials in Organ Transplant) grant (A130726 to QT), which is ancillary to a NIAID grant (1U01AI110658 to FV), NIAID grants (R01 AI 118777, U19 AI 131453, and U01 AI 36779 to AT, and T32 AI 74490 to HZ), Burroughs Wellcome Fund Collaborative Research Travel grant (HZ), and UC Davis Immune Monitoring Shared Resource (grant 5P30CA093373 to EM). This work was supported by the UCSF Parnassus Flow Core (RRID:SCR_018206), which is supported in part by the DRC Center Grant NIH P30 DK063720 and by the NIH S10 Instrumentation Grant S10 1S10OD018040-01, for assistance in cell sorting and generating flow cytometry data.

## Conflict of Interest

QT is a co-founder of Sonoma Biotherapeutics and a co-inventor of a patent on manufacturing arTregs. FV receives research grant support from Novartis, Genentech, Astellas and Bristol Myers Squibb.

The remaining authors declare that the research was conducted in the absence of any commercial or financial relationships that could be construed as a potential conflict of interest.

## Abbreviations

APC, antigen-presenting cell; arTconv, alloreactive conventional CD4^+^ T cell; arTreg, alloreactive regulatory T cell; CFSE, carboxyfluorescein diacetate, succinimidyl ester; cpm, counts per minute; EBV, Epstein Barr Virus; DCs, dendritic cells; DMSO, dimethyl sulfoxide; FBS, fetal bovine serum; MFI, mean fluorescence intensity; MLR, mixed leukocyte reaction; MPLA, monophosphoryl lipid A; rh, human recombinant; sBcs, CD40L-stimulated B cells; sDCs, stimulated-matured monocyte-derived DCs; Tconv, conventional CD4^+^ T cell; TCR, T cell receptor; Treg, regulatory T cell; TSDR, Treg-specific demethylated region.
