# Supplementary material for: A Comparison of Ex Vivo Expanded Human Regulatory T Cells Using Allogeneic Stimulated B Cells or Monocyte-Derived Dendritic Cells
Source: Front Immunol. 2021 Jun 18;12:679675. doi: 10.3389/fimmu.2021.679675 (PMC8253048; doi:10.3389/fimmu.2021.679675)
Supplement: Supplementary file 1 [file DataSheet_1.pdf]

Supporting Information

1. Supporting Materials and Methods

1.1 Cells

PBMCs from normal donors were isolated from either whole blood, residual cells from Trima Apheresis Collection Kits (Blood Centers of the Pacific, San Francisco, CA), or cells from leukapheresis (UCSF; Central Blood Bank of Pittsburgh) as previously described(1). Monocytes for MPLA-matured sDCs generation were isolated from either PBMCs or obtained as the elutriated monocyte fraction of normal human volunteer leukapheresis products (Institute for Transfusion Medicine, Pittsburgh, PA). Only PBMCs from whole blood or leukapheresis were used for purified T cell cultures. The cells from these donors were used in accordance with the UCSF and University of Pittsburgh IRB. Cells were cryopreserved using either CyroStor10 (Sigma Aldrich, St. Louis, MO) or 10% DMSO/90% FBS solution (DMSO-Sigma Aldrich; FBS-Omega Scientific, Tarzana, CA).

1.2 CD40L-stimulated B cells (sBcs) and stimulated matured monocyte-derived DCs (sDCs)

sBcs were generated as previously described using CD40L-expressing K562 cells(2). Briefly, PBMCs were cultured at 37ºC with irradiated (10,000 rad) CD40L-expressing K562 at a ratio of 1 B cell per 4 K562 cells in X-Vivo15 medium containing recombinant transferrin (Lonza, Morristown, NJ), human insulin (Eli Lily, Indianapolis, IN) (5 μg/mL), and 10% human AB serum (Omega Scientific). The cultures were supplemented with rhIL-4 (Miltenyi Biotec, Auburn, CA) (4 ng/mL), cyclosporine (Novartis, CA) (1 μg/mL), and ganciclovir (Genentech, CA) (20 μg/mL). After 7 days, the cells were harvested, counted, and restimulated with additional K562 cells (10 sBcs per 1 K562 cell). The culture was supplemented with rhIL-4 and ganciclovir only. After 3 days, the cells were harvested and cryopreserved. In some cases, the cells were restimulated again, and cryopreserved 3 days after the last restimulation.

Cytokine-matured sDCs were used for all experiments, except monophosphoryl lipid A (MPLA)-matured sDCs were used in responder:stimulator combination 3 in the T cell receptor sequencing analysis. Cytokine-matured sDCs were generated by isolating $CD14^+$ monocytes from PBMCs using EasySep Human Monocyte Isolation Kit or EasySep Human CD14 Positive Selection Kit II (StemCell Technologies, Vancouver, Canada). Monocytes were then differentiated and matured using ImmunoCult™ Dendritic Cell Culture Kit (StemCell Technologies) per manufacturer's instructions. The components of the differentiation and maturation supplements are propriety, but the maturation supplement minimally contains TNFα and IL-1β. MPLA-matured sDCs were generated by isolating $CD14^+$ monocytes from PBMCs using CD14 Microbeads, human (Miltenyi Biotec), or obtained as the elutriated monocyte fraction of leukapheresis products. Monocytes were then differentiated in RPMI media containing 5% human AB serum (Gemini Bioproducts, West Sacramento, CA) and supplemented with rhIL-4 (1000 IU/mL) and rhGM-CSF (1000 IU/mL) (R&D Systems, Minneapolis, MN). On d3, additional rhIL-4 and rhGM-CSF were added to the culture. On d6, the monocytes were matured by the addition of MPLA (1 μg/mL) (Invivogen, San Diego, CA) and harvested the following day. Prior to all assays, sBcs and sDCs were irradiated (25 Gray).

1.3 Mixed leukocyte reaction (MLR)

Carboxyfluorescein diacetate, succinimidyl ester (CFSE, Invitrogen/Thermo Fisher Scientific)-labeled responder PBMCs were cultured at 37ºC with irradiated allogeneic sBcs (2 sBcs per PBMC) or sDCs (1 sDC per 4 PBMC) in assay medium (RPMI, sodium pyruvate, non-essential amino acids, penicillin-streptomycin (all from Invitrogen/Thermo Fisher Scientific)), and 10% human AB serum). After 4 days, the cultures were harvested and assessed for CFSE dilution/proliferation by flow

cytometry. Precursor frequency was calculated based on Noorchashm et.al.(3). Briefly, division peaks were defined by sequential halving of CFSE MFI. This generated 5 peaks. Cells that have not divided (n=0) were defined as the peak with the highest MFI, and cells that have divided 5 or more times were defined as the peak with the lowest MFI (n=5). The number of precursors in each peak was determined by multiplying the total number cell under each peak to $1/2^n$ (where n is the division number). To calculate the precursor frequency, the sum of precursors in each peak that diluted CFSE was then divided by the total number or precursors.

1.4 T cell culture

Responder PBMCs were stained with anti-CD4, anti-CD25, and anti-CD127. Tregs ($CD4^+CD127^{lo/-}CD25^+$) and Tconvs ($CD4^+CD127^+CD25^-$) were FACS-purified using FACSAria II (BD Biosciences, San Jose, CA). T cells were cultured with irradiated allogeneic sBcs (4 sBc per 1 T cell) or sDCs (1 sDC per 4 T cell) in Optimizer T cell expansion media (Invitrogen), supplemented with 2% human AB serum, penicillin/streptomycin, and rhIL-2 (300U/mL) (Proleukin, Novartis) at 37ºC. Medium was added and/or exchanged every 2-3 days. The alloreactive T cell phenotypes were assessed on day 11.

1.5 *sBc and sDC cytokine production*

sBcs and sDCs were cultured alone at 37ºC at the same density as used in T cell expansion cultures (200,000 sBcs or 12,500 sDCs in 100 µL assay medium). After 48 h, supernatants were harvested, and cytokine and chemokine levels were measured using 65-plex human cytokine/chemokine Luminex assay (Eve Technologies, Alberta, Canada). Samples that had values below the limit of detection were given the lowest value detectable in the standard. For one sBc sample, the CCL5 value was above the highest value of the standard; this value was set to the highest value of the standard.

1.6 Flow cytometry

For sBc and sDC staining, the cell samples were incubated for 5-10 min with human TruStain FcX (BioLegend, San Diego, CA). They were then stained with antibodies at 4ºC for 30 min. After washing, the cells were analyzed on a BD Fortessa flow cytometer.

For MLR and T cell staining, the samples were labeled with fixable viability dye (eBioscience™ Fixable Viability Dye eFluor™ e506, e780 (Invitrogen) or Ghost Dye™ Violet 510 (Tonbo Biosciences, San Diego, CA)) for 20 min. The cells were then incubated with hIgG (Sigma-Aldrich) for 5 min, and then stained with antibodies against cell surface molecules for 30 min. They were then fixed and permeabilized with Foxp3 / Transcription Factor Staining Buffer Set (Invitrogen) per manufacturer's instructions. After permeabilization, the cells were stained with antibodies against transcription factors and/or cytokines for 1 h. All incubation steps were performed at 4ºC. After washing, MLR assay samples were analyzed on a Beckman Coulter Navios flow cytometer (Indianapolis, IN). T cell samples (Fig 3 and 5) were analyzed on a BD LSRII flow cytometer. Data analyses were performed using FlowJo (TreeStar, Ashland, OR) or Kaluza Analysis Software (Beckman Coulter). Precursor frequencies were calculated as previously described(3).

Information about the antibodies used are in Table S4.

1.7 Treg-specific demethylated region (TSDR) methylation assay

Frozen cell pellets containing ~100,000 cells were sent to EpigenDx (Hopkinton, MA). The samples were analyzed using the human FOXP3, Intron 1 TSDR region assay (ADS783-FS2) to obtain percentages of demethylated TSDR. All samples were from female donors. Due to X-chromosome inactivation in females, the maximum percentage of demethylation is ~50%.

1.8 Gene expression analysis of stimulated T cells

FACS-purified Tregs and Tconvs (primary-Tregs and primary-Tconvs) and cultured alloreactive T cells were stimulated with Dynabeads Human T-Activator CD3/CD28 beads (Invitrogen) for 24 h. Cells were harvested, and RNA isolated using RNeasy Micro Kit (Qiagen, Valencia, CA) with on-column DNase I digestion per manufacturer's instructions. RNA was sent to the UCSF Center for Advanced Technology for analysis using the Nanostring PanCancer Immune Profiling Panel (Seattle, WA).

Nanostring data was analyzed using the nSolver 4.0 software. The maximum counts of negative controls were 25, so any counts below 50 were considered background. For unsupervised clustering analysis, genes with counts less than the background threshold of 50 in 11 or more samples were excluded from analysis. Using this exclusion criteria, 439 genes were used for unsupervised clustering analysis and heatmap generation. For analysis of genes that were differentially expressed at least 2-fold between sBc- and sDC-arTregs, the average counts of sBc-arTregs and sDC-arTregs were compared.

1.9 Cytokine analyses of stimulated T cells

For analysis of secreted cytokines, primary T cells and cultured alloreactive T cells were stimulated with anti-CD3/CD28 beads for 24 h. Supernatants were harvested, sent to Eve Technologies, and analyzed for cytokines and chemokines by Luminex assay. In some cases, values that were above the highest value of the standard were replaced by the highest value of the standard.

For analysis of intracellular cytokines, primary T cells and cultured alloreactive T cells were stimulated with PMA (100ng/mL) and ionomycin (500ng/mL) (Sigma Aldrich) in the presence of Brefeldin A (10μg/mL) (Sigma Aldrich) and monensin (2μM) (BD Biosciences) for 5 h. Cells were then stained and analyzed by flow cytometry.

1.10 In vitro suppression assay

sBc- or sDC-arTregs were titrated in 96-well round-bottom plates in assay medium. PBMCs from the same responder from which the arTregs were generated, and irradiated PBMCs, either from the same donor as that of the sBc and sDC were generated or third-party donor, were added to each well. Each condition was performed in triplicate. The cells were cultured for 7 days, with ^{3}[H] thymidine (Perkin Elmer, Waltham, MA) added for the final 16 h of culture. Proliferation was assessed by ^{3}[H] thymidine incorporation (counts per minute - cpm). Percent suppression was calculated using the following formula = 1- [(cpm of well treated with Tregs)/(cpm of well with no Tregs)].

2. Supporting Figure Legends

Supplementary Figure 1. Different ratios of sDC:PBMC lead to similar levels of proliferation. CFSE-labeled responder PBMCs were stimulated with different ratios of sDCs (1 sDC per 4 PBMCs, 1 sDC per 8 PBMCs, or 1 sDC per 16 PBMCs) for 4 days. Percentages of T cell divided (CFSE^{lo}) were calculated. Connecting lines indicate alloreactive T cells stimulated by APCs (sBcs or sDCs) derived from the same donor.

Supplementary Figure 2. Treg specialization. Primary-Tregs and cultured arTregs were stimulated with anti-CD3/CD28 beads at 37ºC for 24 hrs. RNA was harvested from and analyzed using Nanostring's PanCancer Immune Profiling Panel. Gene expression of (A) transcription factors, (B) cytokines, and (C) chemokine receptors. Data contain 2 different responder-stimulator combinations.

Supplementary Figure 3. Cytokine production of sBc- and sDC- stimulated T cells. (A-C) Alloreactive T cells were cultured with no stimulation or stimulated with PMA/Ionomycin in the presence of Brefeldin A and monensin at 37ºC for 5 hrs. (A) Representative FACS plots of cytokine production by T cells. (B) Representative FACS plots of FOXP3 expression in cytokine- and non-cytokine producing arTregs. (C) Percentage of cytokine-producing Tconvs. Data contain 5 different responder-stimulator combinations.

Supplementary Figure 4. Representative FACS plots of chemokine receptor expression on alloreactive T cells.

Supplementary Figure 5. Circulating, blood primary Tregs are highly diverse. On d0, 2-4 replicate culture wells were set up using the same responder:stimulator combination (R1, R2, and R3). Cytokine-matured monocyte-derived DCs were used to stimulate responder 1 and 2, and MPLA-matured monocyte-derived DCs were used to stimulate responder 3. On d11, RNA was isolated from the arTregs in each well (~500,000). RNA from ~250,000 cells was TCRβ sequenced with a read depth of 1 million. For each responder:stimulator combination, the top 100 CDR3s between each replicate culture well (A-D) were compared. (A) Percentage shared unique CDR3, (B) percentage shared CDR3 reads, (C) Morisita distance comparing CDR3 usage, and (D) Jaccard distance comparing CDR3 usage were calculated. For (A-D), all possible combination within the same responder: stimulator pair were compared and represented by one dot.

3. Supporting Figures and Tables

SUPPLEMENTARY FIGURE 1

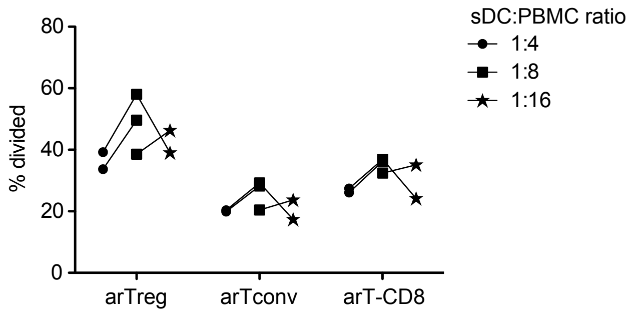

SUPPLEMENTARY FIGURE 2

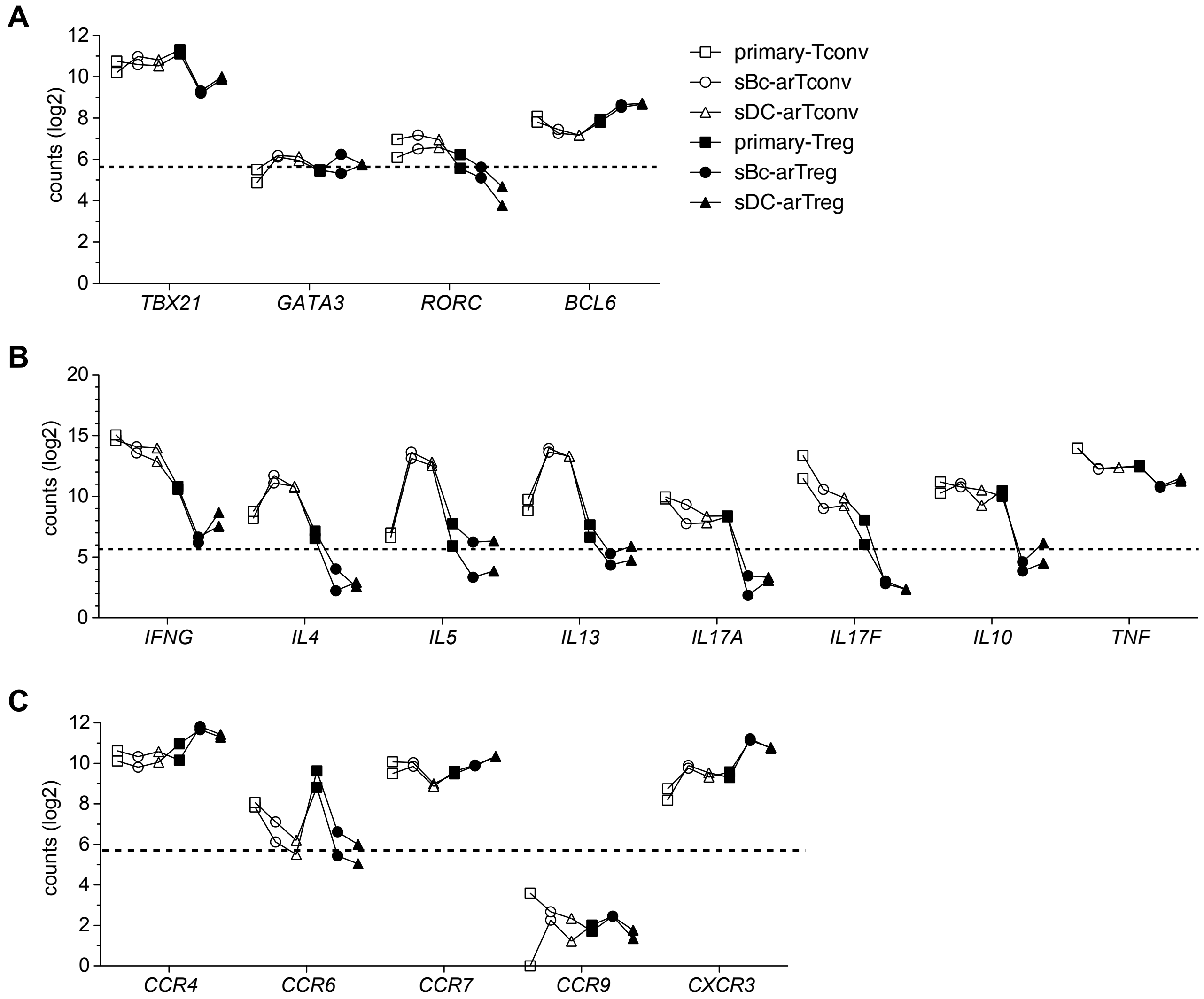

SUPPLEMENTARY FIGURE 3

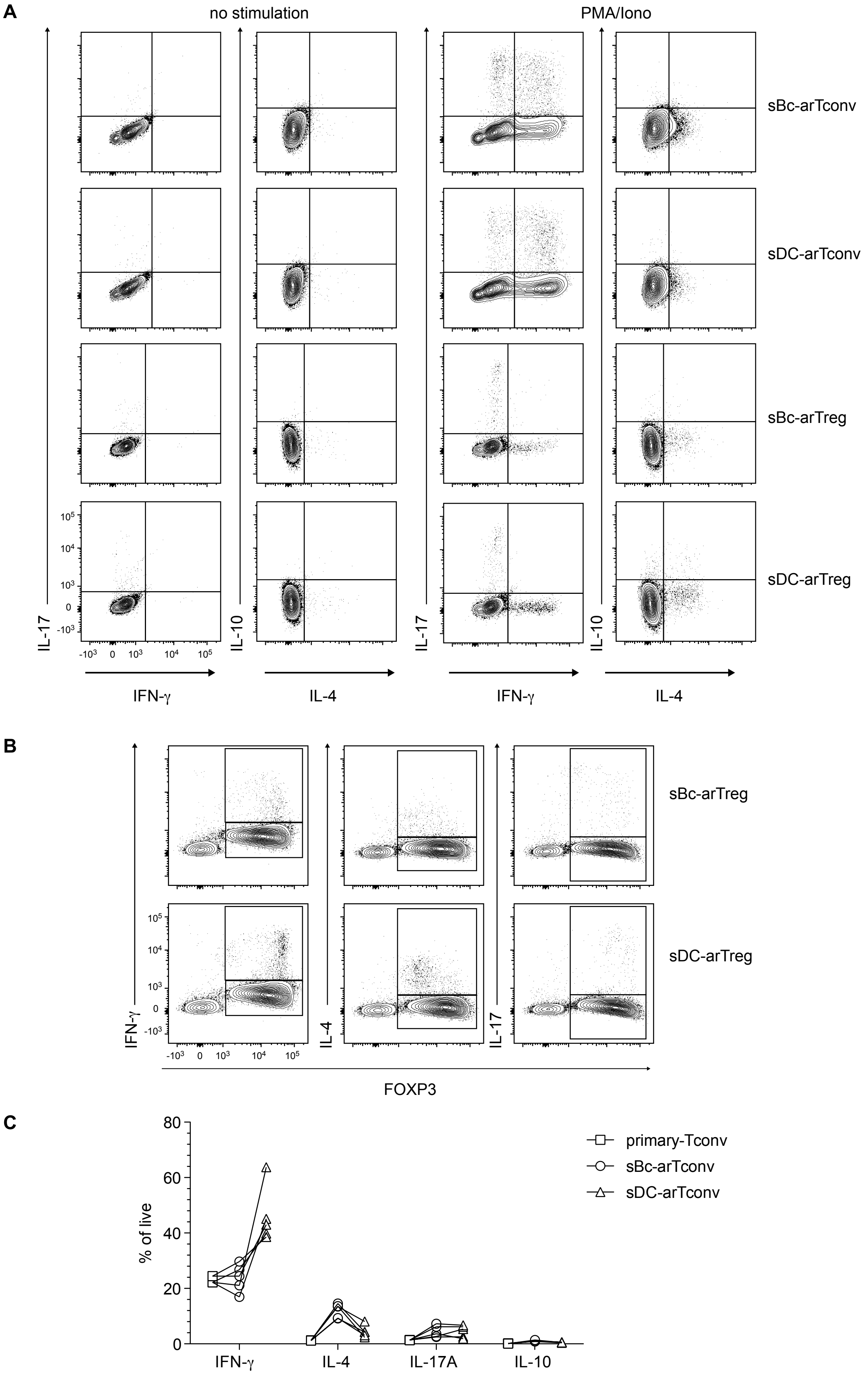

SUPPLEMENTARY FIGURE 4

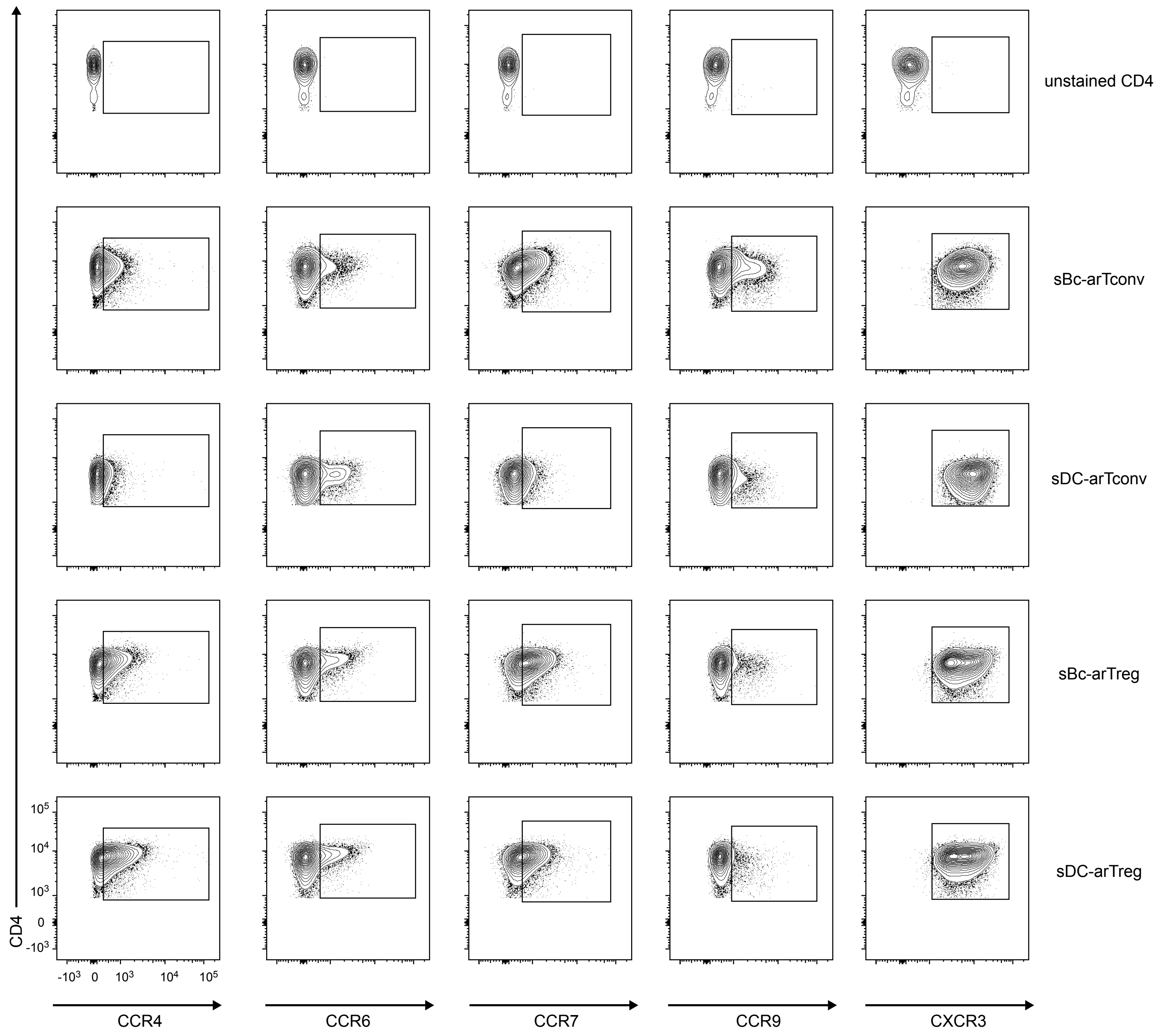

SUPPLEMENTARY FIGURE 5

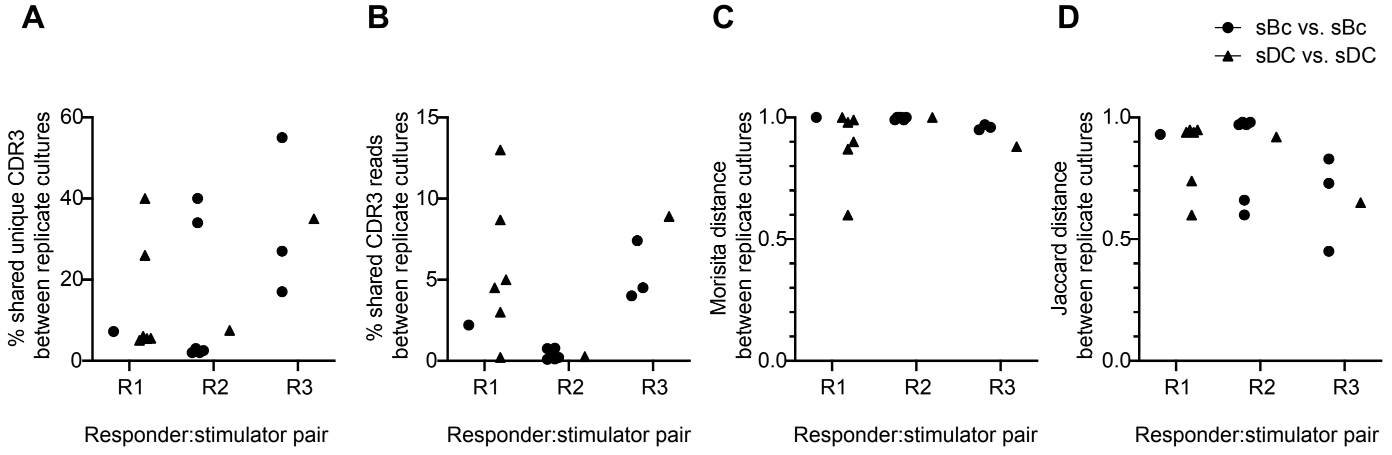

TABLE S1. Gene expression of stimulated T cells

Probe Name	Accession #	Class Name	Sample ID											
			A	B	C	D	E	F	a	b	c	d	e	f
			R1 Primary-Tconv	R1 sBc-arTconv	R1 sDC-arTconv	R1 Primary-Treg	R1 sBc-arTreg	R1 sDC-arTreg	R2 Primary-Tconv	R2 sBc-arTconv	R2 sDC-arTconv	R2 Primary-Treg	R2 sBc-arTreg	R2 sDC-arTreg
A2M	NM_000014.4	Endogenous	12.85	12.03	9.32	12.21	10.94	12.8	2.68	4.78	5.09	13.92	5.51	10.22
ABCB1	NM_000927.3	Endogenous	57.85	51.75	67.61	9.15	10.94	11.09	158.11	73.26	64.91	13.92	13.39	11.07
ABL1	NM_005157.3	Endogenous	232.3	200.97	199.32	194.29	229.8	278.17	229.12	187.94	182.01	254.72	315.01	316.72
ADA	NM_000022.2	Endogenous	222.2	651.04	507.04	134.27	285.42	141.64	170.16	582.93	414.93	100.74	227.59	194.12
ADORA2A	NM_000675.3	Endogenous	213.94	281.6	220.3	642.88	298.19	259.4	308.17	254.83	179.46	607.73	256.73	200.93
AICDA	NM_020661.1	Endogenous	11.94	12.03	9.32	3.05	14.59	9.39	25.46	20.7	16.55	9.01	12.6	12.77
AIRE	NM_000383.2	Endogenous	1	9.63	12.82	5.09	2.74	14.51	4.02	7.96	11.46	6.55	9.45	3.41
AKT3	NM_181690.1	Endogenous	552.75	440.44	434.78	391.63	420.39	381.42	631.08	406.14	427.66	334.99	390.61	374.62
ALCAM	NM_001627.3	Endogenous	465.52	381.48	306.56	194.29	443.18	717.61	333.63	329.69	272.38	219.5	366.98	626.64
AMBP	NM_001633.3	Endogenous	2.75	2.41	3.5	3.05	5.47	2.56	9.38	1.59	6.36	2.46	3.94	3.41
AMICA1	NM_153206.2	Endogenous	2608.58	2351.44	2658.77	1138.27	2037.18	1730.45	2563.18	2432.04	2940.14	900.94	1859.33	1684.93
ANP32B	NM_006401.2	Endogenous	4291.63	5177.02	5185.83	3618.26	5344.65	5593.25	4325.12	5123.69	5120.43	4345.82	5465.37	6020.29
ANXA1	NM_000700.1	Endogenous	1357.09	1207.01	1561.93	156.65	30.09	11.09	1179.09	832.98	963.5	108.93	9.45	8.51
APOE	NM_000041.2	Endogenous	6.43	7.22	5.83	4.07	13.68	10.24	10.72	9.56	11.46	4.1	13.39	6.81
APP	NM_000484.3	Endogenous	817.19	661.87	641.09	294.99	555.35	703.1	762.39	818.64	773.86	704.37	879.66	924.63
ARG1	NM_000045.2	Endogenous	7.35	15.64	17.48	15.26	35.56	11.09	13.4	7.96	11.46	5.73	15.75	9.37
ARG2	NM_001172.3	Endogenous	1	801.46	207.48	54.93	2010.74	1180.09	1.34	396.58	193.46	46.69	1986.12	1185.16
ATF1	NM_005171.2	Endogenous	673.03	539.12	503.55	524.89	568.11	460.77	637.78	506.48	542.21	547.94	546.54	487.86
ATF2	NM_001256090.1	Endogenous	835.55	983.18	810.1	1274.58	1074.22	876.32	785.17	694.41	785.31	1027.89	1056.06	968.05
ATG10	NM_001131028.1	Endogenous	98.25	142	141.04	76.29	165.97	158.71	72.35	81.23	81.46	44.23	129.15	108.13
ATG12	NM_004707.2	Endogenous	81.72	42.12	57.12	71.21	48.33	51.2	45.56	35.04	43.27	74.53	47.25	45.98
ATG16L1	NM_198890.2	Endogenous	316.78	367.04	301.89	411.97	265.36	279.02	296.11	345.61	295.29	406.24	280.36	246.06
ATG5	NM_004849.2	Endogenous	698.74	554.77	649.25	692.73	677.54	678.36	738.27	621.15	656.76	706.83	635.53	597.69
ATG7	NM_001136031.2	Endogenous	386.56	417.58	433.61	487.25	388.47	446.27	397.94	355.17	435.29	371.84	412.66	413.78
ATM	NM_000051.3	Endogenous	11.94	27.68	31.47	22.38	21.89	24.75	21.44	25.48	28	22.11	29.93	22.14
AXL	NM_021913.2	Endogenous	7.35	8.42	8.16	6.1	9.12	4.27	9.38	12.74	7.64	1.64	14.96	5.11
BAGE	NM_001187.1	Endogenous	1	13.24	10.49	5.09	9.12	10.24	21.44	20.7	14	4.91	8.66	11.07
BATF	NM_006399.3	Endogenous	1723.44	1821.95	1517.63	3314.11	2087.34	1727.04	2414.46	1850.71	1626.62	2749.51	1758.53	1689.19
BAX	NM_138761.3	Endogenous	2761	3346.65	2199.52	3295.8	2499.52	2486.46	2367.56	2893.92	2149.74	2860.08	2366.49	2350.73
BCL10	NM_003921.2	Endogenous	1137.64	971.14	860.23	1638.74	1801.91	1340.5	1200.53	1055.95	1047.51	1338.31	1827.04	1409.93
BCL2	NM_000657.2	Endogenous	498.58	949.48	531.52	733.42	888.19	802.08	539.97	743.79	481.11	611.82	893.04	735.62
BCL2L1	NM_001191.2	Endogenous	3827.94	4331.03	3158.82	4691.43	2670.04	4268.11	3978.09	3870.24	2946.51	3724.17	2317.66	3436.28
BCL6	NM_001706.2	Endogenous	271.78	154.04	144.54	246.17	401.24	422.37	223.76	175.2	145.1	225.24	370.13	410.38
BID	NM_001196.2	Endogenous	19.28	7.22	9.32	17.29	16.41	17.07	25.46	20.7	12.73	27.85	29.93	19.58
BIRC5	NM_001168.2	Endogenous	35.81	1120.36	1132.98	17.29	986.68	994.07	87.09	1149.92	1149.33	34.4	874.14	992.74
BLK	NM_001715.2	Endogenous	5.51	7.22	5.83	8.14	10.03	8.53	2.68	12.74	5.09	1	3.15	1.7
BLNK	NM_013314.2	Endogenous	5.51	7.22	8.16	8.14	9.12	8.53	4.02	12.74	3.82	10.65	6.3	7.66
BMI1	NM_005180.5	Endogenous	729.96	592.07	544.34	742.57	530.73	476.98	806.61	516.03	514.21	746.14	452.82	487
BST1	NM_004334.2	Endogenous	19.28	18.05	11.66	15.26	15.5	4.27	13.4	20.7	25.46	9.01	9.45	11.92
BST2	NM_004335.2	Endogenous	901.66	1711.23	1409.23	1677.4	1691.57	1724.48	826.7	1696.22	1332.61	1478.37	1652.21	1620.23
BTK	NM_000061.1	Endogenous	11.94	16.85	1.17	10.17	27.36	12.8	12.06	15.93	21.64	16.38	8.66	12.77
BTLA	NM_181780.2	Endogenous	752.92	255.12	227.3	188.19	874.51	312.3	535.95	329.69	213.83	349.73	1098.59	420.59
C1QA	NM_015991.2	Endogenous	7.35	4.81	10.49	6.1	5.47	5.12	16.08	11.15	11.46	4.91	4.73	5.11
C1QB	NM_000491.3	Endogenous	18.36	16.85	26.81	5.09	10.03	18.77	6.7	36.63	12.73	9.01	18.11	9.37
C1QBP	NM_001212.3	Endogenous	9493.18	7646.4	7680.25	6472.58	4626.98	6344.14	9403.25	7114.55	7473.82	8448.39	4982.62	6916.82
C1R	NM_001733.4	Endogenous	20.2	48.14	53.62	25.43	52.89	27.3	22.78	52.56	40.73	22.93	51.98	35.76
C1S	NM_001734.2	Endogenous	37.65	18.05	22.15	8.14	8.21	12.8	29.48	30.26	24.18	20.48	9.45	17.88
C2	NM_000063.3	Endogenous	4.59	10.83	2.33	2.03	9.12	7.68	9.38	19.11	29.27	5.73	14.18	6.81
C3	NM_000064.2	Endogenous	8.26	8.42	17.48	7.12	10.94	10.24	16.08	14.33	8.91	4.1	1	8.51
C3AR1	NM_004054.2	Endogenous	20.2	32.49	40.8	57.98	64.74	23.04	54.93	39.82	20.36	45.87	40.95	19.58
C4B	NM_001002029.3	Endogenous	8.26	16.85	23.31	9.15	7.3	7.68	26.8	23.89	17.82	7.37	9.45	7.66
C4BPA	NM_000715.3	Endogenous	5.51	12.03	16.32	5.09	10.03	11.95	12.06	22.3	10.18	3.28	2.36	3.41
C5	NM_001735.2	Endogenous	16.53	27.68	26.81	17.29	33.74	23.89	18.76	25.48	43.27	17.2	28.35	31.5
C6	NM_000065.2	Endogenous	6.43	8.42	4.66	10.17	8.21	7.68	9.38	9.56	7.64	4.91	5.51	6.81
C7	NM_000587.2	Endogenous	8.26	12.03	8.16	4.07	8.21	9.39	9.38	12.74	11.46	3.28	6.3	5.11
C8A	NM_000562.2	Endogenous	6.43	8.42	17.48	7.12	3.65	5.97	21.44	6.37	19.09	4.1	5.51	9.37
C8B	NM_000066.2	Endogenous	10.1	15.64	13.99	18.31	15.5	6.83	24.12	17.52	22.91	10.65	11.81	13.62
C8G	NM_000606.2	Endogenous	12.85	10.83	9.32	15.26	11.85	6.83	25.46	20.7	14	11.47	8.66	7.66
C9	NM_001737.3	Endogenous	5.51	8.42	8.16	14.24	10.94	11.95	4.02	6.37	17.82	5.73	11.81	7.66
CAMP	NM_004345.3	Endogenous	6.43	6.02	6.99	5.09	10.03	7.68	5.36	9.56	6.36	9.01	7.09	5.11
CARD11	NM_032415.2	Endogenous	701.5	969.94	734.34	761.9	790.62	624.6	680.66	837.76	730.58	787.1	703.25	682.83
CARD9	NM_052813.4	Endogenous	42.24	25.27	40.8	23.4	32.83	20.48	33.5	23.89	28	22.93	7.09	12.77
CASP1	NM_001223.3	Endogenous	250.67	458.5	195.82	305.17	627.39	273.05	200.98	358.36	168.01	330.07	528.42	279.26
CASP10	NM_032977.3	Endogenous	5.51	14.44	13.99	4.07	10.94	7.68	17.42	14.33	15.27	4.1	8.66	1.7
CASP3	NM_032991.2	Endogenous	1082.55	3871.34	2710.06	1141.32	2370.94	2067.5	1627.95	3172.64	2670.31	1209.72	2761.03	2395.86
CASP8	NM_001228.4	Endogenous	643.65	1614.96	1045.56	1827.95	830.74	583.64	791.87	1044.81	906.23	1120.45	874.14	516.8
CCL1	NM_002981.1	Endogenous	24.79	297.24	453.43	12.21	11.85	11.09	16.08	422.06	203.65	8.19	7.88	3.41
CCL11	NM_002986.2	Endogenous	11.94	13.24	10.49	8.14	9.12	9.39	14.74	7.96	7.64	8.19	8.66	8.51
CCL13	NM_005408.2	Endogenous	1.84	6.02	1.17	1.02	9.12	3.41	1.34	6.37	1.27	1.64	1.58	1
CCL14	NM_032963.3	Endogenous	6.43	9.63	1.17	5.09	3.65	8.53	8.04	25.48	5.09	4.91	6.3	5.96
CCL15	NM_032965.3	Endogenous	4.59	15.64	15.15	5.09	7.3	1	6.7	9.56	17.82	4.91	5.51	5.11
CCL16	NM_004590.2	Endogenous	5.51	7.22	13.99	3.05	9.12	12.8	2.68	11.15	2.55	3.28	6.3	6.81
CCL17	NM_002987.2	Endogenous	6.43	93.87	73.43	216.67	190.59	433.47	9.38	205.46	325.83	164.63	31.5	237.54
CCL18	NM_002988.2	Endogenous	6.43	27.68	18.65	5.09	10.94	9.39	13.4	11.15	16.55	9.01	5.51	6.81
CCL19	NM_006274.2	Endogenous	15.61	9.63	9.32	7.12	17.33	25.6	29.48	27.08	29.27	18.02	23.63	9.37
CCL2	NM_002982.3	Endogenous	5.51	18.05	13.99	7.12	10.03	5.12	9.38	23.89	15.27	20.48	4.73	3.41
CCL20	NM_004591.1	Endogenous	1169.78	160.05	169.01	1036.55	97.57	107.51	2087.53	734.23	730.58	1354.69	174.83	283.52
CCL21	NM_002989.2	Endogenous	3.67	7.22	13.99	6.1	8.21	8.53	9.38	14.33	10.18	5.73	5.51	4.26
CCL22	NM_002990.3	Endogenous	5.51	18.05	54.78	19.33	120.37	930.93	28.14	84.41	366.56	15.56	64.58	387.39
CCL23	NM_145898.1	Endogenous	2.75	3.61	1.17	1.02	9.12	5.97	1.34	1.59	6.36	1.64	1	4.26
CCL24	NM_002991.2	Endogenous	1	2.41	1.17	1.02	1	1.71	1.34	3.19	1.27	1.64	1	1
CCL25	NM_005624.2	Endogenous	9.18	8.42	17.48	6.1	14.59	5.12	16.08	19.11	16.55	6.55	13.39	11.07
CCL26	NM_006072.4	Endogenous	7.35	26.47	27.97	6.1	11.85	3.41	9.38	19.11	26.73	4.1	3.94	2.55
CCL27	NM_006664.2	Endogenous	4.59	14.44	8.16	4.07	13.68	4.27	8.04	17.52	11.46	6.55	1.58	8.51
CCL28	NM_148672.2	Endogenous	15.61	24.07	19.82	6.1	8.21	9.39	37.52	33.45	29.27	4.91	4.73	5.96
CCL3	NM_002983.2	Endogenous	10452.69	25521.69	29096.14	1670.28	238.01	255.13	19889.12	32197.85	34334.76	1036.9	173.25	284.37
CCL3L1	NM_021006.4	Endogenous	15359.5	37140.51	37535.21	2494.23	300.93	304.62	27082.91	33823.99	41280.36	1580.75	260.67	416.34
CCL4	NM_002984.2	Endogenous	16707.41	21236.39	28047.08	2051.74	203.35	185.16	24010.57	19395.8	27154.95	1194.98	111.83	289.48
CCL5	NM_002985.2	Endogenous	118.45	4096.37	1757.75	63.07	560.82	458.21	277.35	2371.52	1663.54	37.68	399.27	253.72
CCL7	NM_006273.2	Endogenous	4.59	4.81	12.82	6.1	7.3	2.56	1.34	12.74	8.91	9.01	8.66	5.11
CCL8	NM_005623.2	Endogenous	8.26	8.42	11.66	5.09	8.21	11.09	32.16	25.48	24.18	8.19	7.88	6.81
CCND3	NM_001760.2	Endogenous	1689.47	1108.33	965.13	497.42	442.27	497.46	1288.96	1076.66	896.04	1022.16	485.9	527.87
CCR1	NM_001295.2	Endogenous	15.61	123.95	45.46	4.07	6.38	6.83	42.88	95.56	19.09	6.55	7.09	10.22
CCR2	NM_001123041.2	Endogenous	27.55	409.16	107.24	15.26	40.12	11.95	119.25	307.39	61.09	6.55	54.34	11.07
CCR3	NM_001837.2	Endogenous	1.84	21.66	6.99	9.15	10.94	3.41	10.72	12.74	7.64	4.1	7.88	2.55
CCR4	NM_005508.4	Endogenous	1112.85	903.75	1068.87	2003.93	3248.19	2527.42	1582.39	1298.04	1533.71	1145.84	3626.52	2764.52
CCR5	NM_000579.1	Endogenous	15.61	116.73	39.63	20.34	27.36	6.83	53.6	87.6	17.82	13.1	22.84	9.37
CCR6	NM_031409.2	Endogenous	230.47	69.8	45.46	793.43	98.49	64	269.32	138.56	73.82	453.75	43.31	33.2
CCR7	NM_001838.2	Endogenous	1086.22	1050.57	509.37	719.18	940.17	1304.67	724.87	928.54	468.39	780.54	969.43	1293.29
CCR9	NM_031200.1	Endogenous	1	4.81	2.33	4.07	5.47	3.41	12.06	6.37	5.09	3.28	5.51	2.55
CCRL2	NM_003965.4	Endogenous	18.36	27.68	22.15	75.27	112.16	48.64	16.08	31.85	19.09	63.89	103.95	59.6
CD14	NM_000591.2	Endogenous	11.02	12.03	3.5	8.14	5.47	11.09	9.38	14.33	14	15.56	8.66	5.96
CD160	NM_007053.2	Endogenous	13.77	12.03	19.82	11.19	10.94	2.56	18.76	4.78	14	12.29	5.51	2.55
CD163	NM_004244.4	Endogenous	15.61	13.24	17.48	8.14	17.33	16.21	17.42	23.89	21.64	9.83	10.24	13.62
CD164	NM_006016.4	Endogenous	2971.27	2776.24	2830.12	3234.76	2990.12	2545.34	4059.82	2983.11	3104.33	2926.43	3255.59	2625.74
CD180	NM_005582.2	Endogenous	9.18	16.85	6.99	20.34	3.65	3.41	12.06	12.74	3.82	11.47	3.94	5.96
CD19	NM_001770.4	Endogenous	4.59	1.2	9.32	7.12	19.15	11.95	6.7	7.96	10.18	8.19	15.75	8.51
CD1A	NM_001763.2	Endogenous	4.59	6.02	3.5	7.12	4.56	3.41	2.68	7.96	2.55	6.55	1.58	3.41
CD1B	NM_001764.2	Endogenous	5.51	3.61	10.49	3.05	4.56	1.71	4.02	3.19	6.36	1.64	2.36	3.41
CD1C	NM_001765.2	Endogenous	14.69	10.83	11.66	9.15	16.41	13.65	14.74	11.15	7.64	13.92	3.94	4.26
CD1D	NM_001766.3	Endogenous	10.1	13.24	9.32	3.05	5.47	7.68	14.74	17.52	3.82	4.1	1.58	5.96
CD1E	NM_001042583.1	Endogenous	1.84	1.2	3.5	1.02	4.56	7.68	6.7	1.59	8.91	1	3.15	1
CD2	NM_001767.3	Endogenous	832.8	1062.6	699.37	1348.84	1157.2	798.67	777.13	797.94	548.57	973.84	921.39	845.45
CD200	NM_005944.5	Endogenous	1242.31	294.83	305.39	167.84	211.56	177.48	755.69	490.55	427.66	416.07	276.42	173.69
CD207	NM_015717.2	Endogenous	3.67	7.22	10.49	10.17	9.12	7.68	14.74	11.15	10.18	6.55	10.24	7.66
CD209	NM_021155.2	Endogenous	1	4.81	2.33	2.03	10.03	3.41	6.7	9.56	10.18	4.1	1.58	2.55
CD22	NM_001771.2	Endogenous	6.43	28.88	26.81	7.12	9.12	4.27	9.38	12.74	19.09	5.73	2.36	5.96
CD24	NM_013230.2	Endogenous	16.53	19.25	9.32	9.15	7.3	9.39	16.08	49.37	28	5.73	17.33	14.47
CD244	NM_016382.2	Endogenous	17.45	33.7	22.15	14.24	14.59	14.51	20.1	11.15	12.73	10.65	9.45	5.96
CD247	NM_198053.1	Endogenous	1085.3	1906.18	1271.69	1174.89	1644.16	1273.95	1393.47	1857.08	1405.16	1198.25	1892.4	1485.7
CD27	NM_001242.4	Endogenous	819.03	226.24	313.55	921.6	5010.89	2800.47	770.43	176.79	244.38	1824.82	5443.32	3580.16
CD274	NM_014143.3	Endogenous	586.72	170.88	158.52	195.31	44.68	33.28	430.1	157.68	173.1	184.28	25.2	35.76
CD276	NM_001024736.1	Endogenous	23.87	220.22	117.73	21.36	36.48	14.51	18.76	261.2	143.83	11.47	25.99	15.33
CD28	NM_001243078.1	Endogenous	1050.41	979.57	769.31	1617.38	1273.01	924.1	1015.63	638.67	794.22	1132.73	1166.31	877.8
CD33	NM_001177608.1	Endogenous	4.59	14.44	6.99	3.05	31	27.3	4.02	9.56	15.27	10.65	9.45	13.62
CD34	NM_001025109.1	Endogenous	8.26	3.61	8.16	8.14	6.38	8.53	12.06	11.15	11.46	7.37	5.51	4.26
CD36	NM_001001548.2	Endogenous	3.67	6.02	2.33	1.02	3.65	8.53	8.04	4.78	7.64	1.64	6.3	5.11
CD37	NM_001774.2	Endogenous	62.44	115.53	108.4	94.6	176.91	110.93	73.69	103.52	98	87.64	135.45	111.53
CD38	NM_001775.2	Endogenous	16.53	330.93	137.54	22.38	93.93	11.95	17.42	453.92	147.64	54.88	133.88	22.14
CD3D	NM_000732.4	Endogenous	7979.08	6740.24	5852.56	12553.53	8107.7	5862.04	7461.77	5835.62	5308.8	10141.34	8590.23	6413.64
CD3E	NM_000733.2	Endogenous	2282.62	2911.02	2212.34	2887.89	3405.03	2530.83	1881.19	2158.1	2019.92	2375.21	3109.9	2796.02
CD3EAP	NM_012099.1	Endogenous	262.6	167.27	184.17	262.44	143.17	184.31	325.59	210.24	202.37	330.07	158.29	182.2
CD3G	NM_000073.2	Endogenous	795.15	983.18	888.2	704.93	1230.15	866.93	671.28	941.28	880.77	764.16	1383.67	1067.66
CD4	NM_000616.4	Endogenous	1176.2	4635.49	2771.84	1729.28	2767.62	2307.27	1274.22	3725.31	2946.51	1277.7	2097.95	1775.18
CD40	NM_001250.4	Endogenous	77.13	48.14	40.8	51.88	41.95	38.4	46.9	38.22	43.27	90.91	48.04	52.79
CD40LG	NM_000074.2	Endogenous	7530.09	4226.34	3340.66	2136.17	265.36	241.48	5878.04	3959.43	2756.86	1755.2	198.45	194.12
CD44	NM_001001392.1	Endogenous	4223.68	3695.64	2776.5	6339.33	5216.07	4228.86	6801.21	3965.8	3291.43	5223.83	4213.22	3543.55

**TABLE S1. Gene expression of stimulated T cells (cont.)**														
CD46	NM_172350.1	Endogenous	1586.63	1912.2	1771.74	1887.96	1864.84	1706.56	1536.84	1823.63	1718.27	1747.83	1819.95	1769.22
CD47	NM_001777.3	Endogenous	4773.68	4131.27	4116.96	6115.54	3116.87	3224.55	4776.66	4005.62	4041.1	5436.78	3171.33	3243.01
CD48	NM_001778.2	Endogenous	2615.93	2335.8	2161.05	1781.16	1432.6	1870.39	2516.29	2156.5	1813.72	2367.02	1489.98	1692.6
CD5	NM_014207.2	Endogenous	3125.52	2089.1	1709.96	2128.03	2784.94	2361.88	2999.98	2363.55	2277.02	2668.43	2836.64	2768.77
CD53	NM_001040033.1	Endogenous	2962.08	4874.97	3655.38	3316.14	4171.03	2804.73	3580.15	3867.06	3287.61	3133.64	4076.19	2916.07
CD55	NM_000574.3	Endogenous	62.44	44.53	43.13	31.53	28.27	42.66	53.6	65.3	45.82	31.94	42.53	26.39
CD58	NM_001779.2	Endogenous	720.78	1333.37	1050.22	1511.59	1872.13	2000.94	829.38	1317.16	1150.6	936.98	1556.13	1657.69
CD59	NM_000611.4	Endogenous	820.86	1754.56	1077.03	1097.58	2404.68	2393.45	1022.33	1605.43	1187.51	959.91	2157.01	2074.88
CD6	NM_006725.3	Endogenous	2006.25	611.33	631.76	478.09	311.87	214.17	1929.42	922.17	812.04	704.37	405.57	305.65
CD63	NM_001780.4	Endogenous	2371.69	3089.13	2675.09	2878.74	1997.97	1736.43	2934.33	2561.05	2913.41	2399.78	1569.52	1533.38
CD68	NM_001251.2	Endogenous	40.4	126.36	111.9	153.6	172.35	135.67	40.2	47.78	73.82	89.28	94.5	71.52
CD7	NM_006137.6	Endogenous	1667.44	605.31	349.69	2759.72	2377.32	1119.5	2094.23	676.89	337.29	2773.27	2382.24	1589.58
CD70	NM_001252.2	Endogenous	44.99	837.57	702.87	348.91	617.36	965.06	42.88	887.13	865.5	134.32	459.12	817.35
CD74	NM_001025159.1	Endogenous	774.95	4645.12	4951.54	2360.97	7783.98	6103.52	848.14	3796.98	4756.41	2438.28	6083.57	5686.54
CD79A	NM_001783.3	Endogenous	12.85	12.03	3.5	10.17	156.85	44.37	8.04	12.74	7.64	16.38	141.75	67.26
CD79B	NM_021602.2	Endogenous	367.28	315.29	208.65	483.18	454.13	407.87	247.88	207.05	204.92	307.96	307.13	301.4
CD80	NM_005191.3	Endogenous	14.69	703.99	418.46	224.81	670.25	887.41	24.12	673.71	574.03	141.69	355.96	542.35
CD81	NM_004356.3	Endogenous	4913.24	6208.34	5523.86	4175.7	3474.34	3908.02	5552.45	5668.39	5275.71	4265.56	3802.92	4101.22
CD83	NM_004233.3	Endogenous	509.6	381.48	496.55	482.16	2569.73	3110.21	846.8	492.14	520.57	649.5	2546.04	3334.11
CD84	NM_001184879.1	Endogenous	285.56	252.71	139.87	171.91	123.11	56.32	111.21	140.16	82.73	82.72	95.29	45.12
CD86	NM_175862.3	Endogenous	1.84	134.78	180.67	4.07	5.47	26.45	8.04	157.68	178.19	4.91	5.51	16.18
CD8A	NM_001768.5	Endogenous	18.36	37.31	41.96	22.38	15.5	17.07	16.08	1.59	2.55	9.01	7.09	8.51
CD8B	NM_004931.3	Endogenous	9.18	16.85	3.5	3.05	21.89	12.8	6.7	4.78	19.09	14.74	18.9	14.47
CD9	NM_001769.2	Endogenous	43.15	1063.8	600.29	20.34	209.74	90.45	60.29	705.56	465.84	15.56	111.04	42.57
CD96	NM_005816.4	Endogenous	328.71	521.07	431.28	266.51	1456.3	533.3	514.51	482.59	332.2	281.75	1265.54	568.74
CD97	NM_078481.2	Endogenous	3044.72	2055.41	1570.09	1435.3	1303.11	1114.38	2865.99	2045.02	1652.08	1200.71	992.27	1115.34
CD99	NM_002414.3	Endogenous	3436.79	4855.72	3667.03	2578.66	2364.56	2316.66	2935.67	4496.17	4232.02	1827.28	2202.68	2232.39
CDH1	NM_004360.2	Endogenous	22.95	13.24	18.65	28.48	78.42	47.78	33.5	33.45	15.27	42.59	173.25	98.76
CDH5	NM_001795.3	Endogenous	13.77	27.68	33.8	17.29	29.18	18.77	30.82	27.08	35.64	18.84	24.41	17.88
CDK1	NM_001786.4	Endogenous	69.78	3347.86	3205.45	23.4	2193.12	2714.29	115.23	2827.03	3259.61	62.25	2131.02	2820.71
CDKN1A	NM_000389.2	Endogenous	226.79	658.26	386.98	1045.7	358.38	396.78	213.04	578.15	370.38	631.48	295.32	321.83
CEACAM1	NM_001712.3	Endogenous	24.79	74.61	38.47	31.53	41.95	26.45	49.58	58.93	24.18	24.57	42.53	13.62
CEACAM6	NM_002483.4	Endogenous	9.18	6.02	2.33	6.1	6.38	8.53	9.38	7.96	7.64	4.1	3.94	8.51
CEACAM8	NM_001816.3	Endogenous	5.51	4.81	2.33	8.14	1	5.12	1.34	3.19	1.27	4.91	4.73	5.11
CEBPB	NM_005194.2	Endogenous	628.96	1077.04	1633.03	495.39	320.99	380.56	1216.61	1452.53	1681.35	429.18	323.67	320.13
CFB	NM_001710.5	Endogenous	6.43	10.83	10.49	4.07	9.12	5.12	4.02	4.78	6.36	3.28	6.3	6.81
CFD	NM_001928.2	Endogenous	2.75	21.66	4.66	6.1	15.5	14.51	22.78	17.52	22.91	13.1	18.11	24.69
CFI	NM_000204.3	Endogenous	14.69	131.17	45.46	3.05	7.3	4.27	13.4	184.75	34.37	3.28	3.15	3.41
CFP	NM_002621.2	Endogenous	19.28	19.25	16.32	14.24	26.45	11.09	16.08	14.33	10.18	17.2	32.29	17.03
CHIT1	NM_003465.2	Endogenous	5.51	8.42	11.66	13.22	15.5	8.53	17.42	9.56	10.18	2.46	15.75	15.33
CHUK	NM_001278.3	Endogenous	852.08	660.67	688.88	996.88	608.24	773.93	777.13	590.89	660.58	892.75	585.91	809.69
CKLF	NM_181640.2	Endogenous	1359.84	2660.72	2060.81	1560.42	2169.41	1675.84	1193.83	2322.14	2018.64	1343.22	2244.42	1830.52
CLEC4A	NM_194448.2	Endogenous	34.89	34.9	24.48	51.88	28.27	25.6	22.78	35.04	21.64	26.21	24.41	28.1
CLEC4C	NM_203503.1	Endogenous	10.1	16.85	8.16	18.31	10.94	12.8	9.38	12.74	10.18	9.01	5.51	5.96
CLEC5A	NM_013252.2	Endogenous	1.84	14.44	12.82	6.1	6.38	8.53	8.04	11.15	10.18	1.64	3.94	8.51
CLEC6A	NM_001007033.1	Endogenous	5.51	3.61	1.17	1.02	1	1	16.08	4.78	5.09	1.64	3.15	1
CLEC7A	NM_197954.2	Endogenous	5.51	3.61	5.83	5.09	7.3	6.83	4.02	6.37	6.36	12.29	3.94	8.51
CLU	NM_001831.2	Endogenous	12.85	7.22	11.66	8.14	7.3	3.41	13.4	3.19	11.46	10.65	6.3	6.81
CMA1	NM_001836.2	Endogenous	41.32	25.27	47.79	9.15	9.12	8.53	29.48	14.33	14	7.37	8.66	5.11
CMKLR1	NM_004072.1	Endogenous	12.85	10.83	10.49	8.14	7.3	10.24	13.4	22.3	16.55	4.1	7.09	9.37
COL3A1	NM_000090.3	Endogenous	9.18	36.1	17.48	15.26	14.59	14.51	14.74	41.41	17.82	16.38	10.24	10.22
COLEC12	NM_130386.2	Endogenous	1.84	7.22	3.5	2.03	11.85	1.71	4.02	3.19	7.64	7.37	3.15	9.37
CR1	NM_000651.4	Endogenous	7.35	8.42	26.81	3.05	10.03	8.53	18.76	3.19	15.27	9.01	3.94	4.26
CR2	NM_001006658.1	Endogenous	10.1	10.83	6.99	13.22	8.21	25.6	6.7	12.74	12.73	14.74	10.24	21.29
CREB1	NM_004379.3	Endogenous	571.11	773.79	655.08	580.83	927.4	864.37	593.56	834.57	701.31	553.67	944.23	882.06
CREB5	NM_182898.2	Endogenous	7.35	7.22	4.66	6.1	7.3	5.12	6.7	12.74	11.46	4.1	4.73	5.11
CREBBP	NM_004380.2	Endogenous	27.55	20.46	20.98	25.43	26.45	13.65	18.76	25.48	16.55	18.02	26.78	21.29
CRP	NM_000567.2	Endogenous	1	4.81	1.17	3.05	1	8.53	5.36	1.59	7.64	3.28	2.36	7.66
CSF1	NM_000757.4	Endogenous	426.96	1948.3	1269.36	1442.42	486.95	436.88	840.1	1935.12	1224.42	1349.78	392.18	362.7
CSF1R	NM_005211.2	Endogenous	7.35	8.42	8.16	3.05	6.38	1.71	5.36	14.33	6.36	4.1	3.15	5.11
CSF2	NM_000758.2	Endogenous	3111.75	13113.44	12969.82	100.7	18.24	17.07	5776.2	11319.26	10085.58	77.81	14.18	15.33
CSF2RB	NM_000395.2	Endogenous	11.94	244.29	57.12	329.58	390.29	209.05	18.76	254.83	64.91	243.25	315.01	175.39
CSF3	NM_000759.3	Endogenous	8.26	7.22	11.66	13.22	13.68	11.95	14.74	12.74	10.18	6.55	3.94	5.96
CSF3R	NM_156038.2	Endogenous	15.61	6.02	13.99	12.21	10.03	6.83	14.74	17.52	15.27	7.37	7.88	8.51
CT45A1	NM_001017417.1	Endogenous	32.14	10.83	27.97	30.52	10.94	4.27	29.48	25.48	25.46	18.02	9.45	5.11
CTAG1B	NM_001327.2	Endogenous	8.26	16.85	19.82	11.19	20.97	17.92	18.76	19.11	19.09	7.37	10.24	14.47
CTAGE1	NM_172241.2	Endogenous	6.43	12.03	4.66	1.02	10.03	5.12	8.04	14.33	11.46	9.01	7.09	8.51
CTCFL	NM_001269042.1	Endogenous	7.35	3.61	3.5	6.1	9.12	9.39	13.4	11.15	11.46	5.73	3.94	1
CTLA4	NM_005214.3	Endogenous	13052.09	15703.16	11276.18	96091.85	27215.66	24205.86	22272.75	13117.41	13253.55	68471.67	22010.31	21217.9
CTSG	NM_001911.2	Endogenous	9.18	25.27	29.14	6.1	8.21	1.71	37.52	38.22	30.55	7.37	5.51	3.41
CTSH	NM_004390.3	Endogenous	289.23	1695.59	1556.1	161.74	410.36	366.06	371.15	1423.87	1146.78	76.17	285.08	277.56
CTSL	NM_001912.4	Endogenous	189.15	44.53	134.05	166.82	122.19	52.05	140.69	39.82	96.73	86.82	60.64	40.87
CTSS	NM_004079.3	Endogenous	842.9	669.09	523.36	2042.58	1540.2	979.57	530.59	549.48	347.47	1368.61	1430.92	951.02
CTSW	NM_001335.3	Endogenous	16.53	154.04	76.93	16.28	49.24	29.01	20.1	50.97	40.73	16.38	29.93	30.65
CX3CL1	NM_002996.3	Endogenous	19.28	1.2	6.99	13.22	13.68	5.12	24.12	22.3	20.36	10.65	4.73	11.07
CX3CR1	NM_001337.3	Endogenous	10.1	20.46	19.82	100.7	121.28	87.03	24.12	14.33	16.55	38.49	40.95	48.53
CXCL1	NM_001511.1	Endogenous	19.28	20.46	25.64	8.14	11.85	15.36	75.03	50.97	35.64	13.92	11.03	14.47
CXCL10	NM_001565.1	Endogenous	11.02	42.12	68.77	15.26	11.85	5.97	26.8	184.75	40.73	35.22	13.39	17.88
CXCL11	NM_005409.4	Endogenous	6.43	10.83	6.99	4.07	3.65	5.12	8.04	9.56	10.18	4.1	4.73	3.41
CXCL12	NM_000609.5	Endogenous	2.75	1.2	1.17	2.03	6.38	2.56	1.34	3.19	1.27	7.37	1	1
CXCL13	NM_006419.2	Endogenous	175.37	71	38.47	13.22	22.8	29.86	123.27	195.9	54.73	5.73	6.3	17.03
CXCL14	NM_004887.4	Endogenous	2.75	10.83	5.83	7.12	10.94	8.53	8.04	11.15	6.36	6.55	5.51	1.7
CXCL16	NM_001100812.1	Endogenous	112.02	190.14	183	132.24	123.11	138.23	156.77	176.79	198.56	89.28	131.52	130.27
CXCL2	NM_002089.3	Endogenous	11.02	97.48	59.45	13.22	10.03	9.39	17.42	52.56	14	18.84	3.15	10.22
CXCL3	NM_002090.2	Endogenous	11.02	18.05	16.32	8.14	11.85	7.68	6.7	14.33	10.18	7.37	3.15	4.26
CXCL5	NM_002994.3	Endogenous	7.35	7.22	8.16	13.22	8.21	5.97	5.36	12.74	10.18	8.19	8.66	8.51
CXCL6	NM_002993.3	Endogenous	5.51	4.81	5.83	1.02	9.12	4.27	6.7	9.56	3.82	4.1	7.88	5.96
CXCL9	NM_002416.1	Endogenous	44.99	50.54	69.94	46.79	58.36	37.54	48.24	90.78	84	63.07	59.06	48.53
CXCR1	NM_000634.2	Endogenous	23.87	27.68	26.81	25.43	41.04	23.89	21.44	46.19	44.55	31.12	31.5	26.39
CXCR2	NM_001557.2	Endogenous	2.75	7.22	9.32	4.07	8.21	8.53	6.7	9.56	5.09	7.37	3.15	5.96
CXCR3	NM_001504.1	Endogenous	292.9	951.89	743.66	632.71	2373.68	1722.77	431.44	871.2	642.76	763.34	2244.42	1773.48
CXCR4	NM_003467.2	Endogenous	2046.65	6713.76	3018.95	1931.7	2260.6	1735.57	3022.76	5042.46	3100.51	1239.21	2111.33	1899.49
CXCR5	NM_001716.3	Endogenous	313.1	83.03	81.59	386.54	113.08	53.76	308.17	105.12	159.1	319.43	54.34	44.27
CXCR6	NM_006564.1	Endogenous	46.83	571.61	208.65	99.69	294.54	111.78	218.4	353.58	176.92	68.8	335.48	146.44
CYBB	NM_000397.3	Endogenous	3.67	4.81	6.99	5.09	5.47	5.12	4.02	11.15	2.55	4.91	5.51	3.41
CYFIP2	NM_001037332.2	Endogenous	2017.27	2456.14	2274.12	2066.99	2355.44	2462.57	2149.16	2295.07	2089.92	2021.39	2497.22	2796.02
CYLD	NM_015247.1	Endogenous	2413.01	2819.56	2213.51	1983.58	2566.09	1845.65	3075.01	3282.54	2586.31	1665.11	2357.83	1829.67
DDX43	NM_018665.2	Endogenous	20.2	22.86	20.98	22.38	10.03	16.21	30.82	23.89	33.09	30.3	9.45	12.77
DDX58	NM_014314.3	Endogenous	63.36	156.44	67.61	165.81	134.05	70.82	76.37	232.53	64.91	139.24	172.47	122.6
DEFB1	NM_005218.3	Endogenous	11.02	13.24	24.48	7.12	25.53	8.53	18.76	15.93	17.82	9.01	12.6	11.92
DMBT1	NM_007329.2	Endogenous	15.61	10.83	9.32	6.1	5.47	10.24	21.44	11.15	7.64	9.83	5.51	1
DOCK9	NM_001130048.1	Endogenous	568.36	380.27	477.9	499.46	459.6	482.96	472.98	293.06	378.02	506.17	487.47	503.18
DPP4	NM_001935.3	Endogenous	137.73	953.09	857.89	46.79	200.62	221	280.03	842.53	566.39	76.17	229.95	332.05
DUSP4	NM_057158.2	Endogenous	2508.5	3164.94	2190.19	6135.88	12463.85	9379.26	2699.85	2518.05	2330.48	5215.64	13644.52	11506.76
DUSP6	NM_001946.2	Endogenous	1371.78	2891.77	1770.57	477.08	171.44	209.91	1997.75	2524.42	1741.18	357.1	156.72	190.72
EBI3	NM_005755.2	Endogenous	16.53	200.97	134.05	59	143.17	261.1	20.1	203.86	117.1	46.69	94.5	147.29
ECSIT	NM_001142464.2	Endogenous	400.33	299.65	299.56	370.27	270.83	292.68	396.6	261.2	355.11	362.02	289.81	285.22
EGR1	NM_001964.2	Endogenous	94.57	162.46	113.06	136.31	149.55	56.32	266.64	100.34	112.01	106.48	148.05	65.56
EGR2	NM_000399.3	Endogenous	604.17	394.71	510.54	424.18	480.57	307.18	850.82	466.66	476.02	278.47	426.83	317.57
ELANE	NM_001972.2	Endogenous	17.45	15.64	19.82	21.36	22.8	7.68	21.44	19.11	10.18	3.28	11.81	12.77
ELK1	NM_005229.3	Endogenous	525.21	675.11	560.66	437.41	422.21	407.87	460.92	485.77	521.84	457.02	416.6	446.14
ENG	NM_001114753.1	Endogenous	101	129.97	149.2	64.08	43.77	55.46	85.75	117.86	136.19	72.89	44.1	57.04
ENTPD1	NM_001098175.1	Endogenous	10.1	140.8	88.59	387.56	447.74	280.73	9.38	9.56	8.91	28.67	19.69	18.73
EOMES	NM_005442.2	Endogenous	11.94	31.29	19.82	9.15	9.12	7.68	12.06	17.52	14	9.83	7.88	3.41
EP300	NM_001429.2	Endogenous	641.82	805.07	673.73	875.83	917.37	673.24	840.1	775.64	733.13	733.04	827.68	716.03
EPCAM	NM_002354.1	Endogenous	8.26	14.44	19.82	7.12	16.41	13.65	36.18	38.22	22.91	3.28	18.11	10.22
ETS1	NM_005238.3	Endogenous	5510.07	9358.84	6982.05	7513.2	8642.08	5658.1	6806.57	7195.78	5951.56	6038.78	9419.49	6567.75
EWSR1	NM_013986.3	Endogenous	1696.82	2036.15	1861.49	1925.6	1702.52	1835.41	1745.86	1898.49	1798.45	2024.66	1723.09	1890.97
F12	NM_000505.3	Endogenous	19.28	32.49	36.13	32.55	43.77	66.56	14.74	22.3	35.64	33.58	31.5	40.02
F13A1	NM_000129.3	Endogenous	7.35	4.81	6.99	1.02	7.3	5.97	14.74	20.7	8.91	1.64	4.73	1
F2RL1	NM_005242.3	Endogenous	43.15	9.63	12.82	27.46	10.94	8.53	49.58	22.3	10.18	47.5	18.11	22.99
FADD	NM_003824.2	Endogenous	83.56	78.22	78.1	94.6	95.75	80.21	80.39	74.86	76.37	59.79	102.38	92.8
FAS	NM_000043.3	Endogenous	41.32	80.63	69.94	141.39	103.96	88.74	49.58	89.19	24.18	92.55	85.84	70.67
FCER1A	NM_002001.2	Endogenous	10.1	13.24	24.48	7.12	7.3	9.39	22.78	15.93	16.55	10.65	5.51	7.66
FCER1G	NM_004106.1	Endogenous	11.02	13.24	19.82	17.29	116.72	68.26	13.4	12.74	15.27	14.74	34.65	26.39
FCER2	NM_002002.4	Endogenous	72.54	194.95	301.89	179.03	238.92	188.57	16.08	47.78	33.09	14.74	18.9	22.99
FCGR1A	NM_000566.3	Endogenous	14.69	14.44	19.82	15.26	17.33	15.36	21.44	25.48	21.64	13.1	9.45	17.03
FCGR2A	NM_021642.3	Endogenous	16.53	21.66	20.98	11.19	23.71	6.83	37.52	12.74	19.09	11.47	7.88	11.92
FCGR2B	NM_001002273.1	Endogenous	1	4.81	4.66	3.05	7.3	4.27	5.36	12.74	8.91	3.28	3.94	5.11
FCGR3A	NM_000569.6	Endogenous	6.43	12.03	10.49	4.07	7.3	10.24	16.08	6.37	14	4.1	8.66	4.26
FEZ1	NM_005103.4	Endogenous	38.56	30.08	11.66	20.34	22.8	57.17	56.27	19.11	19.09	22.11	25.2	42.57
FLT3	NM_004119.1	Endogenous	8.26	13.24	9.32	3.05	8.21	9.39	13.4	9.56	17.82	9.01	3.94	8.51
FLT3LG	NM_001459.3	Endogenous	426.96	476.55	414.96	1324.42	718.58	423.23	566.77	387.02	350.02	1011.51	564.65	345.67
FN1	NM_212482.1	Endogenous	7.35	21.66	19.82	5.09	20.06	68.26	16.08	27.08	6.36	2.46	17.33	41.72
FOS	NM_005252.2	Endogenous	123.96	115.53	173.68	451.65	62.01	33.28	574.81	113.08	78.91	270.28	59.06	62.15
FOXJ1	NM_001454.3	Endogenous	16.53	10.83	12.82	13.22	7.3	8.53	16.08	17.52	11.46	17.2	4.73	12.77
FOXP3	NM_014009.3	Endogenous	44.07	628.17	427.78	3554.17	3987.74	3384.96	131.31	444.36	553.66	3254.04	3442.24	3070.17
FPR2	NM_001462.3	Endogenous	11.94	80.63	52.45	5.09	12.77	17.92	13.4	93.97	56	10.65	5.51	5.11
FUT5	NM_002034.2	Endogenous	44.07	64.98	83.92	37.64	52.89	56.32	45.56	84.41	56	46.69	40.95	56.19
FUT7	NM_004479.3	Endogenous	59.68	494.6	535.02	222.77	322.81	250.86	29.48	423.66	632.58	115.48	158.29	211.15
FYN	NM_002037.3	Endogenous	4962.82	2142.05	1706.46	4223.5	5436.75	4672.56	3692.7	1527.39	1518.44	3783.96	4154.15	4365.16

TABLE S1. Gene expression of stimulated T cells (cont.)														
GAGE1	NM_001040663.2	Endogenous	8.26	12.03	11.66	8.14	7.3	5.97	5.36	14.33	16.55	9.83	6.3	10.22
GATA3	NM_001002295.1	Endogenous	29.38	69.8	61.78	43.74	75.69	54.61	45.56	73.26	70	45.05	40.16	53.64
GNLY	NM_006433.2	Endogenous	23.87	2558.43	794.95	21.36	65.66	17.07	99.15	692.82	257.1	16.38	23.63	11.07
GPI	NM_000175.2	Endogenous	2374.44	6177.05	4473.64	2270.44	4241.25	4856.02	2359.52	4080.48	4074.2	2504.62	4254.95	4675.92
GTF3C1	NM_001520.3	Endogenous	516.02	605.31	649.25	518.78	623.74	579.38	604.28	622.74	659.3	484.05	566.22	528.72
GZMA	NM_006144.2	Endogenous	37.65	2324.97	1220.4	16.28	31	8.53	352.39	997.02	440.38	13.1	10.24	5.96
GZMB	NM_004131.3	Endogenous	22587.5	55687.3	48800.9	1331.54	121.28	42.66	44714.34	39818.88	35459.9	1149.11	23.63	24.69
GZMH	NM_033423.3	Endogenous	253.42	811.09	755.32	24.41	20.97	7.68	217.06	304.2	451.84	26.21	7.88	6.81
GZMK	NM_002104.2	Endogenous	14.69	150.42	33.8	5.09	6.38	1	16.08	7.96	1.27	5.73	1	1
GZMM	NM_005317.2	Endogenous	144.16	234.66	117.73	126.14	110.34	81.91	144.71	181.57	137.46	156.44	133.88	110.68
HAMP	NM_021175.2	Endogenous	15.61	22.86	26.81	11.19	10.94	7.68	14.74	17.52	17.82	4.91	2.36	14.47
HAVCR2	NM_032782.3	Endogenous	179.05	1819.54	927.83	244.13	1356	792.7	511.83	1302.82	674.58	182.65	1045.82	689.64
HCK	NM_002110.2	Endogenous	4.59	10.83	6.99	8.14	5.47	9.39	9.38	9.56	8.91	4.91	5.51	7.66
HLA-A	NM_002116.5	Endogenous	9481.24	18106.35	13208.78	18867.43	16725.16	15094.53	10306.32	13893.05	11551.83	12781.92	13939.84	12144.46
HLA-B	NM_005514.6	Endogenous	12070.54	17245.91	15623.93	22553.84	21032.98	17982.03	12799.83	17215.4	14813.99	16043.34	15369.18	13296.41
HLA-C	NM_002117.4	Endogenous	2360.67	4234.76	3788.26	4312	4274.08	4222.03	2529.69	4997.87	3898.55	3982.99	3731.26	3489.91
HLA-DMA	NM_006120.3	Endogenous	31.22	513.85	425.45	84.43	630.12	445.41	1.34	344.02	476.02	79.45	336.27	336.31
HLA-DMB	NM_002118.3	Endogenous	16.53	357.41	361.34	23.4	370.23	311.45	12.06	267.57	336.02	24.57	246.49	288.63
HLA-DOB	NM_002120.3	Endogenous	56.01	66.19	53.62	53.91	32.83	34.98	33.5	46.19	50.91	70.44	39.38	25.54
HLA-DPA1	NM_033554.2	Endogenous	209.35	2777.45	3523.66	815.81	2070.93	1562.36	42.88	2202.69	3427.62	327.62	1044.25	1283.92
HLA-DPB1	NM_002121.4	Endogenous	81.72	2140.85	2163.39	668.31	1876.69	1259.44	25.46	1175.41	1766.63	221.14	709.55	728.8
HLA-DQA1	NM_002122.3	Endogenous	11.94	12.03	16.32	12.21	22.8	24.75	32.16	3257.05	5056.79	635.57	1735.69	2465.67
HLA-DQB1	NM_002123.3	Endogenous	13.77	13.24	16.32	8.14	10.03	12.8	44.22	410.91	641.49	190.02	470.94	662.39
HLA-DRA	NM_019111.3	Endogenous	49.58	7595.86	9139.61	573.71	6624.95	7211.93	56.27	8081.32	12692.25	430.81	4127.38	6191.43
HLA-DRB3	NM_022555.3	Endogenous	53.26	2494.65	2452.46	821.92	2574.29	2342.25	16.08	1479.61	2222.29	402.15	1068.66	1415.04
HLA-DRB4	NM_021983.4	Endogenous	7.35	8.42	10.49	5.09	6.38	5.12	2.68	6.37	11.46	9.01	5.51	5.11
HLA-E	NM_005516.4	Endogenous	5449.46	4312.98	3525.99	10927	7998.28	7398.79	6199.61	3524.63	3419.98	10092.2	6822.26	6724.41
HLA-G	NM_002127.4	Endogenous	2818.85	5274.5	3954.94	6065.69	5576.27	4716.93	3069.66	3605.85	3249.43	4390.87	4818.03	4257.03
HMGB1	NM_002128.4	Endogenous	3639.71	7643.99	8978.75	2581.71	6288.46	7030.18	4038.39	8570.27	8592.6	2991.13	6605.69	7313.58
HRAS	NM_005343.2	Endogenous	155.17	134.78	146.87	142.41	113.08	127.99	167.48	146.53	155.28	161.35	107.89	143.04
HSD11B1	NM_181755.1	Endogenous	11.02	31.29	23.31	16.28	29.18	19.63	18.76	47.78	38.18	9.01	25.99	17.88
ICAM1	NM_000201.2	Endogenous	485.72	1125.18	885.87	965.34	424.95	861.81	728.89	1008.17	884.59	837.06	370.92	655.58
ICAM2	NM_000873.3	Endogenous	787.81	764.16	649.25	967.38	1160.85	1063.19	814.64	711.93	708.94	936.16	1081.26	1062.56
ICAM3	NM_002162.3	Endogenous	895.24	1575.25	1167.95	901.26	1894.02	1401.94	1011.61	1571.99	1370.79	891.93	1776.64	1447.39
ICAM4	NM_001039132.1	Endogenous	14.69	13.24	23.31	11.19	5.47	11.95	37.52	14.33	15.27	9.83	9.45	10.22
ICOS	NM_012092.2	Endogenous	3873.85	4615.04	4160.09	10587.24	8137.8	9066.96	5137.08	3769.9	4215.48	7504.04	5383.47	6940.66
ICOSLG	NM_015259.4	Endogenous	78.05	55.36	27.97	95.62	88.45	93.01	75.03	44.6	71.28	188.38	168.53	136.23
IDO1	NM_002164.3	Endogenous	38.56	22.86	13.99	41.71	33.74	33.28	56.27	27.08	40.73	88.46	33.08	23.84
IFI16	NM_005531.1	Endogenous	1900.66	2244.34	1969.89	1256.27	2124.73	1793.6	1656.09	1775.85	1434.43	1095.87	2049.12	1867.13
IFI27	NM_005532.3	Endogenous	11.94	316.49	13.99	9.15	14.59	5.12	49.58	1304.41	3.82	9.83	7.88	4.26
IFI35	NM_005533.3	Endogenous	200.17	369.44	284.41	432.32	301.84	254.28	322.91	549.48	258.38	374.3	334.69	240.1
IFIH1	NM_022168.2	Endogenous	97.33	270.76	137.54	283.8	212.47	216.73	131.31	278.72	118.37	212.13	196.09	183.05
IFIT1	NM_001548.3	Endogenous	1.84	6.02	4.66	11.19	8.21	13.65	1.34	30.26	6.36	12.29	7.88	14.47
IFIT2	NM_001547.4	Endogenous	9.18	1.2	1.17	10.17	19.15	19.63	1.34	1.59	1.27	32.76	25.2	17.88
IFITM1	NM_003641.3	Endogenous	3929.86	4887	2875.58	2690.55	1333.2	1119.5	2595.34	4735.07	1936.46	3694.69	1649.85	1592.13
IFITM2	NM_006435.2	Endogenous	1941.06	2046.98	1360.28	1371.21	760.52	638.25	1472.52	1938.31	990.23	1530.78	716.64	706.67
IFNA1	NM_024013.1	Endogenous	5.51	9.63	13.99	5.09	7.3	4.27	6.7	6.37	5.09	3.28	3.94	5.96
IFNA17	NM_021268.2	Endogenous	2.75	7.22	9.32	7.12	7.3	8.53	5.36	1.59	6.36	8.19	1	11.07
IFNA2	NM_000605.3	Endogenous	2.75	7.22	4.66	7.12	12.77	5.97	6.7	1.59	7.64	8.19	4.73	5.96
IFNA7	NM_021057.2	Endogenous	6.43	14.44	9.32	6.1	10.03	13.65	9.38	19.11	14	6.55	6.3	6.81
IFNA8	NM_002170.3	Endogenous	7.35	3.61	12.82	7.12	7.3	5.12	6.7	14.33	3.82	3.28	3.15	1.7
IFNAR1	NM_000629.2	Endogenous	145.07	137.19	141.04	149.53	166.88	137.38	128.63	125.82	115.82	150.7	166.95	121.75
IFNAR2	NM_000874.3	Endogenous	416.86	497	503.55	505.56	1421.65	663.85	385.88	562.22	530.75	466.03	1581.33	872.69
IFNB1	NM_002176.2	Endogenous	1.84	4.81	5.83	7.12	5.47	6.83	2.68	4.78	7.64	4.91	3.15	3.41
IFNG	NM_000619.2	Endogenous	25086.82	17397.54	16318.64	1824.9	101.22	186.87	34110.56	12239.83	7509.46	1543.07	73.24	406.12
IFNGR1	NM_000416.1	Endogenous	555.51	429.61	398.64	548.28	295.46	262.81	514.51	442.77	448.02	528.28	386.67	338.86
IFNL1	NM_172140.1	Endogenous	32.14	28.88	23.31	37.64	34.65	28.16	25.46	23.89	20.36	31.12	29.93	19.58
IFNL2	NM_172138.1	Endogenous	5.51	9.63	10.49	8.14	12.77	11.09	8.04	15.93	17.82	13.1	5.51	2.55
IGF1R	NM_000875.2	Endogenous	374.62	196.15	254.1	135.29	244.39	190.28	368.47	232.53	164.19	290.76	207.12	222.22
IGF2R	NM_000876.1	Endogenous	472.87	504.22	518.7	948.05	727.7	997.48	493.07	436.4	379.29	883.74	542.6	767.97
IGLL1	NM_020070.2	Endogenous	1	1.2	1.17	1.02	5.47	2.56	1.34	1.59	1.27	1.64	1	1
IKBKB	NM_001556.1	Endogenous	215.77	424.8	304.23	195.31	277.22	213.32	171.5	356.76	357.65	169.54	256.73	217.11
IKBKE	NM_014002.2	Endogenous	116.61	322.51	286.74	476.06	806.12	674.94	129.97	385.43	213.83	443.1	755.23	606.2
IKBKG	NM_003639.2	Endogenous	374.62	423.6	354.35	524.89	326.46	323.39	333.63	374.28	314.38	384.13	385.88	322.68
IL10	NM_000572.2	Endogenous	1240.48	2142.05	617.78	1425.13	14.59	23.04	2352.82	1772.67	1481.53	1036.9	24.41	72.37
IL10RA	NM_001558.2	Endogenous	910.85	1430.84	1315.98	1268.48	977.56	515.38	952.65	1164.26	1153.15	933.7	919.82	447.84
IL11	NM_000641.2	Endogenous	8.26	12.03	12.82	16.28	12.77	11.09	16.08	11.15	17.82	13.1	7.09	1.7
IL11RA	NM_147162.1	Endogenous	31.22	72.2	68.77	53.91	102.13	91.3	40.2	58.93	43.27	37.68	72.45	38.31
IL12A	NM_000882.2	Endogenous	4.59	30.08	43.13	14.24	14.59	9.39	1.34	28.67	22.91	11.47	9.45	12.77
IL12B	NM_002187.2	Endogenous	7.35	4.81	8.16	4.07	9.12	10.24	4.02	3.19	12.73	10.65	4.73	12.77
IL12RB1	NM_005535.1	Endogenous	101	204.58	170.18	277.7	325.55	237.21	124.61	230.94	161.64	253.9	337.06	240.1
IL12RB2	NM_001559.2	Endogenous	943.9	1562.01	1591.07	1564.49	1532.9	1967.66	998.21	1341.05	1370.79	1525.05	1848.3	2200.03
IL13	NM_002188.2	Endogenous	451.75	15920.97	9845.97	202.43	39.21	59.73	865.56	12878.5	10303.23	99.92	20.48	27.25
IL13RA1	NM_001560.2	Endogenous	21.12	28.88	25.64	10.17	12.77	11.95	33.5	44.6	36.91	13.92	10.24	12.77
IL13RA2	NM_000640.2	Endogenous	6.43	7.22	8.16	8.14	3.65	13.65	12.06	17.52	8.91	4.91	3.15	6.81
IL15	NM_172174.1	Endogenous	18.36	27.68	29.14	18.31	19.15	11.95	21.44	35.04	17.82	10.65	18.9	12.77
IL15RA	NM_002189.2	Endogenous	412.27	559.58	430.11	910.41	310.05	411.28	416.7	447.55	352.56	742.05	317.37	359.29
IL16	NM_004513.4	Endogenous	499.5	818.31	754.15	462.84	2353.61	1436.92	419.38	836.16	815.86	620.01	2686.22	1819.46
IL17A	NM_002190.2	Endogenous	886.05	216.61	228.46	323.48	3.65	8.53	1004.91	648.23	333.47	337.44	11.03	10.22
IL17B	NM_014443.2	Endogenous	5.51	8.42	17.48	4.07	6.38	5.97	9.38	17.52	15.27	6.55	6.3	8.51
IL17F	NM_052872.3	Endogenous	2873.94	518.67	610.78	66.12	8.21	5.12	10626.55	1533.76	940.59	265.37	7.09	5.11
IL17RA	NM_014339.6	Endogenous	189.15	129.97	149.2	211.58	173.26	137.38	265.3	130.6	152.73	185.92	155.93	137.93
IL17RB	NM_018725.3	Endogenous	8.26	1166.09	240.12	11.19	24.62	17.07	1.34	917.39	344.93	1	1	1
IL18	NM_001562.2	Endogenous	11.02	10.83	11.66	13.22	14.59	7.68	12.06	20.7	11.46	5.73	8.66	6.81
IL18R1	NM_003855.2	Endogenous	416.86	210.59	217.97	1496.33	1485.49	1956.57	448.86	286.68	194.74	1252.31	1421.47	1773.48
IL18RAP	NM_003853.2	Endogenous	65.19	36.1	37.3	53.91	30.09	26.45	48.24	44.6	17.82	24.57	36.23	25.54
IL19	NM_013371.3	Endogenous	6.43	10.83	8.16	12.21	3.65	10.24	14.74	7.96	7.64	9.83	9.45	5.11
IL1A	NM_000575.3	Endogenous	27.55	710.01	1433.71	84.43	193.32	145.06	32.16	726.27	2023.73	17.2	112.61	71.52
IL1B	NM_000576.2	Endogenous	8.26	6.02	10.49	19.33	10.94	10.24	16.08	6.37	10.18	36.86	7.88	5.11
IL1R1	NM_000877.2	Endogenous	105.59	10.83	11.66	1336.63	522.52	728.7	151.41	22.3	16.55	887.02	434.71	557.67
IL1R2	NM_173343.1	Endogenous	121.2	33.7	48.96	1025.36	797.91	1276.51	154.09	30.26	42	606.91	621.35	803.73
IL1RAP	NM_002182.2	Endogenous	95.49	641.41	650.41	408.92	147.73	157	154.09	490.55	640.21	280.11	129.94	189.01
IL1RAPL2	NM_017416.1	Endogenous	23.87	26.47	25.64	15.26	16.41	23.89	42.88	31.85	24.18	19.66	14.18	22.99
IL1RL1	NM_016232.4	Endogenous	22.95	22.86	32.64	18.31	18.24	19.63	37.52	57.34	56	18.84	25.99	22.14
IL1RL2	NM_003854.2	Endogenous	10.1	7.22	8.16	29.5	8.21	11.09	9.38	4.78	11.46	27.85	9.45	12.77
IL1RN	NM_000577.3	Endogenous	14.69	55.36	32.64	277.7	71.13	86.18	29.48	25.48	19.09	109.75	16.54	28.95
IL2	NM_000586.2	Endogenous	71477.51	2332.19	4019.05	704.93	41.04	78.5	40346.34	3323.95	3366.53	950.9	36.23	61.3
IL21	NM_021803.2	Endogenous	5655.14	1781.03	3208.94	162.76	7.3	10.24	2472.07	1482.8	2207.02	131.05	18.9	14.47
IL21R	NM_021798.2	Endogenous	669.36	599.29	423.12	1634.68	1339.58	1510.31	610.98	619.56	445.48	1558.63	1186	1438.88
IL22	NM_020525.4	Endogenous	101.92	1577.66	1596.9	22.38	10.94	10.24	245.2	1694.62	1300.79	32.76	9.45	6.81
IL22RA1	NM_021258.2	Endogenous	3.67	12.03	3.5	8.14	4.56	7.68	4.02	7.96	6.36	7.37	6.3	6.81
IL22RA2	NM_181310.1	Endogenous	13.77	8.42	8.16	6.1	9.12	6.83	28.14	20.7	14	7.37	8.66	6.81
IL23A	NM_016584.2	Endogenous	865.85	509.04	522.2	1791.33	337.4	364.35	860.2	356.76	440.38	956.64	360.68	366.96
IL23R	NM_144701.2	Endogenous	116.61	57.76	153.86	43.74	147.73	252.57	168.82	93.97	131.1	36.04	81.11	147.29
IL24	NM_181339.1	Endogenous	163.44	37.31	38.47	48.83	15.5	9.39	136.67	38.22	50.91	43.41	11.03	10.22
IL25	NM_022789.2	Endogenous	7.35	8.42	3.5	8.14	7.3	1.71	2.68	11.15	11.46	6.55	3.15	5.96
IL26	NM_018402.1	Endogenous	23.87	770.18	547.84	24.41	7.3	8.53	28.14	415.69	287.65	12.29	7.09	7.66
IL27	NM_145659.3	Endogenous	10.1	4.81	17.48	4.07	11.85	12.8	9.38	7.96	15.27	7.37	7.09	4.26
IL2RA	NM_000417.1	Endogenous	21195.53	44137.08	29549.56	42284.89	26677.64	31137.91	32581.76	38113.11	33662.72	36665.11	29000.31	32873.65
IL2RB	NM_000878.2	Endogenous	1063.27	4386.39	3097.05	5210.21	6038.6	4046.26	1677.52	3473.66	3204.88	4523.55	5521.28	3977.77
IL2RG	NM_000206.1	Endogenous	5535.77	10691	6476.17	9536.45	10485.03	8207.71	6154.05	7872.67	5907.01	7056.84	9986.5	7872.1
IL3	NM_000588.3	Endogenous	3546.05	2209.44	2389.52	56.96	10.94	11.95	6018.72	2169.25	1598.62	40.95	3.15	7.66
IL32	NM_004221.4	Endogenous	11386.49	41182.73	26405.89	18952.88	24288.46	17638.16	16437.6	32705.92	26230.91	20964.12	29257.83	24345.11
IL34	NM_152456.1	Endogenous	3.67	6.02	3.5	4.07	9.12	4.27	10.72	6.37	10.18	4.91	5.51	4.26
IL3RA	NM_002183.2	Endogenous	11.02	265.95	75.77	23.4	19.15	12.8	32.16	430.03	96.73	18.84	7.88	5.11
IL4	NM_000589.2	Endogenous	291.98	3394.79	1728.61	142.41	16.41	5.97	436.8	2220.21	1834.09	93.37	4.73	7.66
IL4R	NM_000418.2	Endogenous	1200.08	1413.99	1170.28	1305.1	1738.99	1790.18	1125.5	1149.92	1022.05	1282.62	1822.31	2037.42
IL5	NM_000879.2	Endogenous	127.63	12879.98	7255.97	215.65	75.69	80.21	99.15	8987.56	5947.74	60.61	10.24	14.47
IL5RA	NM_000564.3	Endogenous	2.75	9.63	3.5	2.03	10.03	11.09	4.02	4.78	11.46	1	9.45	3.41
IL6	NM_000600.1	Endogenous	118.45	31.29	41.96	11.19	9.12	17.07	143.37	57.34	36.91	26.21	9.45	8.51
IL6R	NM_000565.2	Endogenous	318.61	140.8	127.05	1055.88	219.77	176.63	263.96	109.9	148.92	705.19	141.75	140.48
IL6ST	NM_002184.2	Endogenous	1359.84	658.26	589.8	1285.77	795.18	511.12	1514.06	684.86	686.03	1231.02	1106.46	601.94
IL7	NM_000880.2	Endogenous	5.51	32.49	20.98	28.48	34.65	14.51	13.4	30.26	19.09	13.92	20.48	11.07
IL7R	NM_002185.2	Endogenous	3042.89	762.96	1677.32	197.34	130.4	86.18	2486.81	633.89	1125.15	301.41	122.85	91.1
IL8	NM_000584.2	Endogenous	33.97	997.62	1816.03	96.64	18.24	29.01	79.05	345.61	496.39	194.11	15.75	29.8
IL9	NM_000590.1	Endogenous	23610.37	2710.06	15282.41	775.12	12.77	12.8	22101.25	2565.83	11248.91	68.8	9.45	5.96
ILF3	NM_001137673.1	Endogenous	4686.45	4831.65	4880.44	4463.57	3945.79	4764.72	4715.02	4980.35	5126.79	5226.29	4326.62	5002.01
INPP5D	NM_005541.3	Endogenous	416.86	726.85	600.29	960.26	1037.74	690.3	401.96	492.14	534.57	809.21	1156.07	808.84
IRAK1	NM_001569.3	Endogenous	1100.91	1037.33	982.62	587.95	445.92	511.12	1305.04	1154.7	1215.51	741.23	397.7	465.72
IRAK2	NM_001570.3	Endogenous	109.26	131.17	142.21	189.2	185.12	117.75	187.58	167.23	147.64	158.07	140.97	121.75
IRAK4	NM_016123.1	Endogenous	260.77	335.75	240.12	319.41	332.84	274.76	263.96	289.87	276.2	294.04	359.11	258.83
IRF1	NM_002198.1	Endogenous	382.89	555.97	435.94	1218.63	662.04	583.64	738.27	617.96	386.93	1051.65	752.87	628.34
IRF2	NM_002199.3	Endogenous	910.85	1501.84	1045.56	1100.63	1259.33	831.95	1073.24	1293.27	920.23	1008.24	1376.58	962.94
IRF3	NM_001571.5	Endogenous	166.19	169.68	180.67	151.57	205.18	157	163.46	186.34	178.19	143.33	176.4	141.33
IRF4	NM_002460.1	Endogenous	6116.07	7583.82	8211.77	6095.19	5641.93	5685.41	5798.98	7050.85	8951.52	4691.46	5553.57	5936
IRF5	NM_002200.3	Endogenous	38.56	110.71	96.75	80.36	124.93	169.8	42.88	119.45	103.1	52.42	115.76	137.08
IRF7	NM_001572.3	Endogenous	78.05	129.97	100.24	183.1	113.99	69.12	132.65	235.72	64.91	143.33	126	69.82
IRF8	NM_002163.2	Endogenous	1214.77	145.61	99.08	106.81	24.62	40.1	569.45	127.42	77.64	296.49	55.13	48.53
IRGM	NM_001145805.1	Endogenous	10.1	10.83	10.49	15.26	13.68	15.36	18.76	7.96	10.18	47.5	28.35	14.47
ISG15	NM_005101.3	Endogenous	173.54	471.73	255.27	751.73	654.74	379.71	176.86	477.81	174.37	488.15	477.24	362.7
ISG20	NM_002201.4	Endogenous	215.77	403.14	272.75	509.63	926.49	793.55	257.26	436.4	274.92	534.01	956.83	842.04

**TABLE S1. Gene expression of stimulated T cells (cont.)**														
ITCH	NM_001257138.1	Endogenous	1056.84	784.62	890.53	1217.61	918.28	810.62	1250.1	839.35	809.49	1092.6	911.94	870.14
ITGA1	NM_181501.1	Endogenous	23.87	34.9	12.82	10.17	19.15	17.07	48.24	25.48	10.18	31.12	11.81	10.22
ITGA2	NM_002203.2	Endogenous	47.75	253.92	124.72	20.34	30.09	13.65	21.44	121.04	33.09	8.19	22.05	11.07
ITGA2B	NM_000419.3	Endogenous	11.94	7.22	6.99	3.05	8.21	3.41	5.36	19.11	11.46	4.91	8.66	11.07
ITGA4	NM_000885.4	Endogenous	149.67	942.26	645.75	166.82	473.28	336.19	180.88	745.38	509.12	162.17	471.72	257.98
ITGA5	NM_002205.2	Endogenous	206.59	332.14	430.11	170.89	180.56	249.16	113.89	334.47	342.38	142.51	138.6	197.53
ITGA6	NM_000210.1	Endogenous	145.99	12.03	10.49	109.86	31	27.3	136.67	14.33	10.18	165.45	20.48	45.98
ITGAE	NM_002208.4	Endogenous	282.8	346.58	434.78	336.7	621.92	440.29	379.18	374.28	384.38	405.42	641.04	521.06
ITGAL	NM_002209.2	Endogenous	763.94	2818.36	1936.09	805.64	2497.69	1230.43	1243.4	2368.33	1799.72	823.13	2479.1	1266.89
ITGAM	NM_000632.3	Endogenous	8.26	73.41	36.13	25.43	273.57	188.57	20.1	35.04	14	11.47	238.62	212
ITGAX	NM_000887.3	Endogenous	11.94	459.7	305.39	24.41	104.87	41.81	18.76	245.27	131.1	31.12	88.99	42.57
ITGB1	NM_033666.2	Endogenous	1857.5	2156.49	2041	2982.49	1363.29	1453.14	1806.15	1538.54	1879.91	1609.41	1005.66	1457.61
ITGB2	NM_000211.2	Endogenous	693.23	1395.94	996.6	920.59	977.56	1035.03	573.47	1019.32	899.86	959.91	1089.92	1112.79
ITGB3	NM_000212.2	Endogenous	4.59	3.61	5.83	4.07	5.47	5.97	1.34	7.96	5.09	4.91	7.09	1
ITGB4	NM_001005731.1	Endogenous	7.35	13.24	11.66	7.12	9.12	8.53	17.42	17.52	16.55	5.73	7.88	7.66
ITK	NM_005546.3	Endogenous	5277.76	3120.41	2502.58	7684.09	3562.79	3506.98	4851.69	2191.54	2065.74	5985.54	3430.42	3415.84
JAK1	NM_002227.1	Endogenous	1065.1	1308.1	1216.9	2846.19	2175.79	1698.88	1243.4	1221.59	1156.97	2156.53	2094.79	1732.61
JAK2	NM_004972.2	Endogenous	538.98	690.75	712.19	953.14	703.07	706.52	419.38	568.59	548.57	814.12	632.38	659.84
JAK3	NM_000215.2	Endogenous	976.96	1901.37	1788.06	1387.49	1257.51	1231.28	1054.48	1732.85	1555.35	1188.43	1126.94	1199.63
JAM3	NM_032801.3	Endogenous	151.5	54.15	58.28	55.95	34.65	48.64	125.95	36.63	57.28	95.83	34.65	40.87
KIR3DL1	NM_013289.2	Endogenous	10.1	20.46	23.31	5.09	11.85	10.24	40.2	43	25.46	6.55	6.3	11.92
KIR3DL2	NM_006737.2	Endogenous	14.69	16.85	8.16	5.09	11.85	15.36	28.14	23.89	36.91	9.83	7.09	5.11
KIR3DL3	NM_153443.3	Endogenous	11.02	9.63	4.66	6.1	6.38	9.39	8.04	6.37	15.27	4.1	11.03	7.66
KIR_Activating_Subgroup_1	NM_001083539.1	Endogenous	9.18	6.02	1.17	11.19	7.3	7.68	14.74	7.96	1.27	5.73	3.94	1
KIR_Activating_Subgroup_2	NM_014512.1	Endogenous	10.1	19.25	17.48	10.17	16.41	10.24	18.76	20.7	15.27	9.83	11.81	12.77
KIR_Inhibiting_Subgroup_1	NM_014218.2	Endogenous	18.36	15.64	22.15	16.28	13.68	14.51	32.16	23.89	26.73	9.83	9.45	8.51
KIR_Inhibiting_Subgroup_2	NM_014511.3	Endogenous	8.26	15.64	17.48	15.26	10.03	7.68	16.08	27.08	21.64	13.92	7.09	6.81
KIT	NM_000222.2	Endogenous	7.35	6.02	13.99	4.07	5.47	4.27	4.02	6.37	6.36	1.64	1	3.41
KLRB1	NM_002258.2	Endogenous	56.01	105.9	60.61	20.34	48.33	14.51	124.61	152.9	56	18.84	29.14	6.81
KLRC1	NM_002259.3	Endogenous	9.18	49.34	33.8	3.05	22.8	14.51	1.34	9.56	5.09	3.28	1	1
KLRC2	NM_002260.3	Endogenous	9.18	42.12	23.31	10.17	19.15	6.83	5.36	4.78	7.64	4.1	6.3	7.66
KLRD1	NM_002262.3	Endogenous	15.61	24.07	36.13	6.1	22.8	5.12	30.82	11.15	19.09	4.1	3.15	3.41
KLRF1	NM_016523.1	Endogenous	1	12.03	5.83	7.12	8.21	1.71	2.68	6.37	7.64	4.91	1	3.41
KLRG1	NM_005810.3	Endogenous	4.59	13.24	15.15	5.09	10.03	8.53	12.06	9.56	14	9.83	11.03	7.66
KLRK1	NM_007360.3	Endogenous	18.36	20.46	25.64	9.15	23.71	8.53	9.38	31.85	6.36	4.1	9.45	7.66
LAG3	NM_002286.5	Endogenous	1212.93	8508.03	3651.88	859.55	1866.66	1173.26	1585.07	6870.87	4139.11	645.4	1363.19	1159.62
LAIR2	NM_002288.3	Endogenous	14.69	102.29	37.3	104.77	934.7	605.83	16.08	76.45	67.46	117.12	901.71	570.44
LAMP1	NM_005561.3	Endogenous	1401.16	900.14	741.33	1496.33	965.7	959.09	1463.14	848.9	809.49	1602.86	1069.45	1023.39
LAMP2	NM_001122606.1	Endogenous	234.14	356.21	300.73	420.11	331.02	311.45	293.43	326.5	299.11	277.65	311.07	298.84
LAMP3	NM_014398.3	Endogenous	102.84	60.17	15.15	154.62	83.89	81.06	77.71	36.63	36.91	155.62	112.61	109.83
LBP	NM_004139.2	Endogenous	5.51	10.83	5.83	4.07	5.47	8.53	8.04	7.96	8.91	6.55	10.24	8.51
LCK	NM_005356.2	Endogenous	2388.22	3007.29	2641.29	3161.52	3577.38	2486.46	2244.29	2441.6	2354.66	2856.81	3620.22	2603.6
LCN2	NM_005564.3	Endogenous	5.51	6.02	5.83	7.12	9.12	5.12	5.36	11.15	10.18	6.55	1	5.11
LCP1	NM_002298.4	Endogenous	8523.57	11737.96	9431.01	8621.97	14252.09	16106.52	8165.2	10913.12	9118.26	10301.05	13455.51	14421.97
LGALS3	NM_001177388.1	Endogenous	203.84	1769	1221.57	1359.01	1737.17	1778.24	751.67	1326.71	1452.25	841.15	1478.17	1256.68
LIF	NM_002309.3	Endogenous	1239.56	7958.08	8159.32	709	507.93	462.48	1349.25	7079.51	6890.88	580.7	429.2	600.24
LILRA1	NM_006863.1	Endogenous	9.18	8.42	10.49	8.14	10.94	9.39	18.76	25.48	10.18	6.55	9.45	6.81
LILRA4	NM_012276.3	Endogenous	1	7.22	1.17	3.05	2.74	6.83	5.36	4.78	1.27	1.64	2.36	5.11
LILRA5	NM_181879.2	Endogenous	8.26	26.47	19.82	8.14	4.56	9.39	14.74	20.7	20.36	7.37	10.24	2.55
LILRB1	NM_001081637.1	Endogenous	9.18	9.63	6.99	8.14	11.85	5.97	13.4	7.96	6.36	9.01	8.66	5.11
LILRB2	NM_005874.1	Endogenous	8.26	6.02	9.32	9.15	10.03	8.53	13.4	12.74	12.73	4.1	4.73	6.81
LILRB3	NM_006864.2	Endogenous	3.67	3.61	9.32	9.15	3.65	2.56	2.68	6.37	6.36	4.91	2.36	3.41
LRP1	NM_002332.2	Endogenous	10.1	9.63	12.82	4.07	23.71	12.8	6.7	12.74	17.82	9.01	17.33	13.62
LRRN3	NM_001099660.1	Endogenous	247.91	607.72	505.88	22.38	83.89	25.6	391.24	963.58	437.84	94.19	359.9	194.12
LTA	NM_000595.2	Endogenous	25927.88	19226.71	22322.74	5355.67	4568.62	11148.11	22594.32	16943.05	18146.15	6945.45	4525.07	7532.39
LTB	NM_002341.1	Endogenous	974.2	2323.76	2051.49	1156.58	2806.83	1864.42	1286.28	1998.83	1585.9	1458.71	2890.97	1737.72
LTBR	NM_002342.1	Endogenous	13.77	21.66	19.82	8.14	16.41	11.95	10.72	17.52	19.09	15.56	11.03	11.07
LTF	NM_002343.2	Endogenous	7.35	15.64	20.98	14.24	17.33	12.8	13.4	20.7	22.91	10.65	17.33	16.18
LTK	NM_001135685.1	Endogenous	6.43	9.63	9.32	18.31	11.85	8.53	16.08	22.3	11.46	13.92	18.11	10.22
LY86	NM_004271.3	Endogenous	11.02	9.63	10.49	10.17	8.21	15.36	17.42	15.93	12.73	3.28	12.6	10.22
LY9	NM_001033667.1	Endogenous	136.81	19.25	58.28	81.38	221.59	60.58	128.63	28.67	50.91	114.67	255.94	94.51
LY96	NM_015364.2	Endogenous	114.77	172.09	125.89	69.17	142.26	66.56	190.26	184.75	129.82	62.25	169.32	99.61
LYN	NM_002350.1	Endogenous	14.69	63.78	67.61	12.21	29.18	22.19	26.8	63.71	53.46	7.37	32.29	22.14
MAF	NM_005360.4	Endogenous	2323.94	3376.74	2128.42	2563.4	833.48	313.15	3045.54	2505.3	2470.48	1253.13	704.04	269.9
MAGEA1	NM_004988.4	Endogenous	5.51	8.42	8.16	4.07	7.3	2.56	9.38	6.37	2.55	3.28	2.36	6.81
MAGEA12	NM_001166386.1	Endogenous	8.26	4.81	10.49	2.03	5.47	7.68	9.38	11.15	12.73	9.83	4.73	4.26
MAGEA3	NM_005362.3	Endogenous	9.18	19.25	4.66	9.15	3.65	6.83	9.38	9.56	6.36	4.1	6.3	7.66
MAGEA4	NM_001011548.1	Endogenous	4.59	13.24	9.32	3.05	10.94	3.41	1.34	6.37	8.91	6.55	3.94	4.26
MAGEB2	NM_002364.4	Endogenous	11.02	14.44	15.15	6.1	6.38	12.8	28.14	15.93	19.09	5.73	17.33	15.33
MAGEC1	NM_005462.4	Endogenous	2.75	4.81	4.66	5.09	7.3	2.56	8.04	6.37	6.36	3.28	4.73	3.41
MAGEC2	NM_016249.3	Endogenous	4.59	9.63	6.99	5.09	6.38	6.83	6.7	9.56	5.09	4.1	5.51	9.37
MAP2K1	NM_002755.2	Endogenous	2181.62	2849.65	2734.54	2049.7	2196.77	2763.78	1897.26	2350.81	2609.22	1700.33	2300.34	2810.49
MAP2K2	NM_030662.2	Endogenous	1599.49	2451.32	2149.4	1469.89	1881.25	1880.63	1866.45	2522.82	2341.93	1520.14	1872.72	1936.1
MAP2K4	NM_003010.2	Endogenous	289.23	306.87	229.63	385.53	373.88	366.06	330.95	216.61	252.01	348.91	370.13	365.25
MAP3K1	NM_005921.1	Endogenous	416.86	636.6	446.43	475.04	646.54	493.2	431.44	517.62	439.11	411.16	637.1	483.6
MAP3K5	NM_005923.3	Endogenous	270.87	500.61	282.08	671.37	407.62	323.39	380.52	492.14	264.74	556.13	448.1	355.04
MAP3K7	NM_145333.1	Endogenous	888.81	966.33	929	876.85	1081.51	1017.11	775.79	893.5	987.68	840.33	1109.61	1054.89
MAP4K2	NM_004579.2	Endogenous	496.74	945.87	1021.08	719.18	1195.5	1103.29	379.18	826.61	977.5	552.03	1022.98	1037.86
MAPK1	NM_138957.2	Endogenous	1310.26	616.14	694.71	911.43	813.41	680.92	1280.92	648.23	747.13	914.87	924.54	762.86
MAPK11	NM_002751.5	Endogenous	39.48	54.15	64.11	29.5	38.3	48.64	34.84	73.26	71.28	27.85	40.16	48.53
MAPK14	NM_001315.1	Endogenous	587.64	703.99	618.94	700.87	812.5	764.54	755.69	729.45	669.49	647.04	805.63	735.62
MAPK3	NM_001040056.1	Endogenous	395.74	882.09	725.01	434.35	736.82	572.55	347.03	699.19	674.58	303.86	616.63	543.2
MAPK8	NM_002750.2	Endogenous	578.46	866.45	719.19	540.14	391.21	440.29	584.19	777.23	623.67	477.5	423.68	464.02
MAPKAPK2	NM_004759.3	Endogenous	658.34	726.85	573.48	662.21	434.98	435.17	728.89	729.45	562.57	533.19	411.87	402.72
MARCO	NM_006770.3	Endogenous	11.02	6.02	15.15	11.19	11.85	12.8	12.06	9.56	12.73	11.47	10.24	10.22
MASP1	NM_139125.3	Endogenous	5.51	13.24	6.99	4.07	4.56	5.97	10.72	15.93	11.46	4.91	3.15	5.11
MASP2	NM_139208.1	Endogenous	38.56	50.54	34.97	45.77	41.04	36.69	25.46	41.41	33.09	36.04	29.14	32.35
MAVS	NM_020746.3	Endogenous	493.07	528.29	490.72	574.73	576.32	450.53	418.04	457.1	496.39	520.09	600.88	471.68
MBL2	NM_000242.2	Endogenous	11.94	4.81	8.16	9.15	6.38	5.12	16.08	11.15	8.91	11.47	4.73	6.81
MCAM	NM_006500.2	Endogenous	22.95	46.93	41.96	26.45	38.3	37.54	37.52	54.15	40.73	25.39	37.01	42.57
MEF2C	NM_002397.3	Endogenous	6.43	12.03	11.66	10.17	14.59	6.83	17.42	27.08	20.36	8.19	14.18	5.96
MEFV	NM_000243.2	Endogenous	9.18	93.87	120.06	29.5	67.48	59.73	8.04	58.93	118.37	14.74	43.31	41.72
MERTK	NM_006343.2	Endogenous	38.56	34.9	40.8	15.26	29.18	38.4	22.78	33.45	47.09	14.74	22.84	27.25
MFGE8	NM_001114614.1	Endogenous	29.38	127.56	135.21	70.19	208.83	173.22	24.12	95.56	115.82	48.32	165.38	166.88
MICA	NM_000247.1	Endogenous	151.5	96.27	85.09	140.38	96.66	116.05	69.67	100.34	68.73	76.17	77.96	61.3
MICB	NM_005931.3	Endogenous	626.21	773.79	674.89	637.8	621.92	684.33	535.95	724.67	608.39	669.15	741.05	758.6
MIF	NM_002415.1	Endogenous	4879.27	8716.22	7469.28	3914.27	7883.38	8838.28	5654.28	6725.94	7612.55	5317.2	8198.84	9010.43
MME	NM_000902.2	Endogenous	6.43	6.02	12.82	7.12	2.74	11.95	9.38	12.74	7.64	8.19	3.94	5.96
MNX1	NM_005515.3	Endogenous	9.18	12.03	10.49	5.09	9.12	5.97	8.04	7.96	7.64	9.01	6.3	6.81
MPPED1	NM_001044370.1	Endogenous	39.48	72.2	67.61	52.9	60.19	64	61.63	62.11	72.55	44.23	56.7	51.94
MR1	NM_001531.2	Endogenous	30.3	63.78	44.29	52.9	85.72	48.64	49.58	71.67	33.09	45.87	88.99	48.53
MRC1	NM_002438.2	Endogenous	6.43	8.42	10.49	40.69	40.12	16.21	12.06	15.93	8.91	31.94	27.56	17.03
MS4A1	NM_152866.2	Endogenous	2.75	4.81	8.16	6.1	11.85	4.27	13.4	6.37	7.64	4.1	9.45	1.7
MS4A2	NM_000139.3	Endogenous	6.43	8.42	9.32	9.15	6.38	11.95	10.72	12.74	7.64	6.55	10.24	5.96
MSR1	NM_002445.3	Endogenous	2.75	3.61	6.99	3.05	2.74	4.27	9.38	6.37	10.18	9.83	4.73	3.41
MST1R	NM_002447.1	Endogenous	6.43	9.63	11.66	16.28	22.8	11.09	13.4	20.7	15.27	10.65	14.18	8.51
MUC1	NM_001018017.1	Endogenous	23.87	51.75	72.27	32.55	57.45	87.89	29.48	98.75	129.82	42.59	70.88	113.24
MX1	NM_002462.2	Endogenous	96.41	1020.48	55.95	456.73	230.71	131.41	292.09	3312.8	84	672.43	411.87	212
MYD88	NM_002468.3	Endogenous	427.88	452.48	412.63	503.52	495.16	491.49	494.41	496.92	460.75	547.94	538.66	498.07
NCAM1	NM_000615.5	Endogenous	10.1	19.25	12.82	12.21	20.97	10.24	5.36	9.56	17.82	11.47	10.24	5.11
NCF4	NM_000631.4	Endogenous	27.55	261.14	164.35	66.12	385.73	184.31	42.88	269.16	157.83	48.32	389.82	223.92
NCR1	NM_004829.5	Endogenous	1.84	10.83	9.32	7.12	9.12	5.97	5.36	7.96	7.64	8.19	11.03	10.22
NEFL	NM_006158.3	Endogenous	12.85	1.2	1.17	2.03	10.94	5.97	10.72	1.59	1.27	4.91	8.66	6.81
NFATC1	NM_172389.1	Endogenous	512.35	422.39	362.51	229.89	469.63	384.83	552.03	490.55	394.56	306.32	460.7	353.33
NFATC2	NM_012340.3	Endogenous	1736.3	1584.88	1453.52	2177.87	1174.53	1494.95	1808.83	1536.95	1445.89	1620.88	948.96	1400.56
NFATC3	NM_004555.2	Endogenous	1245.99	2191.39	1627.2	1014.17	917.37	744.91	1256.8	1863.45	1524.8	1008.24	948.17	869.29
NFATC4	NM_001136022.2	Endogenous	5.51	8.42	13.99	10.17	9.12	8.53	5.36	12.74	8.91	9.83	8.66	5.11
NFKB1	NM_003998.2	Endogenous	1885.96	708.8	743.66	1883.89	1154.47	1670.72	1744.52	659.37	712.76	1773.22	1178.13	1550.41
NFKB2	NM_002502.2	Endogenous	2173.36	2091.51	1692.48	3030.3	1862.1	2995.87	2660.99	2202.69	1764.09	2900.22	1819.95	2657.24
NFKBIA	NM_020529.1	Endogenous	9914.63	10904	10094.25	17187.99	10323.62	16807.92	15718.08	11613.91	9850.11	13492.03	9788.83	13459.88
NLRC5	NM_032206.4	Endogenous	190.98	269.56	270.42	432.32	403.97	430.05	223.76	315.35	333.47	395.6	429.98	408.68
NLRP3	NM_001079821.2	Endogenous	35.81	14.44	15.15	13.22	15.5	13.65	41.54	15.93	21.64	18.84	14.18	17.03
NOD1	NM_006092.1	Endogenous	38.56	113.12	88.59	68.15	103.04	69.97	46.9	74.86	63.64	56.51	114.98	74.92
NOD2	NM_022162.1	Endogenous	139.57	173.29	55.95	113.93	82.07	44.37	57.61	108.3	38.18	92.55	37.01	10.22
NOS2A	NM_153292.1	Endogenous	7.35	2.41	6.99	8.14	2.74	3.41	12.06	12.74	6.36	4.1	10.24	7.66
NOTCH1	NM_017617.3	Endogenous	443.49	365.83	334.53	536.08	355.64	236.36	463.6	356.76	353.84	459.48	307.13	249.46
NRP1	NM_003873.5	Endogenous	39.48	79.42	57.12	13.22	23.71	17.92	30.82	71.67	28	10.65	18.9	5.96
NT5E	NM_002526.2	Endogenous	63.36	32.49	32.64	60.02	59.27	18.77	123.27	82.82	43.27	50.78	48.04	11.07
NUP107	NM_020401.2	Endogenous	1641.73	2243.14	2427.98	1118.94	1645.07	2090.54	1644.03	2247.29	2515.03	1530.78	1908.94	2120.85
OAS3	NM_006187.2	Endogenous	45.91	258.73	93.25	135.29	204.27	123.73	54.93	549.48	96.73	148.25	192.15	140.48
OSM	NM_020530.4	Endogenous	81.72	652.24	549.01	87.48	77.51	65.7	147.39	638.67	529.48	95.01	58.28	83.44
PASD1	NM_173493.2	Endogenous	2.75	14.44	6.99	6.1	4.56	7.68	10.72	11.15	2.55	5.73	9.45	6.81
PAX5	NM_016734.1	Endogenous	8.26	8.42	3.5	1.02	6.38	8.53	10.72	7.96	16.55	6.55	3.15	2.55
PBK	NM_018492.2	Endogenous	52.34	710.01	853.23	45.77	506.1	753.45	91.11	734.23	818.4	80.27	575.67	791.81
PDCD1	NM_005018.1	Endogenous	465.52	632.99	431.28	1020.27	659.3	414.69	539.97	678.49	595.67	742.87	488.26	347.37
PDCD1LG2	NM_025239.3	Endogenous	29.38	21.66	16.32	19.33	12.77	9.39	24.12	27.08	24.18	12.29	10.24	11.07
PDGFC	NM_016205.2	Endogenous	4.59	7.22	8.16	3.05	4.56	7.68	6.7	12.74	2.55	5.73	3.15	4.26
PDGFRB	NM_002609.3	Endogenous	6.43	22.86	9.32	14.24	11.85	10.24	10.72	19.11	15.27	4.91	6.3	11.92
PECAM1	NM_000442.3	Endogenous	28.46	36.1	20.98	16.28	37.39	12.8	24.12	43	25.46	33.58	40.16	27.25
PIK3CD	NM_005026.3	Endogenous	1579.29	2046.98	1831.18	841.24	979.38	753.45	1756.58	2613.61	2069.55	891.93	1012.75	940.8
PIK3CG	NM_002649.2	Endogenous	373.7	610.12	234.29	71.21	206.09	61.44	451.54	594.07	207.46	51.6	222.08	84.29

TABLE S1. Gene expression of stimulated T cells (cont.)														
PIN1	NM_006221.2	Endogenous	367.28	558.38	556	347.89	530.73	551.22	531.93	635.48	720.4	347.27	539.45	525.32
PLA2G1B	NM_000928.2	Endogenous	4.59	8.42	9.32	2.03	7.3	4.27	5.36	7.96	7.64	4.1	4.73	6.81
PLA2G6	NM_001004426.1	Endogenous	57.85	121.54	96.75	57.98	131.31	77.65	49.58	114.67	94.19	50.78	109.46	66.41
PLAU	NM_002658.2	Endogenous	5.51	24.07	27.97	13.22	28.27	69.97	14.74	27.08	19.09	13.1	25.2	42.57
PLAUR	NM_001005376.1	Endogenous	25.71	74.61	46.62	13.22	33.74	20.48	25.46	52.56	50.91	16.38	32.29	19.58
PMCH	NM_002674.2	Endogenous	8.26	712.41	434.78	11.19	1491.87	506.85	16.08	697.6	257.1	19.66	1313.58	315.87
PNMA1	NM_006029.4	Endogenous	501.33	392.31	411.46	831.07	734.08	602.42	379.18	344.02	408.57	475.04	679.63	561.93
POU2AF1	NM_006235.2	Endogenous	16.53	127.56	69.94	9.15	27.36	10.24	13.4	119.45	67.46	5.73	15.75	11.92
POU2F2	NM_002698.2	Endogenous	449	322.51	250.61	864.64	859.01	641.67	379.18	240.5	208.74	783	710.34	571.29
PPARG	NM_015869.3	Endogenous	33.05	81.83	156.19	18.31	10.03	20.48	33.5	119.45	179.46	12.29	10.24	12.77
PPBP	NM_002704.2	Endogenous	6.43	8.42	11.66	12.21	7.3	15.36	17.42	12.74	11.46	23.75	5.51	10.22
PRAME	NM_006115.3	Endogenous	5.51	7.22	11.66	1.02	7.3	4.27	17.42	17.52	11.46	5.73	3.15	5.11
PRF1	NM_005041.3	Endogenous	344.32	1524.71	576.98	511.66	1035.01	629.72	736.93	934.91	339.83	363.65	952.11	509.99
PRG2	NM_002728.4	Endogenous	51.42	62.58	38.47	48.83	38.3	52.9	26.8	39.82	38.18	67.16	32.29	47.68
PRKCD	NM_006254.3	Endogenous	188.23	400.73	278.58	259.39	279.04	372.88	168.82	307.39	254.56	181.01	257.52	243.5
PRKCE	NM_005400.2	Endogenous	51.42	71	54.78	36.62	32.83	29.01	30.82	36.63	63.64	25.39	32.29	33.2
PRM1	NM_002761.2	Endogenous	14.69	14.44	17.48	6.1	9.12	12.8	10.72	9.56	16.55	8.19	3.94	11.07
PSEN1	NM_000021.2	Endogenous	505.92	558.38	427.78	738.5	776.94	556.34	486.37	465.07	451.84	549.58	817.44	538.09
PSEN2	NM_000447.2	Endogenous	33.97	101.09	103.74	28.48	75.69	81.06	58.95	129.01	113.28	54.06	97.65	61.3
PSMB10	NM_002801.2	Endogenous	1172.53	1756.96	1671.5	1584.83	1867.57	1610.99	1234.03	1599.06	1578.26	1662.65	2000.29	1735.17
PSMB7	NM_002799.2	Endogenous	5997.62	3630.66	4624	5249.88	3338.47	4016.39	6503.76	4363.98	4490.4	5390.1	3387.9	3946.27
PSMB8	NM_004159.4	Endogenous	4877.43	7092.84	6325.81	5453.33	6113.38	6323.66	4132.18	6092.05	5406.81	5730	6024.5	6428.12
PSMB9	NM_002800.4	Endogenous	3902.31	5047.06	4547.07	5903.95	4901.46	4636.73	2734.69	3699.82	3100.51	3505.49	3651.72	3592.93
PSMD7	NM_002811.3	Endogenous	4989.45	4492.29	4514.44	4619.2	3651.25	4638.43	4252.77	4139.41	4333.85	4339.27	3693.45	4492.87
PTGDR2	NM_004778.1	Endogenous	5.51	13.24	19.82	8.14	15.5	7.68	25.46	25.48	17.82	8.19	7.88	8.51
PTGS2	NM_000963.1	Endogenous	21.12	109.51	208.65	12.21	34.65	44.37	24.12	179.97	302.92	16.38	44.89	28.1
PTPRC	NM_080921.3	Endogenous	4778.27	4565.7	3799.91	3673.19	5550.74	2982.22	3132.63	3757.16	2711.04	1996.82	4769.2	2845.4
PVR	NM_006505.3	Endogenous	89.06	166.07	228.46	23.4	60.19	143.35	128.63	211.83	241.83	45.05	81.9	99.61
PYCARD	NM_013258.3	Endogenous	9.18	60.17	47.79	13.22	75.69	46.93	18.76	44.6	33.09	13.92	91.35	60.45
RAG1	NM_000448.2	Endogenous	5.51	10.83	3.5	1.02	9.12	5.97	6.7	7.96	8.91	5.73	3.94	5.11
REL	NM_002908.2	Endogenous	819.94	403.14	335.7	799.54	693.96	947.14	825.36	344.02	320.74	704.37	518.97	728.8
RELA	NM_021975.2	Endogenous	167.11	156.44	132.88	249.22	186.94	142.5	186.24	114.67	137.46	212.13	152.78	167.73
RELB	NM_006509.2	Endogenous	648.24	610.12	553.67	1012.14	612.8	579.38	869.58	557.44	582.94	796.93	672.54	618.12
REPS1	NM_001128617.2	Endogenous	1083.47	1169.7	1137.64	833.1	921.02	952.26	1374.71	1255.04	1220.6	868.18	922.97	1060.85
RIPK2	NM_003821.5	Endogenous	206.59	49.34	58.28	121.05	106.69	113.49	162.12	43	45.82	105.66	107.89	93.65
ROPN1	NM_017578.2	Endogenous	18.36	14.44	22.15	10.17	15.5	19.63	24.12	20.7	12.73	14.74	14.96	14.47
RORA	NM_134261.2	Endogenous	881.46	652.24	369.5	679.5	989.41	790.99	935.23	522.4	458.2	492.24	915.09	641.11
RORC	NM_001001523.1	Endogenous	68.86	91.46	95.58	75.27	49.24	25.6	125.95	144.93	124.73	47.5	34.65	13.62
RPS6	NM_001010.2	Endogenous	46871.82	50773.82	47856.75	37219.12	45987.12	44690.57	54822.36	43308.47	46908.64	40634.99	46202.83	45740.95
RRAD	NM_004165.1	Endogenous	8.26	4.81	13.99	2.03	7.3	2.56	4.02	19.11	10.18	4.91	1.58	4.26
RUNX1	NM_001754.4	Endogenous	449	530.7	435.94	773.09	556.26	372.03	501.11	598.85	460.75	560.22	494.56	332.9
RUNX3	NM_004350.1	Endogenous	1209.26	1217.84	889.37	2247.04	1298.55	1309.79	1602.49	1054.36	930.41	2036.13	1266.33	1405.67
S100A12	NM_005621.1	Endogenous	8.26	3.61	3.5	9.15	3.65	5.12	10.72	11.15	12.73	8.19	7.88	4.26
S100A7	NM_002963.2	Endogenous	6.43	8.42	8.16	1.02	3.65	5.97	13.4	15.93	17.82	4.1	4.73	8.51
S100A8	NM_002964.3	Endogenous	5.51	2.41	4.66	7.12	6.38	5.97	9.38	9.56	10.18	7.37	3.94	1
S100B	NM_006272.1	Endogenous	3.67	8.42	10.49	4.07	5.47	4.27	12.06	11.15	8.91	7.37	6.3	5.96
SAA1	NM_199161.1	Endogenous	7.35	6.02	13.99	4.07	6.38	5.12	5.36	15.93	11.46	5.73	8.66	11.07
SBNO2	NM_014963.2	Endogenous	349.83	382.68	342.69	515.73	185.12	131.41	393.92	379.06	344.93	449.65	185.85	127.71
SELE	NM_000450.2	Endogenous	8.26	6.02	6.99	7.12	3.65	4.27	8.04	1.59	5.09	9.83	8.66	4.26
SELL	NR_029467.1	Endogenous	1162.43	315.29	420.79	2435.23	1592.18	1060.63	635.1	240.5	282.56	1440.69	1791.6	1810.09
SELPLG	NM_001206609.1	Endogenous	377.38	904.96	719.19	534.04	665.69	334.49	360.43	785.2	603.3	342.36	542.6	364.4
SEMG1	NM_003007.2	Endogenous	11.94	10.83	12.82	6.1	5.47	11.09	21.44	27.08	14	10.65	6.3	7.66
SERPINB2	NM_002575.1	Endogenous	14.69	12.03	15.15	12.21	14.59	5.97	25.46	15.93	16.55	33.58	7.09	5.11
SERPING1	NM_000062.2	Endogenous	5.51	4.81	1.17	5.09	9.12	7.68	1.34	1.59	1.27	2.46	3.94	1
SH2B2	NM_020979.3	Endogenous	11.02	31.29	15.15	14.24	17.33	34.98	14.74	31.85	19.09	18.02	33.86	24.69
SH2D1A	NM_001114937.2	Endogenous	719.86	2481.41	2248.48	561.51	973	489.78	1022.33	2172.43	2172.65	436.55	1160.8	773.93
SH2D1B	NM_053282.4	Endogenous	10.1	13.24	6.99	9.15	9.12	9.39	4.02	14.33	1.27	5.73	2.36	16.18
SIGIRR	NM_021805.2	Endogenous	56.01	101.09	128.22	151.57	143.17	81.91	108.53	100.34	95.46	81.9	202.39	95.36
SIGLEC1	NM_023068.3	Endogenous	3.67	10.83	17.48	7.12	14.59	10.24	17.42	15.93	14	9.01	15.75	9.37
SLAMF1	NM_003037.2	Endogenous	1371.78	969.94	846.24	1388.51	437.71	430.91	1164.35	759.71	734.4	1018.88	333.12	338.86
SLAMF6	NM_001184714.1	Endogenous	134.06	126.36	108.4	48.83	88.45	54.61	56.27	87.6	72.55	35.22	88.99	49.38
SLAMF7	NM_021181.3	Endogenous	36.73	38.51	79.26	15.26	33.74	66.56	48.24	15.93	36.91	28.67	17.33	28.95
SLC11A1	NM_000578.2	Endogenous	4.59	9.63	12.82	5.09	5.47	5.97	6.7	20.7	7.64	5.73	3.15	4.26
SMAD2	NM_005901.5	Endogenous	2331.29	2234.71	2431.48	1246.1	1242.01	1134.01	2468.05	2387.44	2870.14	1144.2	1156.07	1262.64
SMAD3	NM_005902.3	Endogenous	281.88	950.69	569.99	146.48	143.17	45.22	288.07	608.41	477.3	139.24	168.53	45.12
SMPD3	NM_018667.3	Endogenous	7.35	8.42	3.5	6.1	10.94	5.12	9.38	7.96	7.64	12.29	9.45	7.66
SOCS1	NM_003745.1	Endogenous	2461.67	3240.75	2673.93	5223.43	1823.8	1648.54	3057.6	3202.9	2751.77	5011.7	1895.55	1768.37
SPA17	NM_017425.3	Endogenous	51.42	125.15	127.05	30.52	152.29	127.14	64.31	122.64	128.55	45.05	167.74	169.43
SPACA3	NM_173847.3	Endogenous	12.85	125.15	137.54	31.53	94.84	69.12	12.06	111.49	175.64	23.75	48.83	59.6
SPANXB1	NM_032461.2	Endogenous	4.59	10.83	11.66	3.05	11.85	5.12	14.74	17.52	15.27	8.19	5.51	6.81
SPINK5	NM_006846.3	Endogenous	11.02	16.85	16.32	6.1	10.03	8.53	16.08	33.45	30.55	4.1	5.51	8.51
SPN	NM_003123.3	Endogenous	263.52	646.23	512.87	405.87	698.52	510.26	230.46	587.7	428.93	253.9	499.29	357.59
SPO11	NM_198265.1	Endogenous	36.73	64.98	64.11	61.03	54.71	48.64	26.8	58.93	40.73	45.05	47.25	46.83
SPP1	NM_000582.2	Endogenous	28.46	192.54	65.27	22.38	27.36	15.36	79.05	151.31	71.28	13.92	18.9	8.51
SSX1	NM_005635.2	Endogenous	6.43	12.03	6.99	3.05	8.21	11.09	6.7	12.74	12.73	3.28	7.88	7.66
SSX4	NM_005636.3	Endogenous	6.43	1.2	1.17	4.07	4.56	1	1.34	1.59	1.27	4.91	2.36	1
ST6GAL1	NM_003032.2	Endogenous	1594.9	1140.82	1063.04	1047.74	1260.25	735.53	1682.88	1081.44	882.04	974.66	1415.17	834.38
STAT1	NM_007315.2	Endogenous	2675.61	3025.35	2748.53	7097.16	6978.77	5123.1	3490.38	3429.07	2289.75	6999.51	8166.55	6302.96
STAT2	NM_005419.2	Endogenous	449	522.28	504.71	629.66	596.38	302.06	418.04	616.37	449.29	518.45	622.93	312.47
STAT3	NM_139276.2	Endogenous	3178.78	4807.58	3602.92	3947.84	4381.68	5045.45	4046.42	4011.99	3831.09	3960.87	4364.42	4996.9
STAT4	NM_003151.2	Endogenous	912.68	568	366	691.71	769.64	686.04	712.81	430.03	306.74	620.01	694.59	631.74
STAT5B	NM_012448.3	Endogenous	688.64	1557.2	1035.07	855.48	995.79	928.37	850.82	1278.93	1072.96	764.98	1067.09	948.47
STAT6	NM_003153.3	Endogenous	1725.28	1136.01	1249.54	1311.2	1539.29	1752.64	1567.65	1256.63	1434.43	1683.94	1575.03	1687.49
SYCP1	NM_003176.2	Endogenous	16.53	15.64	18.65	15.26	11.85	18.77	18.76	19.11	6.36	6.55	9.45	8.51
SYK	NM_003177.3	Endogenous	2.75	13.24	4.66	8.14	6.38	4.27	10.72	12.74	12.73	6.55	3.94	9.37
SYT17	NM_016524.2	Endogenous	8.26	6.02	1.17	2.03	5.47	8.53	10.72	15.93	12.73	4.91	9.45	2.55
TAB1	NM_153497.2	Endogenous	174.46	316.49	276.25	259.39	312.78	296.94	187.58	291.46	226.56	239.98	359.9	326.94
TAL1	NM_003189.2	Endogenous	7.35	6.02	3.5	13.22	3.65	11.95	9.38	4.78	2.55	4.1	2.36	5.11
TANK	NM_004180.2	Endogenous	1802.41	2932.68	2088.79	2267.39	2791.33	2697.22	2295.21	2683.69	1999.55	2000.91	2728.75	2607.01
TAP1	NM_000593.5	Endogenous	793.32	758.14	666.73	1075.2	620.09	643.37	970.07	823.42	665.67	1078.67	614.26	635.15
TAP2	NM_000544.3	Endogenous	1346.07	1097.5	1130.65	2090.39	948.38	1082.81	1464.48	1614.99	1230.79	2163.08	989.91	1169.83
TAPBP	NM_003190.4	Endogenous	1611.43	1948.3	1790.39	2023.25	2440.24	2252.66	1907.98	2083.24	1845.54	2128.68	2493.28	2171.94
TARP	NM_001003799.1	Endogenous	54.17	151.63	104.91	32.55	34.65	41.81	85.75	127.42	104.37	19.66	34.65	23.84
TBK1	NM_013254.2	Endogenous	370.95	549.95	402.14	481.15	529.81	454.8	332.29	430.03	319.47	384.13	492.2	418.89
TBX21	NM_013351.1	Endogenous	1178.04	2027.73	1811.37	2543.05	635.59	1020.52	1727.1	1544.91	1480.25	2211.41	594.58	937.4
TCF7	NM_003202.2	Endogenous	467.36	348.99	229.63	116.98	216.12	202.23	422.06	383.84	182.01	318.61	305.56	321.83
TFE3	NM_006521.3	Endogenous	48.66	40.92	24.48	48.83	43.77	41.81	33.5	54.15	33.09	45.05	48.83	36.61
TFEB	NM_007162.2	Endogenous	78.96	109.51	68.77	107.83	102.13	81.06	93.79	73.26	64.91	76.99	81.11	66.41
TFRC	NM_003234.1	Endogenous	8990.01	9468.35	9113.96	10730.67	6048.63	10229.13	11331.33	8559.12	8607.87	10084.01	6426.14	9784.36
TGFB1	NM_000660.3	Endogenous	1481.04	2382.73	1904.62	1923.57	1821.98	2026.54	1917.36	2306.22	2084.83	1680.67	1798.69	1823.71
TGFB2	NM_003238.2	Endogenous	1	2.41	1.17	1.02	8.21	1.71	1.34	3.19	2.55	1.64	2.36	3.41
THBD	NM_000361.2	Endogenous	11.02	10.83	16.32	12.21	17.33	17.07	24.12	15.93	20.36	14.74	10.24	14.47
THBS1	NM_003246.2	Endogenous	13.77	19.25	19.82	8.14	19.15	24.75	6.7	23.89	8.91	13.1	17.33	14.47
THY1	NM_006288.2	Endogenous	12.85	33.7	30.31	8.14	7.3	8.53	22.78	78.04	39.46	3.28	2.36	7.66
TICAM1	NM_014261.1	Endogenous	302.08	300.85	285.58	389.6	319.17	343.87	301.47	296.24	258.38	401.33	296.89	279.26
TICAM2	NM_021649.4	Endogenous	68.86	56.56	65.27	57.98	53.8	37.54	85.75	79.63	45.82	56.51	50.4	38.31
TIGIT	NM_173799.2	Endogenous	142.32	287.61	134.05	645.94	4474.69	2763.78	172.84	293.06	193.46	773.17	4894.42	3617.63
TIRAP	NM_148910.2	Endogenous	33.05	49.34	54.78	84.43	80.25	58.88	56.27	71.67	68.73	59.79	78.75	39.16
TLR1	NM_003263.3	Endogenous	19.28	49.34	51.29	19.33	100.31	57.17	45.56	55.74	42	18.84	81.11	46.83
TLR10	NM_030956.2	Endogenous	20.2	55.36	29.14	17.29	47.42	17.07	29.48	38.22	24.18	21.3	35.44	22.14
TLR2	NM_003264.3	Endogenous	23.87	14.44	16.32	55.95	29.18	40.1	26.8	22.3	20.36	66.34	37.8	43.42
TLR3	NM_003265.2	Endogenous	2.75	2.41	10.49	6.1	6.38	8.53	2.68	22.3	2.55	4.91	6.3	2.55
TLR4	NM_138554.2	Endogenous	5.51	6.02	5.83	4.07	5.47	1.71	1.34	4.78	7.64	3.28	1.58	1
TLR5	NM_003268.3	Endogenous	4.59	13.24	12.82	4.07	5.47	2.56	6.7	14.33	14	21.3	5.51	5.96
TLR6	NM_006068.2	Endogenous	37.65	36.1	30.31	24.41	257.16	110.07	26.8	30.26	17.82	18.02	208.69	123.45
TLR7	NM_016562.3	Endogenous	23.87	22.86	15.15	7.12	8.21	7.68	18.76	22.3	17.82	4.91	8.66	13.62
TLR8	NM_016610.2	Endogenous	7.35	2.41	5.83	3.05	6.38	4.27	14.74	17.52	6.36	10.65	3.15	7.66
TLR9	NM_017442.2	Endogenous	17.45	14.44	16.32	13.22	19.15	11.09	20.1	11.15	11.46	19.66	13.39	8.51
TMEFF2	NM_016192.2	Endogenous	4.59	12.03	3.5	8.14	7.3	5.12	9.38	9.56	10.18	4.1	7.09	5.96
TNF	NM_000594.2	Endogenous	16176.69	5051.87	5353.68	5568.27	1820.15	2935.28	15881.55	4873.64	5335.53	5958.51	1719.94	2457.16
TNFAIP3	NM_006290.2	Endogenous	3296.31	4189.03	2971.16	7476.58	3983.18	6169.22	3056.26	3056.38	2574.85	5150.94	3512.33	5437.08
TNFRSF10B	NM_003842.3	Endogenous	135.89	154.04	135.21	100.7	126.75	148.47	116.57	154.49	129.82	92.55	68.51	114.09
TNFRSF10C	NM_003841.3	Endogenous	6.43	13.24	23.31	5.09	11.85	8.53	16.08	20.7	20.36	8.19	3.15	6.81
TNFRSF11A	NM_003839.2	Endogenous	23.87	658.26	311.22	304.15	372.06	288.41	29.48	385.43	266.01	193.29	292.96	285.22
TNFRSF11B	NM_002546.2	Endogenous	11.94	18.05	18.65	5.09	7.3	9.39	49.58	39.82	21.64	5.73	5.51	7.66
TNFRSF12A	NM_016639.1	Endogenous	15.61	24.07	17.48	26.45	19.15	19.63	10.72	19.11	12.73	12.29	7.88	11.92
TNFRSF13B	NM_012452.2	Endogenous	35.81	6.02	15.15	9.15	16.41	7.68	14.74	17.52	5.09	17.2	8.66	16.18
TNFRSF13C	NM_052945.3	Endogenous	46.83	52.95	43.13	53.91	58.36	41.81	91.11	54.15	47.09	82.72	59.06	47.68
TNFRSF14	NM_003820.2	Endogenous	968.69	1281.62	1012.92	1315.27	1649.63	1264.56	911.12	1106.92	913.86	968.92	1558.5	1126.41
TNFRSF17	NM_001192.2	Endogenous	11.94	9.63	11.66	7.12	7.3	11.09	18.76	17.52	8.91	4.1	8.66	14.47
TNFRSF18	NM_004195.2	Endogenous	1765.68	1835.18	805.44	5151.21	2344.5	2711.73	2196.06	1540.13	910.04	2912.5	1571.88	1660.24
TNFRSF1A	NM_001065.2	Endogenous	373.7	389.9	363.67	305.17	412.18	278.17	333.63	375.88	322.02	276.02	364.62	275.86
TNFRSF1B	NM_001066.2	Endogenous	853	2535.56	1384.75	5156.3	10058.26	6417.52	1228.67	2134.21	1532.44	4226.24	8809.95	6365.11
TNFRSF4	NM_003327.2	Endogenous	4056.57	5239.6	3291.7	9714.47	5082.93	5699.91	6328.23	4865.67	3953.28	8166.64	4041.54	4452.85
TNFRSF8	NM_152942.2	Endogenous	345.24	3162.53	1806.71	1157.6	1533.82	1308.08	631.08	2414.52	1582.08	794.47	1026.13	870.99
TNFRSF9	NM_001561.4	Endogenous	1435.13	936.24	599.13	9436.77	1580.32	2723.67	1452.43	552.66	525.66	6731.68	1170.25	2039.97
TNFSF10	NM_003810.2	Endogenous	267.19	1596.91	1064.21	114.95	352.91	252.57	324.25	1822.04	720.4	117.94	456.76	277.56
TNFSF11	NM_003701.2	Endogenous	61.52	39.71	69.94	23.4	30.09	33.28	54.93	43	86.55	20.48	44.1	29.8
TNFSF12	NM_003809.2	Endogenous	177.21	444.05	307.72	246.17	455.95	317.42	143.37	420.47	372.93	198.21	444.95	355.89
TNFSF13	NM_003808.3	Endogenous	4.59	4.81	9.32	7.12	19.15	15.36	6.7	9.56	10.18	8.19	11.03	18.73
TNFSF13B	NM_006573.4	Endogenous	10.1	25.27	31.47	21.36	303.66	77.65	26.8	33.45	31.82	43.41	622.14	233.29

TABLE S1. Gene expression of stimulated T cells (cont.)														
TNFSF14	NM_003807.3	Endogenous	1330.46	542.73	716.85	725.28	772.38	929.22	1282.26	632.3	787.86	860.81	700.1	971.45
TNFSF15	NM_001204344.1	Endogenous	29.38	13.24	11.66	8.14	10.94	15.36	46.9	12.74	12.73	11.47	10.24	10.22
TNFSF18	NM_005092.2	Endogenous	1	6.02	1.17	8.14	3.65	5.97	4.02	3.19	5.09	3.28	3.15	7.66
TNFSF4	NM_003326.2	Endogenous	26.63	40.92	37.3	20.34	33.74	26.45	44.22	101.93	43.27	18.84	26.78	18.73
TNFSF8	NM_001244.3	Endogenous	2309.25	404.34	212.14	96.64	62.01	36.69	2344.78	703.97	215.1	69.62	41.74	46.83
TOLLIP	NM_019009.2	Endogenous	323.2	271.97	251.77	474.03	349.26	301.21	440.82	316.95	277.47	419.35	298.47	256.27
TP53	NM_000546.2	Endogenous	818.11	1324.94	1172.61	860.57	1308.58	1227.02	968.73	1301.23	1298.24	933.7	1482.11	1443.99
TPSAB1	NM_003294.3	Endogenous	10.1	9.63	18.65	14.24	18.24	13.65	24.12	11.15	19.09	13.1	11.03	6.81
TPTE	NM_199259.2	Endogenous	5.51	9.63	10.49	5.09	8.21	10.24	2.68	9.56	12.73	6.55	7.88	7.66
TRAF2	NM_021138.3	Endogenous	700.58	735.28	666.73	829.04	863.57	752.59	765.07	719.9	697.49	843.61	826.89	743.28
TRAF3	NM_145725.1	Endogenous	1896.06	1565.62	1187.76	3038.44	1519.23	1813.22	1729.78	1242.3	1122.6	2277.75	1522.27	1688.34
TRAF6	NM_145803.1	Endogenous	138.65	213	216.8	174.96	150.46	130.55	209.02	222.98	246.92	149.07	137.03	131.12
TREM1	NM_018643.3	Endogenous	1	1.2	1.17	1.02	31	34.98	1.34	1.59	1.27	1	47.25	9.37
TREM2	NM_018965.3	Endogenous	4.59	8.42	3.5	6.1	5.47	5.97	8.04	11.15	7.64	4.1	2.36	4.26
TTK	NM_003318.3	Endogenous	137.73	714.82	713.36	38.65	645.63	694.57	92.45	616.37	673.31	99.1	550.47	710.07
TXK	NM_003328.1	Endogenous	62.44	75.81	37.3	33.57	35.56	14.51	88.43	66.89	38.18	43.41	51.19	29.8
TXNIP	NM_006472.1	Endogenous	424.2	736.48	512.87	673.4	601.85	211.61	751.67	605.22	366.56	614.28	667.03	259.68
TYK2	NM_003331.3	Endogenous	373.7	588.46	488.39	464.87	622.83	627.16	365.79	621.15	470.93	447.2	645.76	573.85
UBC	NM_021009.3	Endogenous	23036.5	34908.21	28742.96	31212.43	32025.02	29927.11	28915.86	30211.77	27180.41	27678.61	29189.31	28955.48
ULBP2	NM_025217.2	Endogenous	22.95	7.22	15.15	25.43	6.38	4.27	16.08	23.89	19.09	23.75	8.66	6.81
USP9Y	NM_004654.3	Endogenous	87.23	113.12	123.56	90.53	108.52	95.57	97.81	152.9	147.64	82.72	101.59	99.61
VCAM1	NM_001078.3	Endogenous	6.43	8.42	8.16	7.12	8.21	3.41	8.04	15.93	8.91	4.91	1.58	3.41
VEGFA	NM_001025366.1	Endogenous	200.17	432.02	433.61	39.67	231.62	331.07	162.12	332.87	316.92	88.46	242.56	306.51
VEGFC	NM_005429.2	Endogenous	10.1	14.44	11.66	14.24	10.94	17.07	9.38	11.15	15.27	9.01	15.75	12.77
XCL2	NM_003175.3	Endogenous	305.76	90.25	96.75	24.41	60.19	64	273.33	133.79	42	31.12	15.75	47.68
XCR1	NM_005283.2	Endogenous	8.26	6.02	8.16	11.19	7.3	11.95	8.04	4.78	12.73	9.01	10.24	12.77
YTHDF2	NM_001172828.1	Endogenous	1031.13	1009.65	1152.8	1078.26	1184.56	1207.39	1357.29	1146.74	1202.79	1045.91	1221.44	1207.29
ZAP70	NM_001079.3	Endogenous	1276.29	1773.81	1610.88	1102.67	1575.76	1238.11	1189.81	1527.39	1505.71	1051.65	1531.72	1178.35
ZNF205	NM_001031686.1	Endogenous	1	1.2	2.33	7.12	8.21	3.41	5.36	1.59	8.91	8.19	4.73	3.41
ABCF1	NM_001090.2	Housekeeping	2037.47	1511.47	1517.63	1947.98	1338.67	1631.47	2186.68	1591.1	1608.81	2293.31	1513.61	1724.95
AGK	NM_018238.3	Housekeeping	1326.79	1835.18	1779.9	1031.46	877.25	1350.74	1554.26	1780.63	1793.36	1027.89	863.91	1196.23
ALAS1	NM_000688.4	Housekeeping	806.17	818.31	727.35	878.88	625.56	671.53	815.98	890.31	722.94	776.45	505.59	530.43
AMMECR1L	NM_001199140.1	Housekeeping	442.57	401.94	437.11	448.59	320.08	325.95	565.43	437.99	394.56	430	301.62	316.72
CC2D1B	NM_032449.2	Housekeeping	45.91	38.51	24.48	52.9	42.86	23.89	22.78	27.08	19.09	31.94	29.93	19.58
CNOT10	NM_001256741.1	Housekeeping	654.67	729.26	666.73	566.59	837.12	849.87	600.26	643.45	663.12	626.56	822.17	853.11
CNOT4	NM_001190848.1	Housekeeping	222.2	309.27	276.25	355.01	342.87	314.01	255.92	302.61	308.01	334.99	308.71	272.45
COG7	NM_153603.3	Housekeeping	224.04	257.53	221.47	191.24	188.76	197.11	239.84	294.65	280.01	192.47	245.71	240.1
DDX50	NM_024045.1	Housekeeping	891.56	1387.52	1159.79	856.5	1091.54	1244.94	899.06	1153.11	1200.24	877.19	1215.93	1322.23
DHX16	NM_001164239.1	Housekeeping	349.83	356.21	397.48	379.42	355.64	348.99	399.28	379.06	355.11	375.94	318.16	322.68
DNAJC14	NM_032364.5	Housekeeping	158.85	178.1	148.03	152.58	173.26	147.62	176.86	208.64	187.1	131.87	181.92	146.44
EDC3	NM_001142443.1	Housekeeping	800.66	770.18	742.5	735.45	836.21	831.95	834.74	829.79	756.04	798.56	853.67	852.26
EIF2B4	NM_172195.3	Housekeeping	1122.95	661.87	692.38	1123.01	692.13	692.86	1023.67	675.3	688.58	1008.24	642.61	704.96
ERCC3	NM_000122.1	Housekeeping	273.62	213	249.44	242.1	226.15	188.57	259.94	187.94	174.37	259.64	217.35	191.57
FCF1	NM_015962.4	Housekeeping	1943.81	1601.72	1698.3	1597.04	1619.53	1669.02	2112.98	1597.47	1719.54	1833.83	1667.96	1613.41
G6PD	NM_000402.2	Housekeeping	956.76	842.38	800.78	1496.33	709.46	893.38	1260.82	879.17	913.86	1365.34	796.97	830.97
GPATCH3	NM_022078.2	Housekeeping	65.19	39.71	69.94	113.93	68.39	75.09	68.33	44.6	63.64	97.47	66.94	66.41
GUSB	NM_000181.1	Housekeeping	171.7	435.63	368.34	159.7	308.22	316.57	191.6	428.43	445.48	172	363.05	332.9
HDAC3	NM_003883.2	Housekeeping	648.24	891.72	833.42	479.11	829.83	727.85	585.53	772.46	772.58	543.84	816.65	780.74
HPRT1	NM_000194.1	Housekeeping	2505.74	3501.89	3348.82	2025.29	2758.5	3265.5	2269.75	3225.2	3152.7	2094.28	3149.28	3373.27
MRPS5	NM_031902.3	Housekeeping	1915.35	2237.12	2058.48	1642.81	1459.95	1763.73	1972.3	2108.72	2133.19	1760.93	1524.63	1726.65
MTMR14	NM_022485.3	Housekeeping	505.92	497	398.64	606.26	547.14	416.4	515.85	496.92	434.02	502.07	504.8	519.36
NOL7	NM_016167.3	Housekeeping	2913.42	1719.66	1909.28	3049.63	1921.37	2156.24	2557.82	1689.85	1831.54	3135.28	1865.63	2244.31
NUBP1	NM_001278506.1	Housekeeping	245.16	206.98	214.47	259.39	258.98	285	209.02	202.27	199.83	217.05	226.8	210.3
POLR2A	NM_000937.2	Housekeeping	1015.52	1397.15	1501.32	1363.08	1149.91	1166.43	1318.44	1293.27	1438.25	1449.7	1082.84	1174.94
PPIA	NM_021130.2	Housekeeping	710.68	582.45	659.74	614.4	647.45	739.79	793.21	627.52	804.4	639.67	762.32	794.36
PRPF38A	NM_032864.3	Housekeeping	2019.1	1653.47	1869.65	1977.48	1731.7	1745.81	2358.18	1780.63	1801	2137.69	1771.13	1748.79
SAP130	NM_024545.3	Housekeeping	574.79	702.79	676.06	559.47	819.8	760.27	387.22	629.11	582.94	467.67	770.19	784.15
SDHA	NM_004168.1	Housekeeping	332.39	494.6	532.69	370.27	639.24	515.38	435.46	718.3	698.76	429.18	830.83	727.95
SF3A3	NM_006802.2	Housekeeping	1383.71	1416.4	1255.37	1024.34	1117.99	1237.26	1378.73	1286.89	1408.98	1219.55	1244.28	1430.36
TBP	NM_001172085.1	Housekeeping	907.17	758.14	756.49	811.74	673.89	734.67	852.16	702.38	712.76	867.36	681.2	723.7
TLK2	NM_006852.2	Housekeeping	381.97	484.97	441.77	430.28	430.42	411.28	467.62	519.22	458.2	369.39	452.82	423.15
TMUB2	NM_024107.2	Housekeeping	179.97	155.24	183	238.03	231.62	170.66	144.71	203.86	156.55	199.85	226.02	174.54
TRIM39	NM_021253.3	Housekeeping	134.06	97.48	103.74	210.56	146.82	148.47	164.8	70.08	94.19	214.59	160.65	148.14
TUBB	NM_178014.2	Housekeeping	7460.3	12145.91	12454.62	5565.22	11692.38	13626.89	7197.81	11308.11	12264.59	6677.63	12202.57	13969.02
USP39	NM_001256725.1	Housekeeping	942.98	876.07	979.12	868.71	817.06	940.32	909.78	901.46	894.77	938.62	873.36	1057.45
ZC3H14	NM_001160103.1	Housekeeping	659.26	557.17	543.18	497.42	791.53	768.81	640.46	567	498.93	523.37	685.93	734.76
ZKSCAN5	NM_014569.3	Housekeeping	49.58	33.7	62.94	65.1	88.45	52.9	46.9	49.37	58.55	65.52	61.43	57.04
ZNF143	NM_003442.5	Housekeeping	754.75	454.88	396.31	510.65	335.58	361.79	557.39	393.39	403.47	583.16	322.09	322.68
ZNF346	NM_012279.2	Housekeeping	107.43	131.17	114.23	94.6	139.52	145.91	88.43	127.42	138.73	111.39	155.14	130.27
NEG_A	ERCC_00096.1	Negative	19	7	14	10	13	6	10	13	7	10	19	5
NEG_B	ERCC_00041.1	Negative	16	7	13	8	14	14	12	13	17	9	10	17
NEG_C	ERCC_00019.1	Negative	6	10	18	7	17	22	12	7	17	7	14	18
NEG_D	ERCC_00076.1	Negative	8	5	6	2	8	10	7	6	9	8	2	3
NEG_E	ERCC_00098.1	Negative	12	16	14	7	13	11	12	3	12	14	10	8
NEG_F	ERCC_00126.1	Negative	8	5	5	6	15	13	6	6	11	14	17	12
NEG_G	ERCC_00144.1	Negative	2	3	4	5	7	4	1	2	2	6	8	1
NEG_H	ERCC_00154.1	Negative	5	6	4	2	9	10	3	5	5	1	8	8
POS_A	ERCC_00117.1	Positive	12400	11805	10943	14596	15926	15872	8421	8597	9966	18328	18541	15126
POS_B	ERCC_00112.1	Positive	3734	4032	3461	4612	4925	5028	2675	2646	3202	5556	5909	4794
POS_C	ERCC_00002.1	Positive	1103	1051	1023	1335	1402	1439	697	713	883	1610	1656	1387
POS_D	ERCC_00092.1	Positive	253	172	201	283	245	273	158	138	173	304	327	275
POS_E	ERCC_00035.1	Positive	52	51	59	65	62	64	49	33	51	73	93	66
POS_F	ERCC_00034.1	Positive	33	24	31	37	37	48	22	18	24	45	37	25

TABLE S2. arTreg culture well information for TCR sequencing

	Sample well*	Cell # (d0)	Cell # (d11)	Fold (d11/d0)
1	R1-sBc-arTreg-W1	1.00E+05	1.89E+06	18.9
2	R1-sBc-arTreg-W2	1.00E+05	1.75E+06	17.5
3	R1-sDC-arTreg-W1	1.00E+05	1.43E+06	14.3
4	R1-sDC-arTreg-W2	1.00E+05	1.63E+06	16.3
5	R1-sDC-arTreg-W3	1.00E+05	1.96E+06	19.6
6	R1-sDC-arTreg-W4	1.00E+05	1.66E+06	16.6
7	R2-sBc-arTreg-W1	1.00E+05	1.61E+06	16.1
8	R2-sBc-arTreg-W2	1.00E+05	1.59E+06	15.9
9	R2-sBc-arTreg-W3	1.00E+05	2.26E+06	22.6
10	R2-sBc-arTreg-W4	1.00E+05	2.00E+06	20
11	R2-sDC-arTreg-W1	1.00E+05	1.39E+06	13.9
12	R2-sDC-arTreg-W2	1.00E+05	1.72E+06	17.2
13	R3-sBc-arTreg-W1	2.50E+05	5.60E+06	22.4
14	R3-sBc-arTreg-W2	2.50E+05	4.90E+06	19.6
15	R3-sBc-arTreg-W3	2.50E+05	4.48E+06	17.9
16	R3-sDC-arTreg-W1	2.50E+05	4.90E+06	19.6
17	R3-sDC-arTreg-W2	2.50E+05	5.18E+06	20.7

* Sample well names indicate responder:stimulator combination (R1, R2, or R3), sBc-arTreg or sDC-arTreg, and culture well (W) number.

TABLE S3. Summary of sharing and distance analyses comparing digitally pooled sBc- and sDC-arTregs samples.

Comparison	digitally pooled well comparison				average of individual well comparisons			
	Shared unique CDR3	Shared CDR3 reads	Morisita distance	Jaccard distance	Shared unique CDR3	Shared CDR3 reads	Morisita distance	Jaccard distance
R1-sBc-arTreg vs R1-sDC-arTreg	13.00%	4.30%	0.80	0.87	6.84%	3.45%	0.94	0.93
R2-sBc-arTreg vs R2-sDC-arTreg	2.00%	0.24%	1.00	0.98	1.56%	0.28%	1.00	0.98
R3-sBc-arTreg vs R3-sDC-arTreg	15.00%	7.50%	0.94	0.85	8.78%	3.47%	0.98	0.91

TABLE S4. Antibodies used for flow cytometry

Antibody	Fluorophore	Clone	Vendor
CCR4	PE-Cy7	1G1	BD
CCR6	APC	11A9	BD
CCR7	BV711	G043H7	BioLegend
CCR9	BV421	L053E8	BioLegend
CD127	PE	HIL-7R-M21	BD
CD25	APC	M-A251	BD
CD25	Pacific Blue	M-A251	BioLegend
CD27	PE-Cy7	M-T751	BD
CD3	PE-Cy7	SK7	BD
CD4	AF700	RPA-T4	BD
CD4	PerCp-Cy5.5	RPA-T4	Tonbo
CD4	eVolve605	SK3	eBiosciences
CD58	BV421	1C3	BD
CD62L	FITC	SK11	BD
CD8	APC-Cy7	SK1	BD
CD8	BV711	RPA-T8	BioLegend
CD80	FITC	L307.4	BD
CD86	PerCp-Cy5.5	2331 (FUN-1)	BD
CXCR3	PerCp-Cy5.5	G025H7	BioLegend
FOXP3	e660	PCH101	eBiosciences
FOXP3	FITC	PCH101	eBiosciences
HELIOS	PE	22F6	BioLegend
HLA-ABC	APC	W6/32	BioLegend
HLA-DR	PE	G46-6	BD
IFNG	FITC	45.15	Beckman Coulter
IL-10	PerCp-Cy5.5	JES3-9D7	BioLegend
IL-17A	Pacific Blue	BL168	Beckman Coulter
IL-4	BV711	MP4-25D2	BD

4. Supporting References

1. Higdon LE, Lee K, Tang Q, Maltzman JS. Virtual Global Transplant Laboratory Standard Operating Procedures for Blood Collection, PBMC Isolation, and Storage: *Transplant Direct* (2016) 2:e101. doi:10.1097/TXD.0000000000000613

2. Putnam AL, Safinia N, Medvec A, Laszkowska M, Wray M, Mintz MA, Trotta E, Szot GL, Liu W, Lares A, et al. Clinical grade manufacturing of human alloantigen-reactive regulatory T cells for use in transplantation. *Am J Transplant* (2013) 13:3010–3020. doi:10.1111/ajt.12433

3. Noorchashm H, Lieu Y, Rostami S, Song H, Greeley S, Bazel S, Barker C, Naji A. A direct method for the calculation of alloreactive CD4+ T cell precursor frequency. *Transplantation* (1999) 67:1281-4. doi:10.1097/00007890-199905150-00015
